# High-throughput genotyping assays for identification of glycophorin B deletion variants in population studies

**DOI:** 10.1177/1535370220968545

**Published:** 2020-12-16

**Authors:** Dominic SY Amuzu, Kirk A Rockett, Ellen M Leffler, Felix Ansah, Nicholas Amoako, Collins M Morang’a, Christina Hubbart, Kate Rowlands, Anna E Jeffreys, Lucas N Amenga-Etego, Dominic P Kwiatkowski, Gordon A Awandare

**Affiliations:** 1 West African Centre for Cell Biology of Infectious Pathogens, University of Ghana, Accra, GH 0233, Ghana; 2 Department of Biochemistry, Cell and Molecular Biology, University of Ghana, Accra, GH 0233, Ghana; 3 Wellcome Centre for Human Genetics, Nuffield Department of Medicine, University of Oxford, Oxford OX3 7BN, UK; 4 Wellcome Sanger Institute, Hinxton CB10 1SA, UK; 5 Department of Human Genetics, Eccles Institute of Human Genetics, University of Utah, Salt Lake City, UT 84112-5330, USA; 6 Big Data Institute, Nuffield Department of Medicine, University of Oxford, Oxford OX3 7FZ, UK

**Keywords:** Glycophorins, malaria, *Plasmodium*, red blood cell, invasion, *GYPB* deletion

## Abstract

Glycophorins are the most abundant sialoglycoproteins on the surface of human erythrocyte membranes. Genetic variation in glycophorin region of human chromosome 4 (containing *GYPA*, *GYPB*, and *GYPE* genes) is of interest because the gene products serve as receptors for pathogens of major public health interest, including *Plasmodium* *sp.*, *Babesia* *sp.*, Influenza virus, *Vibrio cholerae* El Tor Hemolysin, and *Escherichia coli*. A large structural rearrangement and hybrid glycophorin variant, known as *Dantu*, which was identified in East African populations, has been linked with a 40% reduction in risk for severe malaria. Apart from *Dantu*, other large structural variants exist, with the most common being deletion of the whole *GYPB* gene and its surrounding region, resulting in multiple different deletion forms. In West Africa particularly, these deletions are estimated to account for between 5 and 15% of the variation in different populations, mostly attributed to the forms known as DEL1 and DEL2. Due to the lack of specific variant assays, little is known of the distribution of these variants. Here, we report a modification of a previous *GYPB* DEL1 assay and the development of a novel *GYPB* DEL2 assay as high-throughput PCR-RFLP assays, as well as the identification of the crossover/breakpoint for *GYPB* DEL2. Using 393 samples from three study sites in Ghana as well as samples from HapMap and 1000 G projects for validation, we show that our assays are sensitive and reliable for genotyping *GYPB* DEL1 and DEL2. To the best of our knowledge, this is the first report of such high-throughput genotyping assays by PCR-RFLP for identifying specific *GYPB* deletion types in populations. These assays will enable better identification of GYPB deletions for large genetic association studies and functional experiments to understand the role of this gene cluster region in susceptibility to malaria and other diseases.

## Impact statement

Glycophorins are of interest because they serve as receptors for pathogens including *Plasmodium* *sp*.*, Babesia* *sp*., Influenza virus, *Vibrio cholerae* El Tor Hemolysin, and *Escherichia coli*. Variation in the genes encoding these receptors may be important in influencing disease susceptibility. Due to high sequence homology (∼96) between glycophorins A, B, and E genes, it is challenging designing assays to genotype specific variations in the three genes. This work reports the development of two separate high-throughput assays for reliably genotyping glycophorin B deletions (DEL1 and DEL2) on large-scale. This is important for population prevalence studies and identification of affected individuals for investigating the functional effects of the gene variation. Furthermore, the work identified the location of the breakpoint for *GYPB* DEL2, which is important for understanding the gene cluster and the deletion mechanisms.

## Introduction

Malaria is still an important public health issue worldwide and the leading cause of death among children in sub-Saharan Africa (sSA). It is estimated that every year about 216 million cases of malaria and 445,000 deaths occur globally, with sSA being the most affected.^1^ *Plasmodium falciparum*, which is responsible for most of these deaths, has evolved complex machinery for invading erythrocytes. The mechanism is mediated by multiple redundant parasite ligands and specific human host receptors on the surface of erythrocytes to facilitate invasion.^2–4^ Many studies have shown that host-pathogen factors influence malaria outcomes and most certainly the parasite has affected the evolution of the human genome over the years.^5,6^ Several genetic host factors from single nucleotide polymorphisms to large structural variants are known to influence an individual’s susceptibility or resistance to malaria.^7–10^

Glycophorins (GYP) are glycosylated sialoglycoproteins found on the surface of human and animal erythrocytes.^11^ Human GYPA and GYPB are determinants of the major MNS blood group system while GYPC and GYPD are determinant of the Gerbich Blood Group. Some of these glycophorins have also been shown to be receptors on erythrocytes use by the *P. falciparum* to invade these cells. In addition, these glycophorins serve as receptors for other pathogens such as *Babesia sp.*, Influenza virus, encephalomyocarditis virus, *Vibrio cholerae* El Tor Hemolysin, and *E. coli.*^11–15^ These include GYPA which interacts with the *P. falciparum* protein erythrocyte binding antigen (EBA)-175,^16–18^ GYPB which interacts with erythrocyte binding ligand 1 (EBL-1),^19,20^ and GYPC which interacts with EBA-140, all in a sialic acid-dependent manner.^21–23^ The *GYPE* gene is not known to be expressed as a protein on the erythrocyte surface.^24^

**Figure 1.**
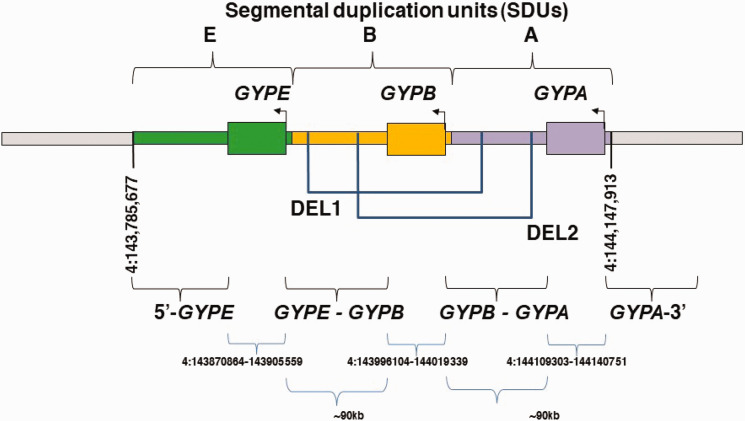
Schematic diagram of the human reference *GYP* gene region on chromosome 4. The three *GYP* segmental duplication units (SDUs) are indicated by different colors. The *GYP* gene-region boundary-locations and genes are shown with respect to GRCh37. The approximate locations of the DEL1 and DEL2 deletions are shown. Intergenic region names used in the main text are shown.^9^

The genes *GYPE, GYPB,* and *GYPA* are located in a gene cluster on the long arm of chromosome 4 (4q28-q31) approximately 360 kb long with each gene segmental duplication unit (SDU) spanning ∼120 kb comprised of a gene region of ∼30 kb and an intergenic region of ∼90 kb (Figure 1). *GYPC* and *GYPD* are located on chromosome 2 and are not discussed here. The *GYPA, GYPB,* and *GYPE* genes are evolutionarily related, with at least 95% sequence homology between them resulting from duplication events, whereby *GYPB* evolved from *GYPA,* and *GYPE* evolved from *GYPB.*^9,25,26^

Recently, a large structural variant in the chromosome 4 *GYP* region that gives rise to the *Dantu* glycophorin (DUP4) has been associated with about a 40% reduction in risk for severe malaria.^9,27^ Interestingly, this *Dantu* variant was found predominantly in East African populations, but there are many other common structural variants across all the West and East African populations that have been studied.^9^ The most common variants identified were deletions of the whole *GYPB* gene and the surrounding region known as *GYPB* DEL1 and *GYPB* DEL2 (Figure 1). However, little research has been conducted on these variants and their population distributions due to the lack of high throughput methods for genotyping these structural variants. With the difficulties in screening for these deletions and other variants, there is also lack of functional data on the effect of *GYPB* deletions on erythrocyte invasion, the growth of *P. falciparum*, and the changes that occur on the surface of the erythrocytes with respect to protein expression. Here, we show the development of two separate high-throughput assays for reliably detecting and genotyping *GYPB* deletions DEL1 and DEL2 that can be used to determine their distribution in populations and identify phenotypes functional investigations of these deletions on *P. falciparum* erythrocyte invasion and growth. Using data from 393 samples from different ethnic populations in southern Ghana as well as DNA samples from the HapMap and 1000 G projects, we show the development of high throughput assays for *GYPB* DEL1. We also DEL2 and show that these are sensitive and reliable for screening population samples with little interference from other glycophorin structural variants.

## Materials and methods

### Location of putative breakpoints for the GYPB whole-gene deletions DEL1 and DEL2

The breakpoints for DEL1 have previously been located on GRCh37 at chr4:144835160–144835280 (4:143914007–143914127 in GRCh38) in the 3ʹ region of the *GYPE* unit of the SDU, and chr4:144945398–144945517 (4:144024245–144024364 in GRCh38) in the 3ʹ region of the *GYPB* unit of the SDU,^9^ while the predicted location of the DEL2 breakpoint was given as 206,000 bases from the 5ʹ end of the *GYP* region (GRCh37:4:144706830, GRCh38:4:143785677).^9^ We downloaded 4 kb of sequence surrounding the DEL1 or DEL2 putative breakpoint coordinates for each of the *GYPE*, *GYPB*, and *GYPA* SDUs, from Ensembl (http://grch37.ensembl.org/Homo_sapiens/). The sequences were aligned using Clustal Omega (www.ebi.ac.uk/Tools/msa/clustalo/) and manually finished where required (Figure 1 and Supplementary Files 1 and 2). While initial designs were made using GRCh37, we have since compared our analysis with GRCh38 and provided coordinates with respect to GRCh38 or both where appropriate.

### Assay design for DEL1 and DEL2 putative breakpoint regions

**Figure 2.**
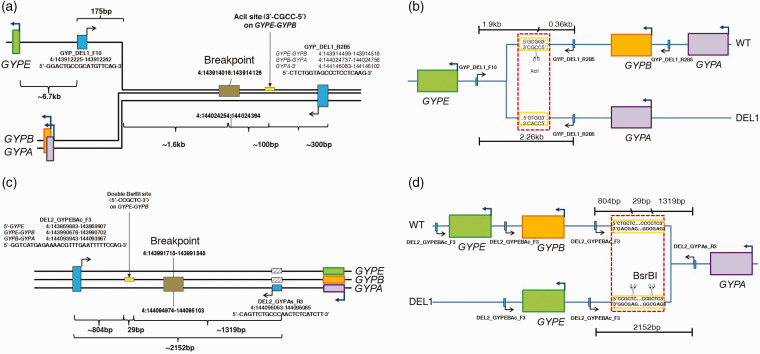
Schematic representation of strategies for amplifying and testing for the *GYPB* DEL1 and DEL2 structural variants. (a) Schematic representation of the alignment for the *GYP* SDUs showing the location of PCR primers (blue rectangles), putative breakpoint (gold rectangle), and AciI restriction site (yellow rectangle). The forward primer GYP_DEL1_F10 is specific to upstream of *GYPE* in the *GYPE-GYPB* region. The reverse primer GYPB_DEL1_R2B5 binds to the upstream of the *GYPB* gene in the *GYB-GYPA* region. In a normal or wild type individual, the GYPB_DEL1_R2B5 in the *GYPE-GYPB* region and the GYP_DEL1_F10 forward primer forms a PCR product made of sequences in the *GYPE_GYPB* region. In the *GYPB* DEL1 state, the PCR product formed is made of sequences in the *GYPE* and *GYPA* region because the *GYPB* is deleted. (b) Alternate schematic representation of the *GYPB* DEL1 RFLP assay showing a normal chromosome and the *GYPB* DEL1 chromosomes aligned. Genes (green, orange, and purple rectangles) and primers (blue rectangle) are indicated as well as the AciI restriction site and PCR-digestion fragment lengths. (c) Schematic representation of the alignment for the *GYP* SDUs showing the location of PCR primers, putative breakpoint (gold rectangle), and BsrBI restriction site (yellow rectangle). The forward primer GYP_DEL2_F3 is common to the *GYPA* downstream of the *GYPB-GYPA* region. The reverse primer GYPB_DEL2_R3 specifically binds to the upstream of the *GYPE* gene in the *GYPE-GYPB* region. In a normal or wild type individual, the GYPB_DEL2_F3 in the *GYPB-GYPA* region and the GYP_DEL2_R3 primer forms a PCR product made of sequences in the *GYPB_GYPA* region. In the *GYPB* DEL2 state, the PCR amplicon formed is made of sequences in the *GYPE* and *GYPA* region because the *GYPB* is deleted. (d) Alternate schematic of the *GYPB* DEL2 RFLP assay showing a normal chromosome and the *GYPB* DEL2 chromosomes aligned. Genes (green, orange, and purple rectangles) and primers (blue rectangle) are indicated as well as the BsrBI restriction site (red dotted rectangular area) and PCR-digestion fragment lengths. Coordinates of sequences are given with respect to GRCh38.

We developed a PCR-RFLP version of the published DEL1 assay^9^ using a similar primer strategy. A forward primer was positioned in the unique sequence 3ʹ to the *GYPE* gene with a common reverse primer positioned in the *GYPE-GYPB* and *GYPB-GYPA* regions (Figure 2(a) and 2(b)) but placed to generate a shorter PCR amplicon (∼2 kb) than that published. From the human genome reference sequence alignments (Supplementary File 1) of the equivalent SDUs, a restriction enzyme (AciI) site was identified that distinguished between the wild-type and DEL1 sequences (Figure 2(a) and (b)).

The assay for *GYPB* DEL2 used a strategy similar to that of the *GYPB* DEL1 assay, but with a unique primer such that at the *GYPA* end of the sequence (reverse primer) and a common primer placed at the 5ʹ end (forward primer) (Figure 2(c) and (d)). From the human genome reference sequence alignments (Supplementary File 2) of the equivalent SDUs, a restriction enzyme (BsrBI) site was identified that distinguished between the wild-type and *GYPB* DEL2 sequences (Figure 2(c) and (d)). For both *GYPB* DEL1 and DEL2, the restriction enzymes used were non-palindromic and therefore strand oriented (Table 1, Supplementary Files 2 to 5).

### Generation of *GYPB* variant control DNA

Cell lines from the 1000 G and associated projects with known *GYPB* deletions or wild-type (identified from the Leffler *et al*.^9^ study and Table 2) were identified and these cell-lines or their genomic DNA were sourced from the NHGRI repository (Coriell Institute, NY, USA [https://www.coriell.org/1/Browse/Biobanks]).

### *GYPB* DEL1 and DEL2 PCR conditions and restriction digest

 QIAGEN Fast Cycling PCR Kit (Qiagen, UK) was used for both the *GYPB* DEL1 and DEL2 PCR reactions as detailed in Table 3a and 3b, with primers purchased from IDT (Leuven, Belgium). Restriction enzymes AciI (Catalog number: R0551L, NEB, UK) and BsrBI (Catalog number: R0102L, NEB, UK) were purchased (Table 4a). Reactions were prepared in 96-well plates (#AB-800, ThermoFisher Scientific, UK) and cycled on an MJ Tetrad (BioRad, UK) as described in Table 3a and 3b. The PCR products were digested with the relevant restriction enzymes (NEB, UK) for 2 hours at 37°C and then the digestion fragments were separated on 1% agarose gel electrophoresis containing ethidium bromide (3 ng/uL) for 2–2½ hours. Products were visualized under UV light and photographed to allow genotype assignment. All plates contained control samples obtained from the NHGRI repository (Table 2).

### Genotyping cell lines

We tested the DEL1 and DEL2 assays on several cell lines (Table 2) that were identified from whole genome sequence analysis as having different *GYP* variants^9^ to check for cross-reactions or aberrant products.

### Screening for *GYP* Dantu (DUP4)

Samples were screened for the glycophorin variant *Dantu* (DUP4) using the assay described by Leffler *et al*.^9^

### Ethical approval and screening population in Ghana for *GYPB* DEL1 and DEL2

Ethical approval for two ongoing studies on glycophorins and malaria was granted by the Ethics Committee for Basic and Applied Sciences, College of Basic and Applied Sciences, University of Ghana (CPN: ECBAS 037/18–19), and the Noguchi Memorial Institute for Medical Research IRB, University of Ghana (CPN 004/11–12). Written informed consent was obtained from all the study participants or their parents/guardians in the case of the children. The assays developed were used to genotype DNA samples obtained from volunteers who had been enrolled in various ongoing studies in three areas of Ghana namely: Accra, Kintampo, and Hohoe.

Venous blood samples were collected and following curation of self-reported ethnicity to only include individuals of Ghanaian origin, comprised; Kintampo (*n* = 147), Hohoe (*n* = 43), and Accra (*n* = 203) (Figure 6, Table 5). Genomic DNA was extracted using the Qiagen QIAmp Blood Mini Kit or Chelex-100 as described for different batches of samples.^28^ The DNA samples were quantified using Picogreen as described above. The gDNA samples were diluted to 20 ng/µL and stored in 96-well PCR plates at −20°C until ready for genotyping. *GYPB* DEL1 and DEL2 genotyping assays were undertaken as given in Tables 3 and 4.

**Table 1.** Samples selected for testing and sequencing to identify the breakpoints for *GYPB* DEL1 and DEL2.

Use	GYP Target	Primers	Sequence (5'-3')	Dir	GC (%)	T_m_ (^o^C)	GYP Region	GRCh38 Location	GRCh38 Location
PCR/sequencing	DEL1	GYP_DEL1_F10	GGACTGCCGCATGTTCAG	Fwd	61	53	*GYPE*-*GYPB*	4:144833378-144833395	4:143912225-143912242
PCR/sequencing	DEL1	GYP_DEL1_R2B5	CTCTGGTAGCCCTCCTCAAG	Rev	60	56	*GYPE*-*GYPB*	4:144835652-144835671	4:143914499-143914518
							*GYPB-GYPA*	4:144945890-144945909	4:144024737-144024756
							GYPA-3'	4:145067236-145067255	4:144146083-144146102
PCR/sequencing	DEL2	DEL2_GYPEBAc_F3	GGTCATGAGAAAACGTTTGAATTTTCCAG	Fwd	37.9	59	5'-*GYPE*	4:144791036-144791060	4:143869883-143869907
							*GYPE*-*GYPB*	4:144911831-144911855	4:143990678-143990702
							*GYPB-GYPA*	4:145015096-145015120	4:144093943-144093967
PCR/sequencing	DEL2	DEL2_GYPBAs_R3	CAGTTCTGCCAACTCTCATCTT	Rev	45	56	*GYPB-GYPA*	4:145017216-145017238	4:144096063-144096085
Sequencing	DEL2	DEL2_BP_seq_Rev1	CTATGGGTCCCTCTCTGTGGA	Rev			5'-*GYPE*	4:144792394-144792414	4:143870624-143870648
							*GYPE*-*GYPB*	4:144913188-144913208	4:143991419-143991443
							*GYPB-GYPA*	4:145016443-145016458	4:144094684-144094708
Sequencing	DEL2	DEL2_BP_seq_Fwd	CATGTCTCACATCCAGTTAATGCTG	Fwd			5'-*GYPE*	4:144791777-144791801	4:143871241-143871261
							*GYPE*-*GYPB*	4:144912572-144912596	4:143992035-143992055
							*GYPB-GYPA*	4:145015837-145015861	4:144095290-144095305

Note: Primers were designed using Primer3 and purchased from IDT (see methods).

Alignments are shown in the *GYP* region. One primer from each PCR (GYP_DEL1_F10 and DEL2_GYPBAs_R3) was unique to the *GYPB* DEL1 and *GYPB* DEL2 breakpoints respectively while the other primers were designed against homologous sequence (GYP_DEL1_R2B5 and DEL2_GYPEBAc_F3).

Two primers were designed to internal regions of the *GYPB* DEL2 amplicon to aid with sequencing (DEL2_BP_seq_REV1 and DEL2_BP_seq_FWD)

**Table 2.** Samples selected testing and sequencing to identify DEL1 and DEL2 Breakpoints

Coriell Identifier*	Population Code	Population	Country	Genotype	DEL1	DEL2
HG00097	GBR	British	UK	N/N	II	II
GM06985	CEU	CEPH	USA	N/N	II	II
GM06986	CEU	CEPH	USA	N/N	II	II
GM06994	CEU	CEPH	USA	N/N	II	II
GM18522	YRI	Yoruba	Nigeria	N/N	II	II
GM19140	YRI	Yoruba	Nigeria	N/N	II	II
GM19141	YRI	Yoruba	Nigeria	N/N	II	II
GM19152	YRI	Yoruba	Nigeria	N/N	II	II
GM18523	YRI	Yoruba	Nigeria	DEL1/N	DI	II
GM19207	YRI	Yoruba	Nigeria	DEL1/N	DI	II
GM19223	YRI	Yoruba	Nigeria	DEL1/N	DI	II
HG02464	GWD	Mandinka	Gambia	DEL1/DEL1	DD	II
HG02545	ACB	Afro-Caribbean	Barbados	DEL1/DEL1	DD	II
HG03072	MSL	Mende	Sierra Leone	DEL1/DEL1	DD	II
HG03139	ESN	Essan	Nigeria	DEL1/DEL1	DD	II
GM18519	YRI	Yoruba	Nigeria	DEL1/DEL1	DD	II
GM18856	YRI	Yoruba	Nigeria	DEL2/N	II	DI
GM19144	YRI	Yoruba	Nigeria	DEL2/N	II	DI
GM17125	ASW	African-American	USA	DEL2/N	II	DI
HG03385	MSL	Mende	Sierra Leone	DEL2/DEL2	II	DD
GM20867	GIH	Gujarati Indian	USA	DEL14/N	XX	II
GM18858	YRI	Yoruba	Nigeria	DEL17/N	XX	XX
HG02586	GWD	Mandinka	Gambia	DUP1/N	XX	II
HG02588	GWD	Mandinka	Gambia	DUP1/N	II	II
GM18502	YRI	Yoruba	Nigeria	DUP1/N	II	XX
GM18870	YRI	Yoruba	Nigeria	DUP1/N	XX	II
HG02250	CDX	Dai	China	DUP2/N	II	II
HG02798	GWD	Mandinka	Gambia	DUP2/N	II	II
GM18552	CHB	Han	China	DUP2/N	II	II
GM18593	CHB	Han	China	DUP2/N	XX	II
GM18605	CHB	Han	China	DUP2/N	II	II
GM12829	CEU	CEPH	USA	DUP23/N	XX	XX
GM12249	CEU	CEPH	USA	DUP28/N	II	II
HG02554	ACB	Afro-Caribbean	Barbados	DUP4/N	II	II
HG02585	GWD	Mandinka	Gambia	DUP6/N	II	II
GM18545	CHB	Han	China	TRP1/N	XX	II
GM18620	CHB	Han	China	TRP1/N	XX	II
GM12341	CEU	CEPH	USA	TRP13/N	II	II
GM11894	CEU	CEPH	USA	TRP5/N	XX	II
GM18852	YRI	Yoruba	Nigeria	UNK	XX	II
GM19221	YRI	Yoruba	Nigeria	UNK	XX	II
**Ghana Identifier^§^**						
GX0387		Ghana	Ghana	N/N	II	II
GX0540		Ghana	Ghana	N/N	II	II
GX0600		Ghana	Ghana	N/N	II	II
GX0610		Ghana	Ghana	N/N	II	II
GX0531		Ghana	Ghana	N/N	II	II
GX0258		Ghana	Ghana	DEL1/DEL1	DD	II
GX0458		Ghana	Ghana	DEL1/DEL1	DD	II
GX0537		Ghana	Ghana	DEL1/DEL1	DD	II
GX0403		Ghana	Ghana	DEL2/DEL2	II	DD
GX0440		Ghana	Ghana	DEL2/DEL2	II	DD
GX0300ǂ		Nigeria	Nigeria	DEL1/DEL2	DI	DI

Note: A set of 1000G samples obtained from the NHGRI repository at the Coriell Institute, NY, USA, and with known *GYP* variation status of *GYPB* DEL1 homozygote or DEL2 homozygote (Leffler et al., 2017) were selected for sequencing. A second set of samples, selected from the Ghana sample collection described in this manuscript, were also sequenced following *GYPB* DEL1 or DEL2 status identification from the RFLP assays described in this manuscript.

N/N; wild-type or at least not *GYPB* DEL1/DEL2 positive; *GYPB* DEL1/DEL1: DEL1 homozygous; *GYPB* DEL2/DEL2: DEL2 homozygous; *GYPB* DEL1/DEL2: Heterozygous

*Coriell identifier for 1000G and HapMap samples (https://www.coriell.org/1/Browse/Biobanks)

^§^Ghana identifier is an anonymised identifier with no relationship to local identifiers.

^ǂ^This individual was collected at the Accra study site and was later found to be of Nigerian origin.

**Table 3.** PCR assay conditions for detecting *GYPB* DEL1 and DEL2

A: PCR Reaction Components		Volume (µL)			
Reagent	Stock	DEL1		DEL2	
gDNA Template	20 ng/µl	1		1	
Forward Primer	10 µM	0.25		0.75	
Reverse Primer	10 µM	0.25		0.25	
Qiagen Fast Cycling PCR Kit	2X	2.5		2.5	
MilliQ Water	-	7		6.5	
Total	-	11.00		11.00	

B: PCR Cycling Conditions		DEL1		DEL2	
PCR Step	Description	Temp (°C)	Time	Temp (°C)	Time
1	Initial Denaturation	95	5 min	95	5 min
2	Denaturation	96	5 sec	96	5 sec
3	Annealing	59	6 sec	55	6 sec
4	Extension	68	3 min 15 sec	68	3 min 15 sec
5	Repeats	Step 2, x39		Step 2, x39	
6	Final extension	72	1 min	72	1 min
7	End	15	4 min	15	4 min

A: PCR volumes and concentration for amplifying the *GYPB* DEL1 and DEL2.

B: PCR conditions for the amplifying the *GYPB* DEL1 and DEL2.

**Table 4.** Restriction Digest Conditions for Detecting *GYPB* DEL1 and DEL2

A: Restriction Digest conditions		Volume (µL)	
Reagent	Stock	DEL1	DEL2
Enzyme	10U/µL	0.2	0.2
Cat smart buffer	10x	1	1.2
Water	-	3.8	5.6
PCR reaction	-	5	5
Total Reaction Vol (µL)	-	10	12

**B: Restriction Digest Products**			
Genotype		DEL1 (AciI Enzyme)	DEL2 (BsrBI Enzyme)
Recognition site		5'-CCGC-3'	5'-CCGCTC-3'
Product code		NEB: R0551L	NEB: R0102L
Non-DEL1-DEL2 Sample		(3, 7, 29, 38), 1921, 296	804, 29, 1318
Homozygous Sample		(3, 7, 29, 38), 2225	2141
Heterozygous Sample		(3, 7, 29, 38), 1921, 296, 2225	804, 29, 1318, 2141

Note: 2 hours digest at 37°C then 5 minutes at 65°C to inactivate the enzyme (optional). Add 5 µL loading gel to the full reaction. Load 10 µL onto a 1% agarose gel with ethidium bromide.

100 V for 2 - 2½ hours using Bioline Hyperladder 1 kB (BIO-33053).

**Table 5.** Details of Sample Collection from 3 Study Sites in Ghana

Location	Gender	Age (years)		Ethnicity							
		Mean	range	Akan	Ewe	Ga	Gurunsi	Konkomba	Mo	Other	Total
Accra		5.44 ± 4.18	1-15	78	48	63	5	0	0	9	203
	Female	5.15 ± 4.21	1-15	48	22	30	4	0	0	3	107
	Male	5.67 ± 4.14	1-15	30	26	33	1	0	0	8	98
Hohoe		Adults	>18	3	35	2	1	0	0	2	43
	Female	Adults	> 18	2	21	0	1	0	0	2	26
	Male	Adults	> 18	1	14	2	0	0	0	0	17
Kintampo		3.25 ± 2.85	1-15	17	2	0	24	20	21	63	147
	Female	3.39 ± 3.72	1-12	5	2	0	9	8	13	24	61
	Male	3.15 ± 2.93	1-15	12	0	0	15	12	8	39	86
Overall		4.52 ± 3.83		98	85	65	30	20	21	76	393

Note: The six named ethnic groups were represented by more than 20 samples in the full sample set.

**Table 6.** Genotypes for DEL1 and DEL2 in Ghana

	Accra						Hohoe						Kintampo						Overall					
	II	DI	DD	XX	N	%	II	DI	DD	XX	N	%	II	DI	DD	XX	N	%	II	DI	DD	XX	N	%
DEL1 Group																								
Akan	63	11	0	4	78	7.43	3	0	0	0	3	0.00	14	0	0	3	17	0.00	80	11	0	7	98	6.04
Ewe	38	4	0	6	48	4.76	27	3	0	5	35	5.00	1	0	0	1	2	0.00	66	7	0	12	85	4.79
Ga	51	5	1	6	63	6.14	2	0	0	0	2	0.00	NA	NA	NA	NA	NA	NA	53	5	1	6	65	5.93
Gurunsi	4	0	0	1	5	0.00	1	0	0	0	1	0.00	22	1	0	1	24	2.17	27	1	0	2	30	1.79
Konkomba	NA	NA	NA	NA	NA	NA	NA	NA	NA	NA	NA	NA	17	1	0	2	20	2.78	17	1	0	2	20	2.78
Mo	NA	NA	NA	NA	NA	NA	NA	NA	NA	NA	NA	NA	16	2	0	3	21	5.56	16	2	0	3	21	5.56
Other	7	1	0	1	9	6.25	1	0	0	1	2	0.00	49	7	2	5	63	9.48	58	9	2	7	74	8.96
Total (N)	163	21	1	18	203	6.22	34	3		6	43	4.05	119	11	2	15	147	5.68	317	36	3	39	393	5.79
DEL2 Group																								
Akan	73	1	0	4	78	0.68	2	1	0	0	3	16.67	14	2	0	1	17	6.25	89	4	0	5	98	2.15
Ewe	44	1	0	3	48	1.11	30	3	0	2	35	4.55	2	0	0	0	2	0.00	76	4	0	5	85	2.50
Ga	52	3	0	8	63	2.73	1	0	0	1	2	0.00	NA	NA	NA	NA	NA	NA	53	3	0	9	65	2.68
Gurunsi	5	0	0	0	5	0.00	1	0	0	0	1	0.00	20	3	0	1	24	6.52	26	3	0	1	30	5.17
Konkomba	NA	NA	NA	NA	NA	NA	NA	NA	NA	NA	NA	NA	19	1	0	0	20	2.50	19	1	0	0	20	2.50
Mo	NA	NA	NA	NA	NA	NA	NA	NA	NA	NA	NA	NA	18	1	2	0	21	11.90	18	1	2	0	21	11.90
Other	9	0	0	1	9	0.00	2	0	0	0	2	0.00	59	1	0	3	63	0.83	70	2	0	4	74	1.39
Total (N)	182	5	0	16	203	1.34	36	4	0	3	43	5.00	132	8	2	5	147	4.23	351	18	2	24	393	2.96

Note: Genotypes for *GYPB* DEL1 and *GYPB* DEL2 in individuals from the 6 most-represented ethnic groups (N≥20) in the study, and by collection site.

A total of 393 individuals were genotyped for *GYPB* DEL1, DEL2, and *GYP Dantu* (DUP4) variants by PCR-RFLP with 325 (76%) of the participants representing 6 ethnic groups. The *GYPB* DEL1 and DEL2 allele frequencies are shown as percentages (%) within ethnic populations and across Ghana with N as the total number of participants and NA as no samples represented.

Genotypes were coded II – 'normal' with respect to the assay; DI – heterozygote for variant assayed; DD – homozygous for the variant assayed and XX for failed samples. Failed samples are included as these may be due to technical reasons or other *GYP* variants not detected by the assays used here. *GYP Dantu* was not found at any study site (Supplementary Table 2).

### Sequence analysis of *GYPB* DEL1 and DEL2 PCR products

For Sanger sequencing, PCR amplicons were separated on the agarose gels and extracted from the gel using the Qiagen PCR gel-extraction kit (Qiagen QIAquick Gel Extraction) as described by the manufacturer. The concentration of the DNA recovered was determined by Quant-i Picogreen assay (Invitrogen, UK). Samples were prepared following instructions described by the sequencing company and sent for Sanger sequencing by Eurofins Genomics (Ebersberg, Germany [https://www.eurofinsgenomics.eu/en/custom-dna-sequencing/eurofins-services]). The sequence data were inspected and curated using Chromas (https://technelysium.com.au/wp/chromas/) to generate FASTA files for the different sequencing reactions. These data were aligned using the multiple-sequence-alignment tool Clustal Omega (https://www.ebi.ac.uk/Tools/msa/clustalo/), after which pile-ups were manually curated and residues annotated according to the consensus sequence with respect to the PCR amplicon primers. Paralogous sequence differences between the three genes SDUs were used to confirm the PCR products for both DEL1 and DEL2 and also to identify the putative breakpoint regions for *GYPB* DEL1 and DEL2.

## Results

### Development of novel *GYPB* DEL1 and DEL2 PCR-RFLP assays

**Figure 3.**
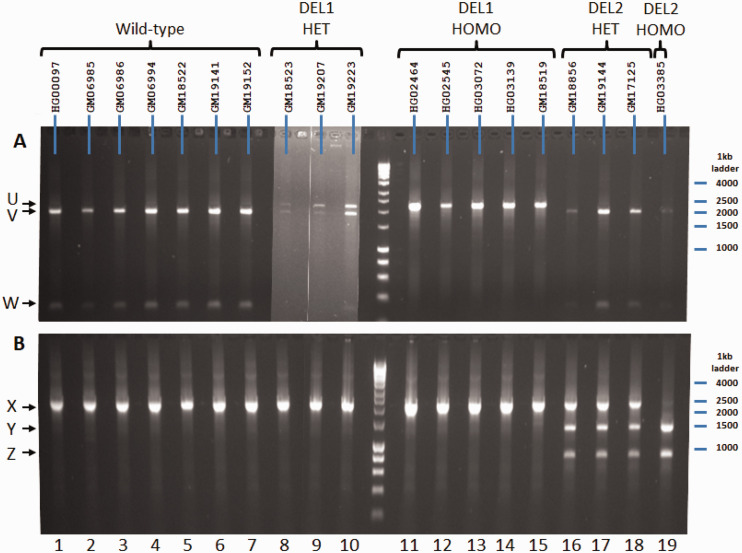
*GYPB* DEL1 (a) and DEL2 (b) assays on cell lines with known *GYP* States. PCR using the *GYPB* DEL1 (a) or DEL2 (b) primers was carried out on the samples followed by AciI or BsrBI restriction enzyme digestion, respectively. (a) DEL1 assay; The first seven wells (1–7) contain wild-type cell lines giving bands at 1.9 kb and 0.3 kb (V and W, respectively), five wells (11–15) contain homozygous cell-lines which give a single uncut band at 2.2 kb (U), while three cell-lines heterozygous for DEL1 (lanes 8–10) show two upper bands and one small band (2.3 kb [U], 1.9 kb [V], and 0.3 kb [W]). Lanes 16–19 are *GYPB* DEL2 positive cell lines that are all cut by the AciI enzyme (1.9 kb [V] and 0.3 kb [W]) indicating “normal” or non-DEL1. (b) *GYPB* DEL2 assay; The first seven wells (1–7) contain wild-type cell lines giving a single uncut band at 2.1 kb (X); lane (19) contains a *GYPB* DEL2 homozygous cell line giving two bands (1.3 kb [Y] and 0.8 kb [Z]); three wells (16–18) contain heterozygous cell-lines which give three bands (2.1 kb [X], 1.3 kb [Y], and 0.8 kb [Z]). Lanes 8–15 are DEL1 positive cell lines that are not cut by BsrBI indicating “normal” or non-DEL2.

**Figure 4.**
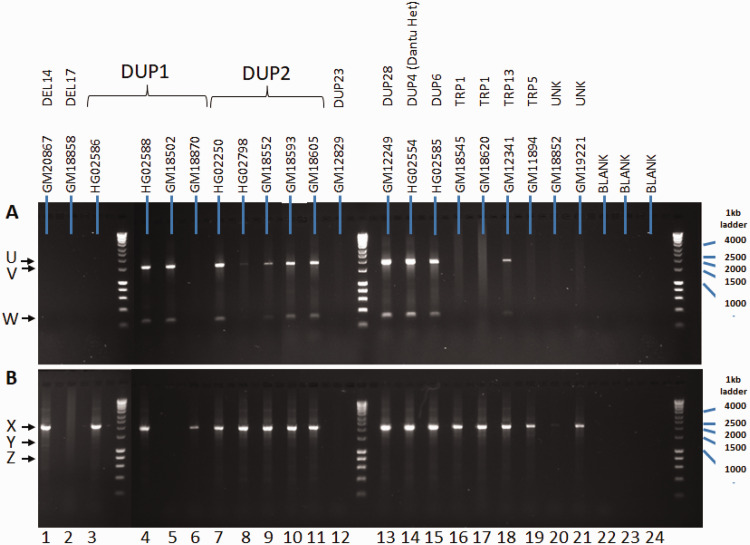
*GYPB* DEL1 (a) and DEL2 (b) assays on cell lines with known *GYP* states other than *GYPB* DEL1 and DEL2. PCR using the *GYPB* DEL1 (a) or DEL2 (b) primers was carried out on the samples followed by AciI or BsrBI restriction enzyme digestion, respectively. Lanes 22–24 are negative control wells. (a) DEL1 assay; 1.9 kb (V) and 0.8 kb (W) identify the bands expected for a non-DEL1 sample, and 2.2 kb (U) identifies the presence of *GYPB* DEL1. (b) *GYPB* DEL2 assay; 2.1 kb (X) band identifies a non-DEL2 sample, and the 1.3 kb (Y) plus 0.8 kb (Z) bands identify the presence of *GYPB* DEL2. See also Figure 4. Sample designations identified from Leffler et al.^9^

We have successfully designed a PCR-RFLP assay for *GYPB* DEL1 using an AciI restriction enzyme digest to differentiate *GYPB* DEL1 from the reference (“normal”/wild type) or non-DEL1 forms. A schematic representation of the strategy for the restriction digest is presented in Figure 2(a) and 2(b) and Supplementary Figure 2. The DEL2 deletion assay was designed in a similar way as the DEL1 assay using the BsrBI restriction enzyme (Figure 2(c) and (d) and Supplementary Figure 3). Several cell lines identified as DEL1 or DEL2 homozygous or heterozygous,^9^ were used to test both assays and the expected banding patterns were observed (Figure 3). The non-DEL1 homozygous reference samples gave two visible PCR amplicons on agarose gel-electrophoresis after AciI digest (1.9 kb and 0.3 kb), while the DEL1 deletion homozygote samples were not cut and gave a single visible 2.2 kb amplicon. Samples that are heterozygous for DEL1 gave a combination of all three bands (0.3 kb, 1.9 kb, and 2.2 kb) (Figures 2(a), 2(b), 3(a) and Table 4). Four other smaller products (all less than 50 bp) are produced in this AciI digestion but are present in both the reference and alternate PCR amplicons (Table 4) and are generally not visible or resolvable. Similarly, the *GYPB* DEL2 assay showed clear discrimination of genotypes using the BsrBI restriction enzyme (Figures 2(c), 2(d) and 3(b)). Reference non-DEL2 samples gave a single uncut band at 2.1 kb, while the alternate DEL2 variant gave visible bands at 1.3 kb and 0.8 kb showing that it was cut by this enzyme. Similar to the DEL1 assay, the DEL2 heterozygous samples showed three distinct bands. It is worth noting that samples that show negative result for any of the deletions should be classified as non-DEL1 or non-DEL2, respectively, since the assays only detect variants positive as homozygous or heterozygous. Also, the PCR-RFLP assays that we developed were used on non-DEL1 and non-DEL2 cell-lines that carried other *GYP* structural variants to confirm specificity of reactions and assays (Figure 4). Across the other cell lines tested, only non-DEL1 and non-DEL2 banding patterns were observed after restriction digest and gel electrophoresis, thus confirming the specificity of the assays.

### Sanger sequencing of PCR amplicons for *GYPB* DEL1 and DEL2

Several HapMap/1000G cell-lines (“normal,” DEL1 homozygote and DEL2 homozygote) as well as Ghanaian samples which comprised of three “normal” (non-DEL1, non-DEL2, and *Dantu* negative), three *GYPB* DEL1 homozygotes, one DEL2 homozygote, and one DEL1-DEL2 heterozygote were selected for Sanger sequencing of the PCR-RFLP amplicons. Pile-up sequences were aligned with the genome reference sequences (GRCh38) (Supplementary Files 2 to 5) showing that the “normal” amplicons were derived from the expected *GYP* regions (*GYPE*-*GYPB* for the DEL1 assay and *GYPB-GYPA* for the DEL2 assay [Supplementary Figure 1]). Amplicons from samples identified as homozygous for either DEL1 or DEL2 showed hybrid sequences (*GYPE*-*GYPB*/*GYPB-GYPA*).

**Figure 5.**
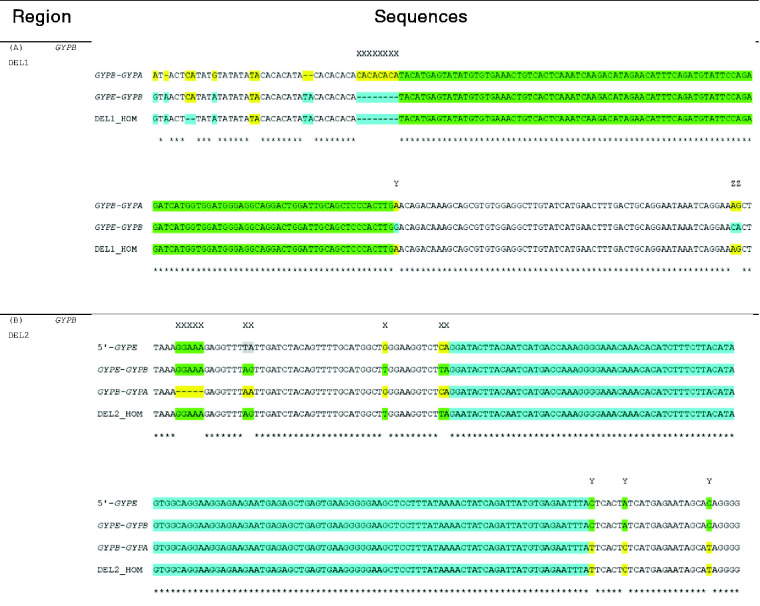
Breakpoint sequences identified for *GYPB* DEL1 and DEL2 from Sanger sequencing. (a) The *GYPB* DEL1 highlighted regions correspond to 4:143914016-143914126 (*GYPE-GYPB*) and 4:144024254-144024364 (*GYPB-GYPA*) in GRCh38; XXXXXXXX; identifies the 5ʹ boundary for the *GYPB* DEL1 breakpoint, Y identifies the 3ʹ end, and ZZ identifies 2 further distinguishing bases 62 bases downstream. (b) The *GYPB* DEL2 highlighted regions correspond to 4:143870925-143871054 (5ʹ-*GYPE*), 4:143991718-143991848 (*GYPE-GYPB*) and 4:144094974-144095103 (*GYPB-GYPA*) in GRCh38. Bases marked with X's identify the group of distinguishing bases at the 5ʹ end of the *GYPB* DEL2 breakpoint, while the Y's identify the 3ʹ end of the *GYPB* DEL2 breakpoint. Supplementary Files 1–5 provide further detail and Sanger Sequencing pile-ups. The *GYPE* sequence was included to confirm that the amplicons were not amplifying this gene region.

For DEL1, the sequence changed from *GYPE*-*GYPB* to the *GYPB-GYPA* sequence between bases 1672 and 1783 in the amplicon (corresponding to 4:143914016–143914126 [*GYPE*-*GYPB*] and 4:144024254–144024364 [*GYPB-GYPA*] in GRCh38). The 5ʹ boundary was identified by a tandem repeat motif made up of CA and AT repeats (XXXXXXXX in Figure 5(a)), while the 3ʹ end was marked by a single paralogous base difference (A/G, marked Y in Figure 5(a)) between the reference sequences. A further 62 bases upstream from the 3ʹ boundary, there is also a 2-base paralogous difference between the reference sequences (ZZ in Figure 5(a)). The region bounded by these distinguishing motifs identifies a 111 base sequence within which the putative breakpoint occurs and is ∼8.5 kb from the *GYPE* ATG start site and ∼4.9 kb from the *GYPB* ATG start site, and deletes 110 kb to form DEL1 (Supplementary Files 4 and 6). For DEL2, the sequence changed from *GYPE*-*GYPB* to the *GYPB-GYPA* sequence between bases 1043 and 1172 in the amplicon (corresponding to 4:143991718–143991848 [*GYPE*-*GYPB*] and 4:144094974–144095103 [*GYPB-GYPA*] in GRCh38). The 5ʹ boundary is identified by 4 different paralogous motifs, all within 50 bases of each other (5 base INDEL, 2 × 2 base difference, and 1 single bases difference – marked with X's in Figure 5(b)). The 3ʹ end is marked by 3 separate single paralogous base differences between the reference sequences, all within 23 bases of each other (marked as Y in Figure 5(b)). This identifies a 129 base sequence within which the putative breakpoint occurs and is located ∼86 kb from the *GYPE* ATG start site and ∼76 kb from the *GYPB* ATG start site, and deletes 103 kb to form DEL2 (Supplementary File 5).

### Distribution of *GYPB* DEL1 and DEL2 genotypes in Ghana

**Figure 6.**
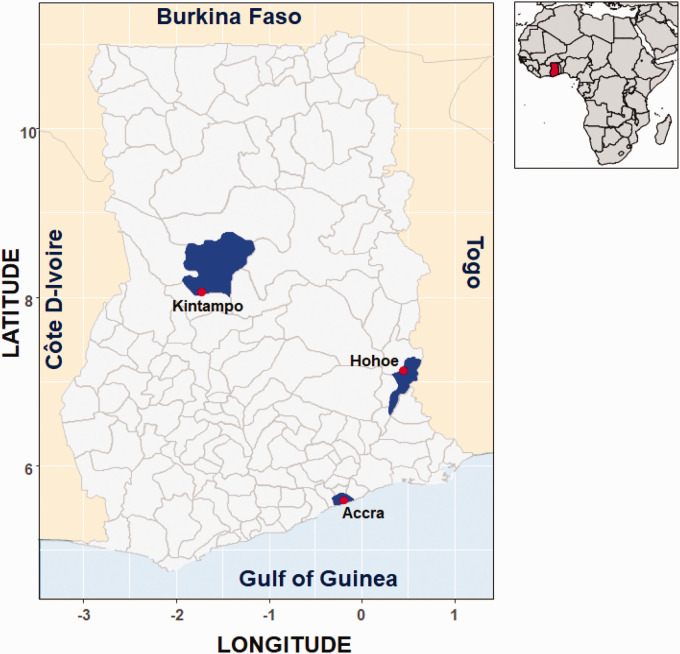
Location of sampling site in Ghana. Boarders for level 2 administrative districts are shown within Ghana with the sampling districts filled in blue. The major towns where sampling was conducted are shown and named. The offset Africa map shows the location of Ghana in red. The map was generated using R (https://www.r-project.org/) using a shape file downloaded from GADM (https://gadm.org/download_country_v3.html). Town GPS coordinates were identified from Google Maps (https://www.google.com/maps).

The assays developed were used to genotype DNA samples obtained from volunteers who had been enrolled into various ongoing studies in three areas of Ghana, namely Accra, Kintampo, and Hohoe. Individuals from Accra and Kintampo were all children between the ages of 1 to 15 years (mean 5.4 years from Accra [47:53% male:female ratio] and 3.25 years from Kintampo [41:59% male:female ratio]), while individuals from Hohoe were all adults (>18 years) with 39:61% male:female ratio (Table 5 and Figure 6). In total, 393 individuals were included for genotyping after curating for self-reported ethnicity to only include those of Ghanaian origin. There were 21 distinct self-reported ethnicities (Supplementary Table 1) of which 6 had N ≥ 20 individuals (No. chromosomes ≥ 40 providing a minimum detection of 2.5% allele frequency; Table 5).

The two new *GYPB* deletion assays as well as the assay for detecting the *Dantu* variant were used to measure the frequency of the DEL1, DEL2, and *Dantu* variants among the selected individuals at the three locations in Ghana (Table 6 and Supplementary Table 2). *GYPB* DEL1 was present at all three sites, with an overall *GYPB* DEL1 allele frequency of between 4.1% and 6.2%, while *GYPB* DEL2 was estimated at an overall allele frequency of between 1.3% and 5.0%. In Accra, the frequency of DEL1 was 4-fold higher than DEL2, while in Hohoe and Kintampo they were similar (4.1% vs. 5.0%, and 5.7% vs. 4.2%, respectively). As expected, we did not identify any individuals carrying the *Dantu* variant^9^ (Supplementary Table 2). The number of individuals from different ethnic backgrounds influenced the overall allele frequencies which skewed the overall estimates. The data were therefore analyzed using the main ethnic groups represented in the dataset which were at least 20 individuals for any given group.

Of the 393 samples across the 3 study sites in Ghana, 325 came from 6 ethnic groups (Akan, Ewe, Ga, Konkomba, Mo, and Dagarti; Tables 1 and 2). *GYPB* DEL1 varied between 1.8% and 9.0% overall, while *GYPB* DEL2 varied between 0.7% and 11.9% overall. When analyzed by the study site, the DEL1/2 frequency estimates became more unreliable due to small sample sizes but where sites-ethnic groups had N ≥ 20, the estimates ranged from 2.17% to 7.4% (DEL1) and 0.7% to 11.9% (DEL2) (Table 6). In total, there were 21 ethnic groups represented of which 11/21 possess DEL1 (of the other 10 groups where DEL1 was not detected, all had 4 or fewer individuals). For DEL2, 7/21 groups showed the presence of DEL2 (but not necessarily the same groups as DEL1, Supplementary Table 2). *GYPB* DEL2 was not detected in 14/21 groups (of which 10 had 4 or fewer individuals and 4 groups between 8 and 19 individuals).

## Discussion

Glycophorins on the surface of erythrocyte are used by malaria parasites to mediate invasion,^11^ as such, variants of the gene may protect against malaria parasite infection through mechanisms such as slowing parasite growth or reducing chances of developing severe malaria.^8,9^ To better assess the effects of these structural variants on resistance to malaria, there is a need for population surveys in malaria-endemic regions across sSA to generate prevalence data and identify phenotypes for conducting functional studies. However, surveys have been limited by the lack of reliable high-throughput assays for the identification of such phenotypes. The challenge of designing high-throughput PCR-based screening assays is due to the high sequence homology (>96%) between the *GYPE*, *GYPB,* and *GYPA* genes.^29,30^ In this study, we overcame this challenge and successfully designed two high throughput PCR-RFLP protocols that detect the presence of the two most common *GYPB* deletion variants in West African populations, *GYPB* DEL1 and DEL2. The assays were confirmed by Sanger sequencing and analyzing the PCR products of the assays. Furthermore, these assays can be performed in a 96-well plate format, followed by agarose-gel electrophoresis, making it possible to run over 90 samples in a single experiment. The assays were validated in the field by comparing their performance with an existing assay for the detection of the *GYP* *Dantu* variant (DUP4) in screening 393 individuals from three study sites in Ghana. The specificity and sensitivity of the assays in the field demonstrate their applicability as less time-consuming and less expensive options to long-read sequencing for conducting large-scale studies on the population distribution of these variants.

Details for the putative location of the breakpoints for *GYPB* DEL1 and DEL2 originally came from a previous study that used whole-genome sequencing from the 1000 G samples, plus additional African samples.^9^ This initial information was used to align the reference sequences for *GYPA, GYPB,* and *GYPE* across the putative breakpoints and identify the paralogous differences. Due to the high homology between the three *GYP* regions, identifying primers that will specifically amplify each region was challenging. It was however easy to design a single primer that could anchor the three genes and their surrounding regions. These features were used to overcome the challenge of developing an assay to differentiate between the three genes and their surrounding regions. For *GYPB* DEL1, a forward primer was designed to bind to the unique sequences near the *GYPE* transcription start site, while the counterpart (reverse primer) was common to the three regions. In the case of DEL2, designing a specific primer was more challenging because the breakpoint was not close to any entirely unique region. To overcome this, the DEL2 specific primer was placed at a location on the gene cluster close to the *GYPB* DEL2 breakpoint with sufficient sequence variation to allow the PCR conditions to discriminate between the sequences. In view of the fact that both the resulting reference and deletion amplicons for each assay would be of the same length, restriction enzymes were used to distinguish between them. The restriction enzymes AciI for the digestion of *GYPB* DEL1 PCR product and BsrBI for *GYPB* DEL2 PCR products were identified and selected. Other restriction enzymes may work well but have not been explored or tested in this study. One problem with using restriction enzymes is the possibility that the recognition sites themselves may contain population variation that could complicate the interpretation of the assays; however, from current information in genome-browsers and variation databases (dbSNP153), this variation appears to be uncommon for the restriction sites used here.

The two assays were used to analyze DNA from HapMap and 1000 G cell-lines that had known *GYPB* types identified in the Leffler *et al.* study.^9^ This allowed further optimization of the PCR conditions and also the use of Sanger sequencing to validate the amplicons produced by the PCRs. When the homozygous *GYPB* DEL1 or DEL2 samples were amplified, the sequences obtained could be seen to change from one reference sequence to another and the paralogous sequence differences were used to identify the region where the switch occurred from *GYPA to GYPE*. Further sequence data would be required to identify whether these breakpoint boundaries are the same for all *GYPB* DEL1 and DEL2 chromosomes and begin to understand whether the flanking sequences were important for the mismatches during chromosome replication. In both *GYPB* DEL1 and DEL2 deletions, the equivalent of a whole SDU was removed amounting to ∼100 kb each.

The performance of the two assay systems we have developed was evaluated by screening nearly 400 individuals from three different sites in Central to Southern Ghana. Considering the ethnic diversity at each study site and the ethnic group sample numbers, the overall allele frequency of *GYPB* DEL1 varied between 4.1% and 6.2%, while that of *GYPB* DEL2 varied between 1.3% and 5.0%. Work done by Gassner *et al*.^31^ reported that allele frequencies of the other three distinct deletions within African ethnicities varied greatly, to the extent that among the Congolese Mbuti Pygmy populations, cumulative allele frequencies were as high as 23.3%. The frequencies reported in our current study are similar to frequencies reported in other West African populations.^9^ In this study, analysis of the main ethnic groups with at least 20 individuals, showed the allele frequencies of DEL1 and DEL2 varied between ∼1% and ∼12%, with *GYPB* DEL2 mostly lower than DEL1. It is worth noting that the frequency estimation within ethnic groups where the sample sizes are below 100 (*n* = 200 chromosomes) will require confirmation by screening larger sample sizes to have a high power of study and confidence. In general, larger sample sizes would be required for DEL2 surveys as the allele frequency was less than that of *GYPB* DEL1. In all the ethnic groups with more than 20 individuals, we detected *GYPB* DEL1 and DEL2; however, non-DEL1 and non-DEL2 individuals could only be identified by increasing the number of study participants across all the ethnic groups. These two new assays will thus allow surveys on a larger scale to determine the distribution of these two main variants in other West African populations and also identify phenotypes for functional assays to investigate the effects of *GYPB* DEL1 and DEL2 on the susceptibility of erythrocytes to being invaded by *P. falciparum* and the resulting impact on disease pathogenesis.

The ability to identify these genotypes of interest from large populations accurately and rapidly has been an important goal in high-throughput genetic screening assay development.^32,33^ Therefore, the current DEL1 and DEL2 assays offer an opportunity for rapid screening of populations for these *GYPB* polymorphisms that are common especially in West Africa. Furthermore, for any malaria-related studies, it is important to collect alongside the *GYP* variants, other key genetic information such as sickle (rs334) which is present throughout Africa,^34^ HbC (rs33930165; present in Ghana and other West African Countries),^35^ and G6PD,^36^ as these may act as confounders in studies examining associations with susceptibility to malaria or effects on malaria parasite invasion and growth.

Developing less expensive high throughput assays targeting the less common *GYP* variants will provide a better understanding of the distribution and functional effects of these variants on susceptibility and pathogenesis of malaria and other disease causing pathogens that also use *GYPB* as a receptor. Such understanding may prove useful in guiding the design of vaccines or other therapeutic interventions targeting the pathogen interactions with these GYP proteins on the erythrocyte surface. These assays are also important for identifying individuals with the various genotypes of *GYPB* (homozygous, heterozygous and wild type), which is necessary for investigating the functional significance of these gene variations.

## Supplemental Material

sj-pdf-1-ebm-10.1177_1535370220968545 - Supplemental material for High-throughput genotyping assays for identification of glycophorin B deletion variants in population studies Click here for additional data file. Supplemental material, sj-pdf-1-ebm-10.1177_1535370220968545 for High-throughput genotyping assays for identification of glycophorin B deletion variants in population studies by Dominic SY Amuzu, Kirk A Rockett, Ellen M Leffler, Felix Ansah, Nicholas Amoako, Collins M Morang’a, Christina Hubbart, Kate Rowlands, Anna E Jeffreys, Lucas N Amenga-Etego, Dominic P Kwiatkowski and Gordon A Awandare in Experimental Biology and Medicine
