# Supplementary material for: High-throughput genotyping assays for identification of glycophorin B deletion variants in population studies
Source: Exp Biol Med (Maywood). 2020 Dec 16;246(8):916–28. doi: 10.1177/1535370220968545 (PMC8022085; doi:10.1177/1535370220968545)
Supplement: sj-pdf-1-ebm-10.1177_1535370220968545 - Supplemental material for High-throughput genotyping assays for identification of glycophorin B deletion variants in population studies [file sj-pdf-1-ebm-10.1177_1535370220968545.pdf]

SS1_Amuzu_et_al_Supplementary_File_1_Tables_and_Figures.pdf

SS2_Amuzu_et_al_Supplementary_File_2_DEL1_Pileup_GRCh37.pdf

SS3_Amuzu_et_al_Supplementary_File_3_DEL2_Pileup_GRCh37.pdf

SS4_Amuzu_et_al_Supplementary_File_4_DEL1_Sanger_Seq_GRCh37.pdf

SS5_Amuzu_et_al_Supplementary_File_5_DEL2_Sanger_Seq_GRCh37.pdf

SS6_Amuzu_et_al_Supplementary_File_6_GYPB Boundaries_GRCh37.pdf

Amuzu et al., Supplementary File 1 - Tables and Figures

High-throughput genotyping assays for identification of glycophorin B deletion variants in population studies

Supplementary Table 1: Ethnic group and number of individuals with a curated group identifier

Ethnicity	Number	Curated Ethnicity
Akan	98	Akan
Ewe	85	Ewe
Ga	65	Ga
Gurunsi	30	Gurunsi
Mo	21	Mo
Kokomba	20	Kokomba
Dagarti	19	Other
Gonja	13	Other
Dagomba	11	Other
Fulani	8	Other
Hausa	4	Other
Wala	4	Other
Basari	2	Other
Bimoba	2	Other
Chokosi	2	Other
Krobo	2	Other
Mamprusi	2	Other
Zambrama	2	Other
Banda	1	Other
Guan	1	Other
Nanumba	1	Other
**Total (N)**	**393**	

Self-reported ethnic group and number of individuals with a curated group identifier. The latter was used to create aggregate numbers for GYPB DEL1 and DEL2 (see **Supplementary Table 2**).

Supplementary Table 2: Distribution of genotypes for DEL1, DEL2 and Dantu across ethnic groups

Ethnic Group	Total	DEL1				DEL2				DANTU			
	(N)	II	DI	DD	XX	II	DI	DD	XX	II	DI	DD	XX
Akan	98	80	11	0	7	89	4	0	5	97	0	0	1
Banda	1	1	0	0	0	0	1	0	0	1	0	0	0
Basari	2	2	0	0	0	2	0	0	0	2	0	0	0
Bimoba	2	2	0	0	0	2	0	0	0	2	0	0	0
Chokosi	2	2	0	0	0	2	0	0	0	2	0	0	0
Dagarti	19	14	2	1	2	17	0	0	2	19	0	0	0
Dagomba	11	8	3	0	0	11	0	0	0	11	0	0	0
Ewe	85	66	7	0	12	76	4	0	5	82	0	0	3
Fulani	8	6	2	0	0	8	0	0	0	8	0	0	0
Ga	65	53	5	1	6	53	3	0	9	64	0	0	1
Gonja	13	9	1	0	3	13	0	0	0	13	0	0	0
Guan	1	0	0	0	1	1	0	0	0	1	0	0	0
Gurunsi	30	27	1	0	2	26	3	0	1	30	0	0	0
Hausa	4	3	0	0	1	2	0	0	2	4	0	0	0
Kokomba	20	17	1	0	2	19	1	0	0	20	0	0	0
Krobo	2	2	0	0	0	2	0	0	0	2	0	0	0
Mamprusi	2	2	0	0	0	2	0	0	0	2	0	0	0
Mo	21	16	2	0	3	18	1	2	0	21	0	0	0
Nanumba	1	1	0	0	0	1	0	0	0	1	0	0	0
Wala	4	4	0	0	0	4	0	0	0	4	0	0	0
Zambrama	2	1	0	1	0	2	0	0	0	2	0	0	0
**Total (N)**	**393**	**316**	**35**	**3**	**39**	**350**	**17**	**2**	**24**	**388**	**0**	**0**	**5**

Groups highlighted have ≤20 individuals overall.

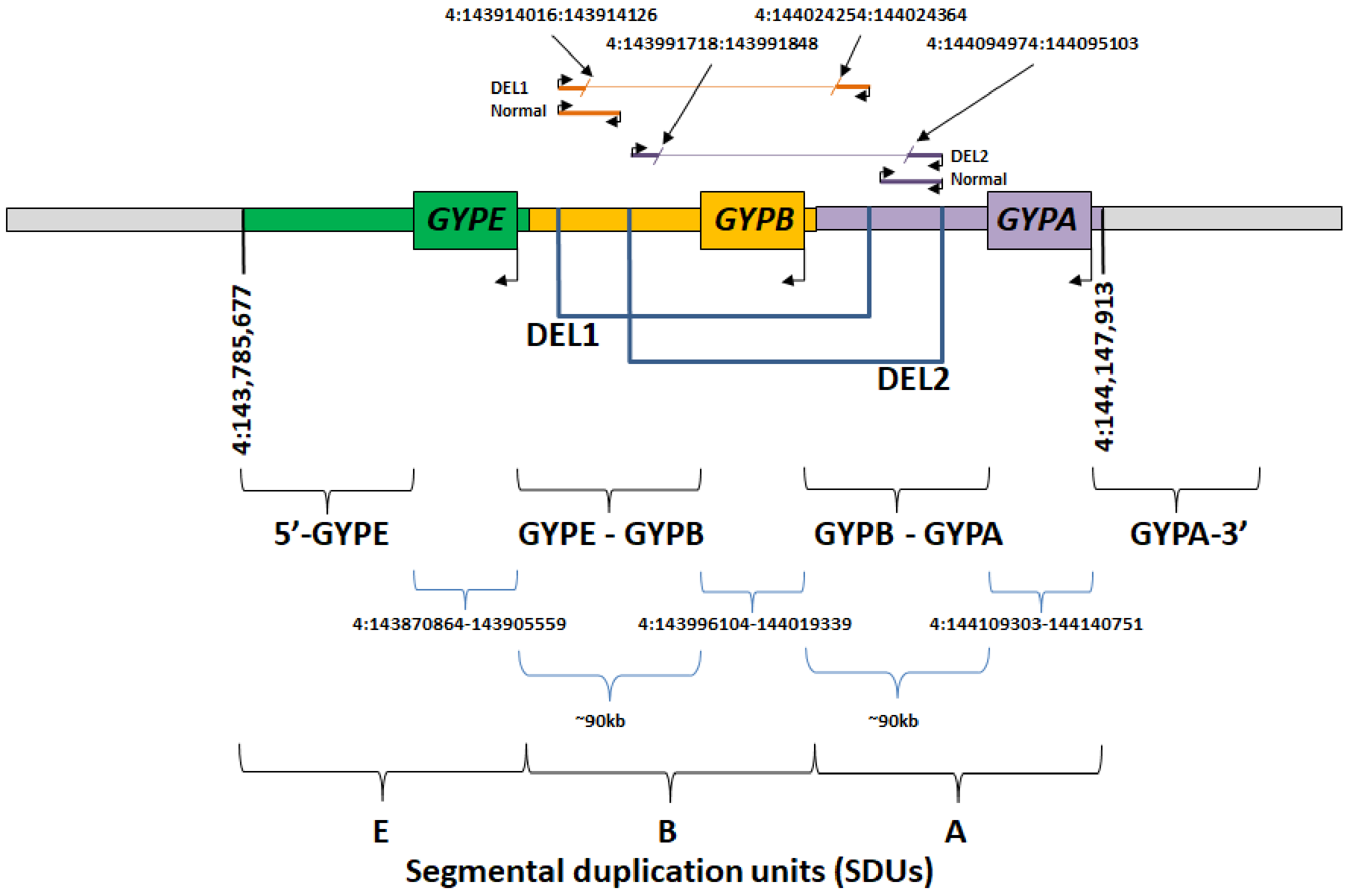

Supplementary Figure 1: Schematic diagram of the Human reference GYP gene region on chromosome 4.
The three GYP segmental duplications units (SDUs) are indicated by the different colours. The GYP gene-region boundary-locations are with respect to GRCh37 as indicated. The approximate locations of the DEL1 and DEL2 deletions are shown. The amplicons produced by the PCRs are shown above the gene map along with the putative breakpoint locations. The DEL1 specific PCR primer is located at the GYPE end. The DEL1 common primer maps to the GYPE-GYPB region and the GYPB-GYPA region giving a 'normal' amplicon in the GYPE-GYPB region while the DEL1 product is a hybrid of GYP-E-GYPB and GYPB-GYPA. The DEL2 specific PCR primer is located at the GYPA end. The DEL2 common primer maps to the GYPE-GYPB region and the GYPB-GYPA region giving a 'normal' amplicon in the GYPB-GYPA region while the DEL2 product is a hybrid of GYPE-GYPB and GYPB-GYPA.
Gene region names used in the main text are shown. After Leffler et al. (Leffler EM, Band G, Busby GB, Kivinen K, Le QS, Clarke GM, et al. Resistance to malaria through structural variation of red blood cell invasion receptors. Science. 2017;356(6343): eaam6393).

Amuzu *et al.*, Supplementary File 2: DEL1 pile-up.

Sequences of GYPE, B, A around the DEL1 breakpoint in FASTA format.
Sequences were downloaded from Ensembl and are with respect to GRCH37 (http://grch37.ensembl.org/index.html)

```
>DEL1:GYPE-GYPB:chromosome:GRCh37:4:144832652:144836671:1:GRCh38:4:143911499-143915518
TTATAATGTTAAGCTACCTGAAGCATGCCTCCCCTGATTCTGTGGCATTCTTTGGAAGAGATCATGGTTGTATTCATAATATCTGTAGTCTTCATGTGCACGATCTCCAAGTGGCCGCTTTCTCTGACCATCAACAAAATTGTCTGAATAATAATATCGGGAA
CCAGAGTCTCCATTTTCAACAGCAAAACTAACTTCCGTTACAAATGACTGAGAAGAACCATACTCTCTAGGGACATCTGCTAGACTTCTGGCAAGATAAGAAGGTGCAGATAATCTGTTGCTTTCTCTTCTCATTATTTTATGAGGTGTTCTTTGAATTGGAG
TTCTAAGGAAACTCTGGTTTCTTGAAACTACTCCATCTCCAGAAGTTGAAAAGGCATTAGGGCTTGAATCTGGAAGACAGATATCAGTTCGTCCTGGAATTTTTGGACCATGCTGGATGAATGAAGGCATAAGATTCCGAAGCAGAGGCGACGTCTCCTTCTT
CACGTACTTCCCCTGCACCTCGGCCGGCTTGGACACTTCGTTTTTGGTTTTCTGTCGGGACAGCATCCTTCTCGACCAGGCCCGAGGCCGCGCTGAACTGCCTCGCTAGGGCTCCGCGCCGGGCGCCGGCCGTCTCCGCCGCCACCTCCGCCGCCACCTCCGC
CGGCGCCGCCGCCTCCTTCCCTCCGGAGCCGCCGCCTCCGCCGCTGCCTGTCCGGAGCCTGGGGTCGCCCGCAGGGACTGCCGCATGTTCAGGGCGCTAAACGCGCCGGCCGCCGCTCAGTCGCTGGTCACTTCCTTCCCGGAAGTCGGCCCGCTCTGCGACG
CTGCTCGGGGACCCCTTGAGGAAAGCCCAGCGACGCCGGGCCAGCGCATTCTTATTTTATGTATGAGCAAACTATGGAATGGAAAAGAGATTATGAGTTATTACTTGATTTGATTTGCCCTAAGTGTTTTTTAAACGTATGGATTTAGCGCCAACATTTAAAA
AGAAGGTATATTTTAATTAAAAATCAGGAATTGATTTAATTTAAAATTTGGCTCCTCTTACAGGAAGATCTGGCAAGAGTTAGCGTGCAGTTCTACATTGTGACAATTCATGAGAGCTAAGGACTGGCTGTCTCTTGGGACAAATAGGCTTTCTTGAGTCTGG
TAAGTGCTCCACTTGATGCTTGCTTCCATTTCTTAGATTAGGTTCTACTGTGGTAAGGCCTCAAAATCTCAGTGTCTTGCAGCAACACTGGTTCATTTCCTGCTCACATTACATTTGGTCTATGACGGTGACAAGTTCCAGGATCCAGACTGAAGGAGCCACC
CCCATTTGGAATATGCTGTTCTTGTTGCAAACTGGGGGAAAAAGGCCAGATAAGGAATGGCTTTTAATGCTTCCTCTCAGACTGTGTTATGGTTTTTTTCTCACATTCTATTAGCCAAAGTAAATCAGTGGCCAAGCTGACCTCAATGGGACAAGTAAATATA
ATTCTTCCAAATGGAAGTCCTGCAAGTCATAGGTCAATGGTGAGGATATATAAAACACTTACAGGAAGGGCCCTAATAATTGGGAGCAAAAACACAATCCATCGCATTCATTTATACTCTTTCTAAGCTTTTGGCATTTGGGTTTGGAAATCTAATTTATTTA
GGAGTCTTCTTCAACACCTTCTAGTGATGAGATTTCTTTAACCCAGTTTTCAGTTTCCTTTCTTTAAAATTATAATGAGATGTGATGTGCGTTGAAGAAACCCCAGAAGTGAGAAACTCTCATCACTACAATGGTGATGTAGACAGTGGAAAACTGTTCCCAG
TTGCCTCTAAGTCCATCGTGCTATACTGCTGGAATCAGGTATCATACATAATCTTTCTGCTGAGAATTGATAGGTCCAGCTTGTTCTACATCCAAATAATAATTCAACCAATGATTTGTGAGCACCTACCGGGCACTGTGCCAAGTGTGGAAGACACAACCTT
CAGTAACTCAAGTTTCCCTTCAGGAACTGACAGCTTAATTTGGAAACAAATAAATTAAGAAGGCAATTGCATTATGGAGTGTCAAGTGGATGATAGTAGTAAGCAAAGGGTGTTATAAGGGTACATAAGAGGGGTACCTAACTCAGGGTTGAGGTTCTGGAAA
GGTTTTCCAGAGCAAGCAATGAGACCTAAAGGATGAGTAGACAAGTCACGGGAGAAGTAAGAACATTTTAAGCAGAGAAAACAGCATGAGCAATGGTCTGATATCAAGAAAGGCAGAGATAGTGCAAGAATACACAATGTAGTAGTTTTCAAAACTGTTTGAA
ATTTTAATCTCACATTTGTTCTTTTCAATGTCTAGGATATCAATTCATTCTGCGATTTACCTTTGTAAAGATGAATTTAGCCCTGGTTCTCTATATGAATACTAGAGGAAGTATATTGATAAAAGAAAAGGAACAAATATGTAGTCATTTCTAAGATTTATTT
TTATTAAAATGTAACACTCACAAACTGTAAGTGTGTAACTCATATATATATATACACACATATACACACACATACATGAGTATATGTGTGAAACTGTCACTCAAATCAAGACATAGAACATTTCAGATGTATTCCAGAGATCATGGTGGATGGGAGGCAGGAC
TGGATTGCAGCTCCCACTTGGACAGACAAAGCAGCGTGTGGAGGCTTGTATCATGAACTTTGACTGCAGGAATAAATCAGGAACACTGAGAGAACCCACAGACCCTCAGAAAGAAGCGGATTGCTCCTGCAGGTCTCAGGAGACACCCCAAATGCTGTGGGAG
CCCAAACTGCAAACTGTGGAAGTGGGAAAGGGGAATAGTCAGCTCCTGAACACACATCCTCACTGGGGAACCTAAAGGTCTAGATCACAGGAGAAGATTTTGACCTTACTTGGAGCTGAGTCAATTTAGAGAGCCAAGTGACATACACTGCTAGAGAAAGCAG
GGCGTAAGGCCCTGTGGGCTCACTGGGTTCCCTAGCCATCCATTTCTGCCTTGCCTCACGGGGGTCCTTGAGGAGGGCTACCAGAGGCACTGGGAAATGGTCACAAAGAGAAGGAAACTTCCAGCTGAACTTTAAAACAATTTGAACAGATTGAGAAGTCTCC
TAGCCAGAACTTGGGGGAGGGCATGAATCCAGTGTGCAGACTCTACAGGTGGGGAAGTACCAAAGCCCTACTTGCTTTTGCAGCTGGGAGGCTGGTAGCCTGGGGCAAATTCTCAGCCCTGCTCACCCACTGCCTGGAAACAGACTCGGTGCTGTTGCAGGGG
CTATGGTGGGAGTGAGACCAGCCCTTTGGGTTATGTGGGAGCTGGGTGAGGCCTGTGACTTCAACTTCCCTGACAACCTGCATGGGATAAAAATCAGGGATAAAAATCACTACAGCTCTGCTCTCAGGAAGCCACATCCCTGGGAAAAGTGGGAGAGTACTAC
ATCAAGGGAACACCCTGTGGGACAAAAGAATCTGAAGAACAGCCTTGAGCCCTAGACTTTCCCTCTGACAGAGCCTACCCAAAGGAGAAGGAACCAGAAAGCCAACTGTGGTAATATGACAAAACAAGGTTTTTAACACCCCCCAAAATCTCACTAGCTCA
CCAGCAATGGATCCAAATGAAGAAGAAATCCCTGATTTACGTGGAAAAGAATTCAGAAGGTTAGTTATTAAGCTAATCAAGGAGGTACCAGAAAAGGCAAAGCCCAATTTAAGGAAGTGAAAAAAAATGATACAAGAAATGAGGGGAGAAAGTTTCAATTAAA
TAGATAGCATAAATAACAAACAATCAAAACTTCAGGAAAAAAATGGATGCGCTTATAGAAATGCAAAATGCTCTGGAAAGTCTCAGCAATAGATTCCAACAAGCAAAAGAAAGAACTTCAGAGCTCGAAGACGAGGTTTTCAAATTAACCCAAACCAACAAAG
ACAAAGAAAAAAGAATAAGAAAATACAAGGAAAGCCTGCAAGAAATCTGGGATTATGTTAAACAACCAAACTAAGAATTATCAGCCTTCTTGAGGAAGAAGAGAAATC

>DEL1:GYPB-GYPA:chromosome:GRCh37:4:144942890:144946909:1:GRCh38:4:144021737-144025756
CAGATTCAGCACCTCATACAGGGTGTGAGGAAGCAGAGCCATATAGTTCCCTCTAGTGGCGTATCTGCACAATGGAAGGAAAAAATGTGAGCCCCGTAAATGCAACAGAAAAAAATATACGTCAGATTACATTTTTCTCTTTCAGGCATTCCCTAATCTTCTT
TAAATTTTTCAAAAATATTTTTACTGAAATACACATTCAGAATTATCATAAAATTCTGCCTAAGAGCTATATCATGCAAATTATTAGACAAATCCACATCTTTTAAGAATTTTGACTCTGCATAAAATAAAAGAAGTACTTTTGAGGCATTTGATCAAACATA
AAAAACTTATCCAATTTTCCTGATACTACATGTCTTTAGGCTATTTTTTGAATTGCCTGAGTCATTTCTATGACGTAGTTAAGAAGGATGAAGTTCAATGTTATAATCTAGGAAAGGCAACTTGGATCGATCAACACAAACTTATACCTTGCTTGTTTCATGA
CAGAGAACCATTCATTTTTGTCAATTACTTACCTCTCAACTATTTCAGGCTTAGATTATTCCTGAAGATGGTTGCATGCTAGTCTGATCTGCCAGTGGAGAAGTGTGCTTTTCATGTTTTGCATGGAGGAGTGGAGAACAGGGGAGGGGCAGGAAAGTGGAGG
CACAGTGGTTTATACTACTATCCTCCTTGGCACCTCATCGCCTTAGAATTTTCCAGCCTCCTTTGCCAGAGTAGTCTAAGAACTTTCCCTGACCTTTGTTTGTTTATCATTTGAAATTTTTTTTAAATAAAAAAACAACTACAAGGTAAGATAAATGTAACGC
TCAAAATAACCCTGTTAGAGAGGCAGAACAGATATTAGCATTCTTATTTTAAATATGAGCAAACTATGGAATGGAAAAGAGACTATGAGTGTATTACTTGATTTGATTTGCCCTAAGTGTTTTTTAAACGTATGGATTTAGCTGCTAACATTTAAAAAGAAGG
TATATTTTAATTAAAAATCAGGAATTGATTTAATTAAAAATTTGGCTCCTGTTACCGGAAGATCTGGCAAGAGTTAGCATGCAGTTCTACATTGTGACAATCCATGAGAGCTAAGGACTGGCTGTCTCTTGGGACAAATAGGCTTTCTTGAGTCTGGTAAGTG
CTCCACCTGATGCTTGCTTCCATTTCTTAGATTAGGTTCTACTGTGGTAAGGCCTCAAAATCTCAGTGTCTTGCAGCAACACTGGTTCATTTCCTGCTCACATTACATTTGGTCTATGACGGTGACAAGTTCCAGGATCCAGACTGAAGGAGCCACCCCCATT
TGGAATATGCTGTTCTTGTTGCAAACTGGGGGAAAAAGGCCAAATAAGGAATGGCTTTTAATGCTTCCTCTCAGACATGGTATGTGTTATTTTTTTTTCTCATATTCTATTAGCCAAAGTAAATCAGTGGCCAAGCTGACCTCAACGGGACAAGTAAATATAA
TTCTTCCAAATGGAAGTCCTCCAAGTCATAGGTCAATGGTGAGGATATATAAAACACTTACAGGAAGGGCCCTAATAATTGGGAGCGAAAACACAATCCATCACATTCATTTATACTCTTTCTAAGCTTTTGGCATTTGGGTTTGGAAATCTAATTTATTTAG
GAGTCTCCTTCAACACCTTCTAGTGATGAGATTTCTTTAACCCAGTTTTCAGTTTCCTTTCTTTAAGATTATAATGAGATGTGATGTGCGTTGAAGAAACCCCAGAAGTGAGAAACTCTCATTACTACAATGGTGATGTAGACAGTGAAAAACTGTTCCCAGT
TGCCTCTAAGTCCATTGTGCTATACTGCTGGAATCAGGAATCATACATGATCTTTCTGCTGAGAATTGATAGGTCCAGCTTGTTCTACATCCAAATAATAATTCAACAAATGATTTGTGAGCACCTACCGGGCACTGTGCCAAGTGTGGAAGACACAACCTTC
AGTAACTCAAGTTTCCCTTCAGGAACTGACAGCTTAATTGGGAAACAAATAAAGTAAGAAGGCAATTGCATTATGGAGTGTCAAGTGGATGATAGTAGTAAGCAAAGGGTGTTATAAGAGTACATAAGAGGGGTACCTAACTCAGACTTGGGGTTCTGGAAAG
GTTTTCCAGAGCAAGCAAGACCTAAAGGATGAGTAGACAAGTCACGGGAGAAGTAAGAACATTTTAAGCAGAGAAAACAGCATGAGCAATGGTCTGATACCAAGAAAGAGAGAGATAGTGCAAGAATACACAATGTAGTAGTTTTCAAAACTGTTTGAAATTT
TAATCTCACATTGGTTCTTTTCAATGTCTAGTATATCAATTCATTCTGCCGTTTACCTTTGTAAAGATGAATTTAGCCCTGGTTCTCTATATGAATACTAGAGGAAGTATACTGATAAAAGAAAAGGAACAAATATGTAGTCATTTCTAAGATTTATTTTTAT
TAAAATGTAACACTCACAAACTAAGTGTATAACTCATATGTATATATACACACATACACACACACACACACATACATGAGTATATGTGTGAAACTGTCACTCAAATCAAGACATAGAACATTTCAGATGTATTCCAGAGATCATGGTGGATGGGAGGCAGGAC
TGGATTGCAGCTCCCACTTGAACAGACAAAGCAGCGTGTGGAGGCTTGTATCATGAACTTTGACTGCAGGAATAAATCAGGAAAGCTGAGAGAACCCACAGACCCTCTGAAGGAAGTGGATTGTTCCTGCAGGTCTCAGGAGACACCCCAAATGCTGTGGGAG
CCCAAACTGCAAACTGTGGAAGTGGGAAAGGGGAATAGTCAGCTCCTGAACACACATCCTCACTGGGGAACCTAAAGGTCTAGATCACAGGAGAAGATTTTGACCTTACTTGGAGCTGAGTCAATTTAGAGAGCCAAGTGACATACACTGCTAGAGAAAGCAG
CGCGAAAAGCCCTGTGGGCTCACTGGGTTCCCTAGCCATCCATTTCTGCCTTGCCTCACAGGGGTCCTTGAGGAGGGCTACCAGAGGCACTGGGAAATGGTCACAAAGAGAAGGAAACTTCCAGCTGAACTTTGTAACAATTTGAACAGATTGAGAAATCTCC
TGGCCAGAACCTGGGGGAGGGCATGAATCCAGTGTGCAGACTCCACAGGTGGGGAAGTACCAAAGCCCTACTTGCTTTTGCAGCTGGGAGGCAGGTAGCCTGGGGCAGAATCTCAGCCCTGCTCACCCACTGCCTTGAAACAGACTCGGTGCTGTTGTAGGGG
CTATGGTGGGAGTGAGACCAGCCCTTCGGGTTATGTGGGAGCTGGGTGAGGCCTATGACTTCAACTTCCCTGACAACCTGCATGGGATAAAAATCAGGGATAAAAATCACTACAGCTCTGTCCTCAGGAAGCCACATCCCTAGGAAAAGTGGGAGAGTACTAT
ATCAAGGGAACACCCTGTGGGACAAAAGAATCTGAACAACAGCCTTGAGCCCTAGACTTTCCCTCTGACAGAGCCTACCCAAAGGAGAAGGAACCAGAAAGCCCACTGTGGTAATATGACAAAACAAGGTTTTTTAACACCCCCCCAAAATCTCACTAGCTCA
CCAGCAATGGATCCAAATGAAGAAGGAATCCCTGATTTACCTGGAAAAGAATTCAGAAGATTAGTTATTAAGCTAATCAAGGAGGTACCAGAAAAGGCAAAGCCCAATTTAAGGAAGTGAAAAAAAAATGATAGAAGAAATGAGGGGAGAAATTTTCAATTAA
ATAGATAGGATAAATAACAAACAATCAAAACTTCAGGAAAAAATGGATGCACTTATAGAAATGCAAAATGCTCTGGAAAGTCTCAGCAATAGATTCCAACAAGTAAAAGAAAGAACTTCAGAGCTCGAAGACGAGGTTTTCAAATTAACCCAAACCAACAAAG
ACAAAGAAAAAAGAATAAGAAAATACAAGCAAAGCCTGCAAGAAATCTGGGATTATGTTAAACAACCAAACTAAGAATTATCAGCCTTCTTGAGGAAGAAGAGAAATC

>DEL1:GYPA-3':chromosome:GRCh37:4:145064236:145068255:1:GRCh38:4:144143083-144147102
1GATTCAGCACCTCACACAAGGTGTGAGGAAGCAGAGCCATATAGTGCCCTCTAGTGGCGTATCTGCACAATGGAAGGAAAAAATGTGAGCCCTGAAAATGCAACAGAAAAAAATATACGTCAGATTACATATTTCTTTTTCAGGCATTCCCTAATCTTCTTT
AATTTTTTCAAAAATATTTTTACTGAAGTACACATTCAGAATTATCATAAAATTCTACCTATGAGCTATATCATGCAAATTATTAGATAAATCCACATCTTTTAAGAATTTTGACTCTTTGCATAAAATAAAACAAGTACTTTTGAGGCATTTGATCAAACAT
AAAAAACTTATCCAATTTTCTTGATACTACATGTCTTTAGGCTATTTTTTGAATTGCCTGAGTCATTTCTATGACGTAGTTAAGAAGGATGAAGTTCAATGTTATAATCTAGGAAAGGCAACTTGGATCGATCAACACAAACTTATACCTTGCTTGTTTCATG
ACAGAGAACCATTCATTTTTGTCAATTACTTACCTCTCAACTATTTCAGGCTTAGATTATTCCTGAAGATGGTTGCATGCTAGTCTGATCTGCCAGTGGAGAAGTGTGCTTTTCATGTTTTGCATGGAGGAGTGGAGAACAGGGGAGGGGCAGGAAAGTGGAG
GCACAGTGGTTTATACTACTAGCCTCCGTGGTACCTCGTTGCCTTAGAATTGTCCAGCCTGCTTTGCCAGAGTAATCTAAGAACTTTCCCTGACCTTTGTTAGTTTATCATTTCAAAATTTTTTTAAATAAAAAAACAACTACAAGGTAAGATAAATCTAACG
CTCAAAATAACCCTGTTAGAGAGACAGAACAGATATTAGCATTCTTATTTTATATATGAGCAAACTATGGAATGGAAAAGAGATTATGGGTGTATTACCTGATTTGATTTGCCCTAAGTGTTTTTTAAACGTATGGATTTAGCTGCCAACATTTAAAAAGAAG
GTATATTTTAATTAAAATCAGGAATTGATTTAATTAAAAATTTGGCTCCTCTTACAGGAAGATCTGGCAAGAGTTAGCATGCAGGTCTACATTGTGACAATCCATGAGAGCTAAGGACTGGCTGTCTCTTGGGACAAATAGGCTTTCTTGAGTCTGGTAAGTG
CTCCACCTGATGCTTGCTTCCATTTCTTAGATTAGGTTTTACTGTGGTAAGGCCTCAAAATCTCAGTGTCTTGCAGCAACACTGGTTCATTTCCTGCTCACATTACATTTGGTCTATGATGGTGACAAGTTCCAGGATCAAGACTGAAGGAGCCACCTCCGTT
TGGAATATGCTGTTCTTGTTGCAAACTGGGGGAAAAAGGCCAAATAAGGAATGGCTTTTAATGCTTCCTCTCAGACATGGTATGTGTTATTTTTTTTTCTCACATTCTATTAACCAAAGTAAATCAGTGGCCAAGCTGACCTCAATGGGACAAGTAAATATAA
TTCTTCCAAATGGAAGTCCTGCAAGTCATAGTTCAATGGTGAGGATATATAAAACACTTACAGGAAGGGCCCTAATAATTGGGAGCAAAAACGCAATCCATCACATGCATTTATACTCTTTCTAAGCTTTTGGCATTTGGGTTTGGAAATCTAATTTATTTAG
GAGTCTTCTTCAACACCTTCTAGTGATGAGATTTCTTTAATCCAATTTTCAGTTTCCTTTCTTTAAAATTATAATGAGATGTGATGTGTGTTGAAGAAACCCCAGAAGTGAGAAACTCTCATCACTACAATGGTGACGTAGACAGTGATTAACTGTTCCCAGT
TGCCTCTAAGTCCATTGTGCTATACTGCTGGAATCAGGAATCATACATGATCTTTCTGCTGAGAATTGATAGGTCCAGCTTGTTCTACATCCAAATAATAATTCAACACATGATTTGTGAGCACCTACTGGGCACTGTGCCAAGTGTGGAAGATACAACCTTC
AGTAACTCAAGTTTCCCTTCAGGAACTGACAGCTTAATTGGGAAACAAATTAAGAAGGCAACTGCATTATGGAGTGTCAAGTGGATGATAGTAGTAAGCAAAGGGTGTTATAAGAGTACATAAGAGGGGTACCTAACTCAGACTTGGGGTTCTGGAAAGGTTT
TCCAGAGCAAGCAATGAGACCTAAAGGATGAGTAGACAAGTCACGGGAGAATTAAGAACATTTTAAGCAGAGAAAGCAGCATGAGCAATGGTCTGATATCAAGAAAGAGAGAGATAGTGCAAGAGTACACAATGTAGTAGTTTTCAAAACTGTTTGAAATTTT
AATCTCACATTTGTTCTTTTCAATGTCTAGGATATCAATTCATTCTGCCGTTTACCTTTGTAAACATGAATTTAGCCCTGTTTCTCTATATGAATACTAGCGGAAGTATATTGATAAAAGAAAAGGAACAAATATGTAGTCATTTCTAAGATTTATTTTTATT
AAAATGTAACACTCACAAACTGTAAGTGTATAACTCATATATATATATACACACACATATACACACACACACATACATGAGTATATGTGTGAAACTGTCACTCAAATCAAGACATAGAACATTTCAGATGTATTCCAGAGATCATGGTGGATGGGAGGCAGGA
CTGGATTGCAGCTCCCACTTGGACAGACAAAGCAGCGTGTGGAGGCTTGTATCATGAACTTTGACTGCAGGAATAAATCAGGAAAGCTGAGAGAACCCACAGACCCTCTGAAGGAAGTGAATTGCTCCTGCAGGTCTCAGGAGACACCCCAAATGCTGTGGGA
GCCCAAACTGCAAACTGTGGAAGTGGGAAAGGGGAATAGTCAGCTCCTGAACACACATCCTCACTGGGGAACCTAAAGGTCTAGATCACAGGAGAAGATTTTGACCTTACTTGGAGCTGAGTCAATTTAGAGAGCCAAGTGACATACACTGCTAGAGAAAGCA
GCGCGAAAAGCCCTGTGGGCTCACAGGGTTCCCTAGCTATCCATTTCTGCCTTGCCTCTCAGGGGTCCTTGAGGAGGGCTACCAGAGGCACTGGGAAATGGTCACAAAGAGAAGGAAACTTCCAGCTGAACTTTGTAACAATTTGAACAGATTGAGAAGTCTC
CTGGCCAGAACCTGGGGGAGGGCATGAATCCAGTGTGCAGACTCCACAGGTGGGGAAGTACCAAAGCCCTACTTGCTTTTGCAGCTGGGAGGCTGGTAGCCTGGGGCAAATTCTCAGCCCTGCTCACCCACTGCCTTGAAACAGACTCAGTGCTGTTGTAGGG
GCTATGGTGGGAGTGAGACCAGCCCTTCGGGTTATGTGGGAGCTGGGTGAGGCCTGTGACTTCAACTTCCCTGACAACCTGCATGGGATAAAAATCAGGGATAAAAATCACTACAGCTCTGCCCTCAGGAAGCCACATCCCTGGGAAAAGTGGGAGAGTACTA
CATCAAGGGAACACCCCGTGGGACAAAAGAATCTGAACAACAGCCTTGAGCCCTAGACTTTCCCTCTGACAGAGCCTACCCAAAGGAGAAGGAACCAGAAAGCCAACTCTGGTAATATGACAAAACAAGGTTTTTTAACACCCCCCCAAAATCTCACTAGCTC
ACCAGCAATGGATCCAAATGAAGAAGAAATCCCTGATTTACGTGGAAAAGAATTCAGAAGGTTAGTTATTAAGCTAATCAAGGAGGTACCAAAAAAGGCAAAGCCCAATTTAAGGAAGTGAAAAAAAAAAGATAAAATGATACAAGAAATGAGGGGAGAAATT
TTCAATTAAATAGATAGCATAAATAACAATCAAAACTTCAGGAAAAAATGGGTGCACTTATAGAAATGCAAAATGCTCTGGAAAGTCTCAGCAATAGATTCCAACAAGCAAAAGAAAGAACTTCAGAGCTCGAAGACGAGGTTTTCAAATTAACCCAAACCAA
CAAAGACAAAGAAAAAAGAATAAGAAAATACAAGCAAAGCCTGCAAGAAATCTGGGATTATGTTAAACAACCAAACTAAGAATTATCAGCCTTCTTGAGGAAGAAGAGA
```

The forward (GYP_DEL1_F10) and reverse (GYP_DEL1_R2B5) PCR primers are highlighted in yellow. The location of AciI restriction enzyme sites are highlighted in green (enzyme is non-palindromic and cuts at CCGC and GCGG); BsrBI is non-palindromic. For the PCR products produced using these primers (GYPE-GYPB region and GYPB-GYPB regions), only motifs in the GYPE-GYPB region are cut. The putative DEL1 breakpoint is shown highlighted in blue in the GYPE-GYPB and GYPB-GYPA regions. An alignment with additional feature highlights is shown below. The 12 bases highlighted in grey with blue-bolded text marks the location where all 3 sequences become homologous (see alignment below).

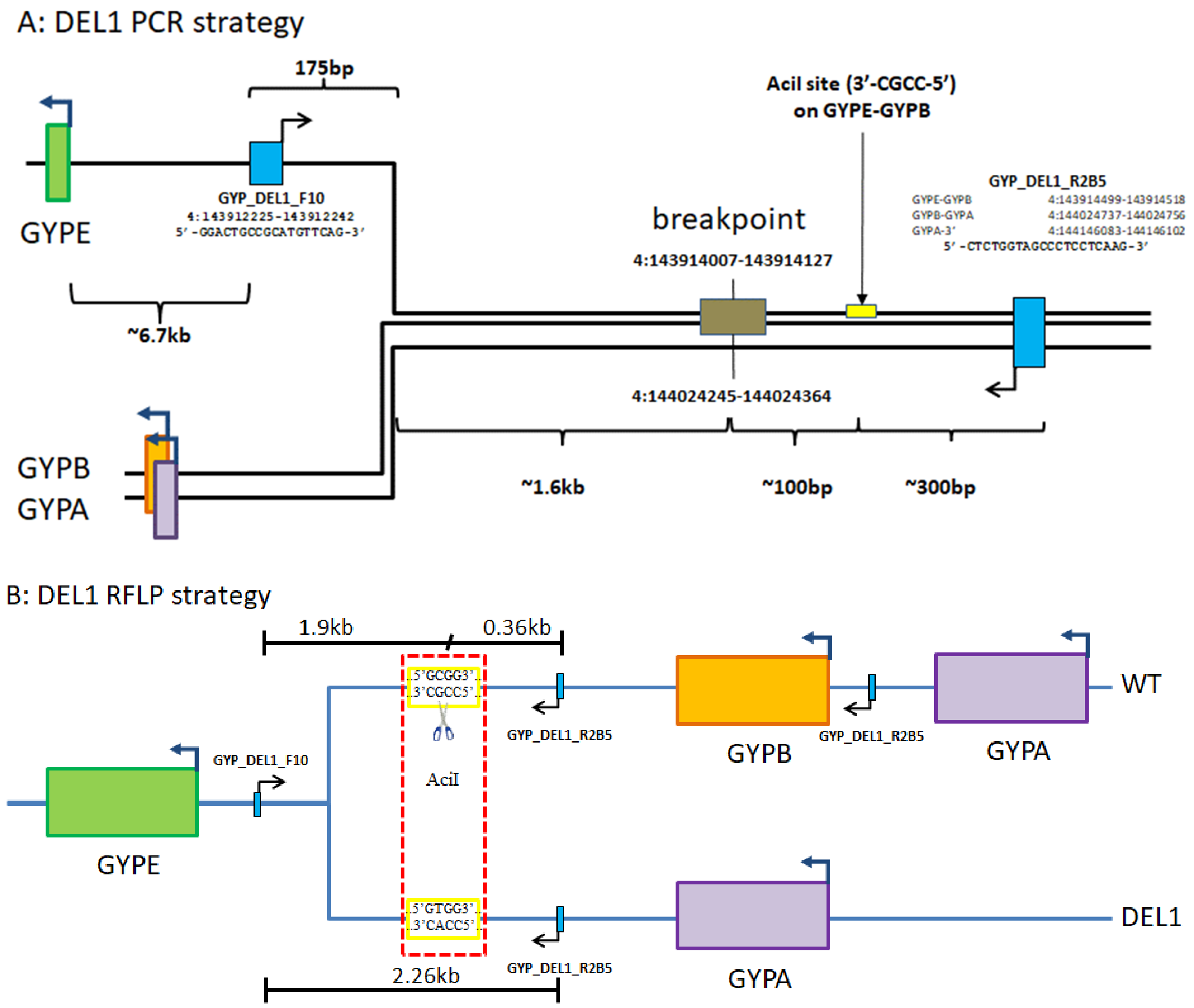

Supplementary Figure 2: Schematic representation of strategies for developing assays for the DEL1 GYP structural variant.

A: schematic of the alignment for the GYP SDUs showing the location of PCR primers, putative breakpoint and AciI restriction site.
On the upper left is shown the unique sequence at the 3' end of GYPE gene where the forward primer (GYP_DEL1_F10) for the PCR was positioned ~6.7kb from the GYPE start site and ~120 bases from the point of similarity (Sequence shown in "**Unique GYPE-GYPB region**" sequence alignment below). Also shown on the lower left are the regions of consensus between GYPB and GYPA at the point of departure from the GYPE sequence (Sequence shown in "**Complementary regions between GYPB-GYPA and GYPA-' that diverge from GYPE-GYPA**" sequence alignment below). A block of consensus between GYPE, B and A is shown (Sequences shown in "**Complementary regions between GYPE, GYPB, and GYPA**" sequence alignment below) with the locations of the DEL1 breakpoint, AciI restriction site and reverse primer (GYP_DEL_R2B5) shown.

B: Alternate schematic of the DEL1 RFLP assay showing a normal chromosome and the DEL1 chromosomes aligned. Genes and primers are indicated as well as the AciI restriction site and PCR-digestion fragment lengths.
Coordinates are given with respect to GRCh38

DEL1 breakpoint region pileup of the 4kb sequences shown above.

The alignment below was created using Clustal Omega (https://www.ebi.ac.uk/Tools/msa/clustalo/) and then manually finished and highlighted. All sequences are from the forward strand of the human reference genome.

The first section shows the unique sequences region ~6kb 3' to the GYPE gene. This is where the forward primer is located (highlighted in grey with red bolded text – GYP_DEL1_F10).
The [JOIN A] identifies where this sequences connects to the 3-sequences alignment in section 3.

Section 2 shows the alignment between the GYPB-GYPA and GYPA-3' sequences that diverge from the GYPB-GYPE sequence (see figure above). The [JOIN B] and [JOIN C] identify where these sequences connect to the 3-sequences alignment in section 3.

Section 3: Alignment of all 3 segmental duplication units sequences around the DEL1 breakpoint region (highlighted in green). The common reverse primer is shown highlighted in grey and red bolded text (GYP_DEL1_R2B5). Sites where there are differences between any 2 sequences are shown in yellow or blue highlights (in each case the top sequence's base is shown in blue and any matching bases also in blue, with different bases in yellow). The AciI restriction enzyme site is highlighted and identified. This will distinguish between the normal chromosome and DEL1 chromosome (as shown in the figure above). Due to the PCR and the position of the unique primer, the GYPA-3' sequence will not be amplified. This is shown here for completeness and was useful in the Sanger sequencing experiments.

SECTION 1: Unique GYPE-GYPB region

```
DEL1:GYPE-GYPB:chromosome        TTATAATGTTAAGCTACCTGAAGCATGCCTCCCCTGATTCTGTGGCATTCTTTGGAAGAGATCATGGTTGTATTCATAATATCTGTAGTCTTCATGTGCACGATCTCCAAGTGGCCGCTT
DEL1:GYPE-GYPB:chromosome        TCTCTGACCATCAACAAAATTGTCTGAATAATAATATCGGGAACCAGAGTCTCCATTTTCAACAGCAAAACTAACTTCCGTTACAAATGACTGAGAAGAACCATACTCTCTAGGGACATC
DEL1:GYPE-GYPB:chromosome        TGCTAGACTTCTGGCAAGATAAGAAGGTGCAGATAATCTGTTGCTTTCTCTTCTCATTATTTTATGAGGTGTTCTTTGAATTGGAGTTCTAAGGAAACTCTGGTTTCTTGAAACTACTCC
DEL1:GYPE-GYPB:chromosome        ATCTCCAGAAGTTGAAAAGGCATTAGGGCTTGAATCTGGAAGACAGATATCAGTTCGTCCTGGAATTTTTGGACCATGCTGGATGAATGAAGGCATAAGATTCCGAAGCAGAGGCGACGT
DEL1:GYPE-GYPB:chromosome        CTCCTTCTTCACGTACTTCCCCTGCACCTCGGCCGGCTTGGACACTTCGTTTTTGGTTTTCTGTCGGGACAGCATCCTTCTCGACCAGGCCCGAGGCCGCGCTGAACTGCCTCGCTAGGG
DEL1:GYPE-GYPB:chromosome        CTCCGCGCCGGGCGCCGGCCGTCTCCGCCGCCACCTCCGCCGCCACCTCCGCCGGCGCCGCCGCCTCCTTCCCTCCGGAGCCGCCGCCTCCGCCGCTGCCTGTCCGGAGCCTGGGGTCGC
DEL1:GYPE-GYPB:chromosome        CCGCAGGGACTGCCGCATGTTCAGGGCGCTAAACGCGCCGGCCGCCGCTCAGTCGCTGGTCACTTCCTTCCCGGAAGTCGGCCCGCTCTGCGACGCTGCTCGGGGACCCCTTGAGGAAAGCCCAGCGACGCCGGGCCAGC...[JOIN A]
                                       GYP_DEL1_F10     →
```

SECTION 2: Complementary regions between GYPB-GYPA and GYPA-3' that diverge from GYPE-GYPA sequence above

```
DEL1:GYPB-GYPA:chromosome        CAGATTCAGCACCTCATACAGGGTGTGAGGAAGCAGAGCCATATAGTTCCCTCTAGTGGCGTATCTGCACAATGGAAGGAAAAAATGTGAGCCCCGTAAATGCAACAGAAAAAAATATAC
DEL1:GYPA-3':chromosome          --GATTCAGCACCTCACACAAGGTGTGAGGAAGCAGAGCCATATAGTGCCCTCTAGTGGCGTATCTGCACAATGGAAGGAAAAAATGTGAGCCCTGAAAATGCAACAGAAAAAAATATAC
                                   ************** *** ************************** ********************************************** * ***********************

DEL1:GYPB-GYPA:chromosome        GTCAGATTACATTTTTCTCTTTCAGGCATTCCCTAATCTTCTTTAAATTTTTCAAAAATATTTTTACTGAAATACACATTCAGAATTATCATAAAATTCTGCCTAAGAGCTATATCATGC
DEL1:GYPA-3':chromosome          GTCAGATTACATATTTCTTTTTCAGGCATTCCCTAATCTTCTTTAATTTTTTCAAAAATATTTTTACTGAAGTACACATTCAGAATTATCATAAAATTCTACCTATGAGCTATATCATGC
                                 ************ ***** *************************** ************************ **************************** **** **************

DEL1:GYPB-GYPA:chromosome        AAATTATTAGACAAATCCACATCTTTTAAGAATTTTGACTC--TGCATAAAATAAAAGAAGTACTTTTGAGGCATTTGATCAAACATAAAAAACTTATCCAATTTTCCTGATACTACATG
DEL1:GYPA-3':chromosome          AAATTATTAGATAAATCCACATCTTTTAAGAATTTTGACTCTTTGCATAAAATAAAACAAGTACTTTTGAGGCATTTGATCAAACATAAAAAACTTATCCAATTTTCTTGATACTACATG
                                 *********** *****************************  ************** ************************************************* ************

DEL1:GYPB-GYPA:chromosome        TCTTTAGGCTATTTTTTGAATTGCCTGAGTCATTTCTATGACGTAGTTAAGAAGGATGAAGTTCAATGTTATAATCTAGGAAAGGCAACTTGGATCGATCAACACAAACTTATACCTTGC
DEL1:GYPA-3':chromosome          TCTTTAGGCTATTTTTTGAATTGCCTGAGTCATTTCTATGACGTAGTTAAGAAGGATGAAGTTCAATGTTATAATCTAGGAAAGGCAACTTGGATCGATCAACACAAACTTATACCTTGC
                                 ************************************************************************************************************************

DEL1:GYPB-GYPA:chromosome        TTGTTTCATGACAGAGAACCATTCATTTTTGTCAATTACTTACCTCTCAACTATTTCAGGCTTAGATTATTCCTGAAGATGGTTGCATGCTAGTCTGATCTGCCAGTGGAGAAGTGTGCT
DEL1:GYPA-3':chromosome          TTGTTTCATGACAGAGAACCATTCATTTTTGTCAATTACTTACCTCTCAACTATTTCAGGCTTAGATTATTCCTGAAGATGGTTGCATGCTAGTCTGATCTGCCAGTGGAGAAGTGTGCT
                                 ************************************************************************************************************************

DEL1:GYPB-GYPA:chromosome        TTTCATGTTTTGCATGGAGGAGTGGAGAACAGGGGAGGGGCAGGAAAGTGGAGGCACAGTGGTTTATACTACTATCCTCCTTGGCACCTCATCGCCTTAGAATTTTCCAGCCTCCTTTGC
DEL1:GYPA-3':chromosome          TTTCATGTTTTGCATGGAGGAGTGGAGAACAGGGGAGGGGCAGGAAAGTGGAGGCACAGTGGTTTATACTACTAGCCTCCGTGGTACCTCGTTGCCTTAGAATTGTCCAGCCTGCTTTGC
                                 ************************************************************************************************************************

DEL1:GYPB-GYPA:chromosome        CAGAGTAGTCTAAGAACTTTCCCTGACCTTTGTTTGTTTATCATTTGAAATTTTTTTTAAATAAAAAAACAACTACAAGGTAAGATAAATGTAACGCTCAAAATAACCCTGTTAGAGAGGCA...[JOIN B]
DEL1:GYPA-3':chromosome          CAGAGTAATCTAAGAACTTTCCCTGACCTTTGTTAGTTTATCATTTCAAAATTTTTTTAAATAAAAAAACAACTACAAGGTAAGATAAATCTAACGCTCAAAATAACCCTGTTAGAGAGACA...[JOIN C]
                                 ******* ************************** *********** *** *************************************** **************************** **
```

SECTION 3: Complementary regions between GYPE, GYPB, and GYPA

```
DEL1:GYPE-GYPB:chromosome        [JOIN A]...GCATTCTTATTTTATGTATGAGCAAACTATGGAATGGAAAAGAGATTATGAG-TTATTACTTGATTTGATTTGCCCTAAGTGTTTTTTAAACGTATGGATTTAGCG-
DEL1:GYPB-GYPA:chromosome        [JOIN B]...GCATTCTTATTTTAAATATGAGCAAACTATGGAATGGAAAAGAGACTATGAGTGTATTACTTGATTTGATTTGCCCTAAGTGTTTTTTAAACGTATGGATTTAGCTG
DEL1:GYPA-3':chromosome          [JOIN C]...GCATTCTTATTTTATATATGAGCAAACTATGGAATGGAAAAGAGATTATGGGTGTATTACCTGATTTGATTTGCCCTAAGTGTTTTTTAAACGTATGGATTTAGCTG
                                            **************  ***************************** **** *  ****** ********************************************

DEL1:GYPE-GYPB:chromosome        CCAACATTTAAAAAGAAGGTATATTTTAATTAAAAATCAGGAATTGATTTAATTTAAAATTTGGCTCCTCTTACAGGAAGATCTGGCAAGAGTTAGCGTGCAGTTCTACATTGTGACAAT
DEL1:GYPB-GYPA:chromosome        CTAACATTTAAAAAGAAGGTATATTTTAATTAAAAATCAGGAATTGATTTAATTAAAAATTTGGCTCCTGTTACCGGAAGATCTGGCAAGAGTTAGCATGCAGTTCTACATTGTGACAAT
DEL1:GYPA-3':chromosome          CCAACATTTAAAAAGAAGGTATATTTTAATTA-AAATCAGGAATTGATTTAATTAAAAATTTGGCTCCTCTTACAGGAAGATCTGGCAAGAGTTAGCATGCAGGTCTACATTGTGACAAT
                                 * ****************************** ********************* ************** **** ********************** ***** ****************

DEL1:GYPE-GYPB:chromosome        TCATGAGAGCTAAGGACTGGCTGTCTCTTGGGACAAATAGGCTTTCTTGAGTCTGGTAAGTGCTCCACTTGATGCTTGCTTCCATTTCTTAGATTAGGTTCTACTGTGGTAAGGCCTCAA
DEL1:GYPB-GYPA:chromosome        CCATGAGAGCTAAGGACTGGCTGTCTCTTGGGACAAATAGGCTTTCTTGAGTCTGGTAAGTGCTCCACCTGATGCTTGCTTCCATTTCTTAGATTAGGTTCTACTGTGGTAAGGCCTCAA
DEL1:GYPA-3':chromosome          CCATGAGAGCTAAGGACTGGCTGTCTCTTGGGACAAATAGGCTTTCTTGAGTCTGGTAAGTGCTCCACCTGATGCTTGCTTCCATTTCTTAGATTAGGTTTTACTGTGGTAAGGCCTCAA
                                  ******************************************************************* ******************************* *******************
```

SS2_Amuzu_et_al_Supplementary_File_2_DEL1_Pileup_GRCh37.docx

```
DEL1:GYPE-GYPB:chromosome      AATCTCAGTGTCTTGCAGCAACACTGGTTCATTTCCTGCTCACATTACATTTGGTCTATGACGGTGACAAGTTCCAGGATCCAGACTGAAGGAGCCACCCCCATTTGGAATATGCTGTTC
DEL1:GYPB-GYPA:chromosome      AATCTCAGTGTCTTGCAGCAACACTGGTTCATTTCCTGCTCACATTACATTTGGTCTATGACGGTGACAAGTTCCAGGATCCAGACTGAAGGAGCCACCCCCATTTGGAATATGCTGTTC
DEL1:GYPA-3':chromosome        AATCTCAGTGTCTTGCAGCAACACTGGTTCATTTCCTGCTCACATTACATTTGGTCTATGATGGTGACAAGTTCCAGGATCAAGACTGAAGGAGCCACCTCCGTTTGGAATATGCTGTTC
                               ************************************************************* ******************* ***************** ** *****************

DEL1:GYPE-GYPB:chromosome      TTGTTGCAAACTGGGGGAAAAAGGCCAGATAAGGAATGGCTTTTAATGCTTCCTCTCAGACTGTGTTATGG-----TTTTTTTCTCACATTCTATTAGCCAAAGTAAATCAGTGGCCAAG
DEL1:GYPB-GYPA:chromosome      TTGTTGCAAACTGGGGGAAAAAGGCCAAATAAGGAATGGCTTTTAATGCTTCCTCTCAGACATGGTATGTGTTATTTTTTTTTCTCATATTCTATTAGCCAAAGTAAATCAGTGGCCAAG
DEL1:GYPA-3':chromosome        TTGTTGCAAACTGGGGGAAAAAGGCCAAATAAGGAATGGCTTTTAATGCTTCCTCTCAGACATGGTATGTGTTATTTTTTTTTCTCACATTCTATTAACCAAAGTAAATCAGTGGCCAAG
                               *************************** *********************************   **    *     *********** ********* **********************

DEL1:GYPE-GYPB:chromosome      CTGACCTCAATGGGACAAGTAAATATAATTCTTCCAAATGGAAGTCCTGCAAGTCATAGGTCAATGGTGAGGATATATAAAACACTTACAGGAAGGGCCCTAATAATTGGGAGCAAAAAC
DEL1:GYPB-GYPA:chromosome      CTGACCTCAACGGGACAAGTAAATATAATTCTTCCAAATGGAAGTCCTCCAAGTCATAGGTCAATGGTGAGGATATATAAAACACTTACAGGAAGGGCCCTAATAATTGGGAGCGAAAAC
DEL1:GYPA-3':chromosome        CTGACCTCAATGGGACAAGTAAATATAATTCTTCCAAATGGAAGTCCTGCAAGTCATAGTTCAATGGTGAGGATATATAAAACACTTACAGGAAGGGCCCTAATAATTGGGAGCAAAAAC
                               ********** ************************************* ********** ****************************************************** *****

DEL1:GYPE-GYPB:chromosome      ACAATCCATCGCATTCATTTATACTCTTTCTAAGCTTTTGGCATTTGGGTTTGGAAATCTAATTTATTTAGGAGTCTTCTTCAACACCTTCTAGTGATGAGATTTCTTTAACCCAGTTTT
DEL1:GYPB-GYPA:chromosome      ACAATCCATCACATTCATTTATACTCTTTCTAAGCTTTTGGCATTTGGGTTTGGAAATCTAATTTATTTAGGAGTCTCCTTCAACACCTTCTAGTGATGAGATTTCTTTAACCCAGTTTT
DEL1:GYPA-3':chromosome        GCAATCCATCACATGCATTTATACTCTTTCTAAGCTTTTGGCATTTGGGTTTGGAAATCTAATTTATTTAGGAGTCTTCTTCAACACCTTCTAGTGATGAGATTTCTTTAATCCAATTTT
                                ********* *** ************************************************************** ********************************* *** ****

DEL1:GYPE-GYPB:chromosome      CAGTTTCCTTTCTTTAAAATTATAATGAGATGTGATGTGCGTTGAAGAAACCCCAGAAGTGAGAAACTCTCATCACTACAATGGTGATGTAGACAGTGGAAAACTGTTCCCAGTTGCCTC
DEL1:GYPB-GYPA:chromosome      CAGTTTCCTTTCTTTAAGATTATAATGAGATGTGATGTGCGTTGAAGAAACCCCAGAAGTGAGAAACTCTCATTACTACAATGGTGATGTAGACAGTGAAAAACTGTTCCCAGTTGCCTC
DEL1:GYPA-3':chromosome        CAGTTTCCTTTCTTTAAAATTATAATGAGATGTGATGTGTGTTGAAGAAACCCCAGAAGTGAGAAACTCTCATCACTACAATGGTGACGTAGACAGTGATTAACTGTTCCCAGTTGCCTC
                               ***************** ********************* ********************************* ************* **********   *******************

DEL1:GYPE-GYPB:chromosome      TAAGTCCATCGTGCTATACTGCTGGAATCAGGTATCATACATAATCTTTCTGCTGAGAATTGATAGGTCCAGCTTGTTCTACATCCAAATAATAATTCAACCAATGATTTGTGAGCACCT
DEL1:GYPB-GYPA:chromosome      TAAGTCCATTGTGCTATACTGCTGGAATCAGGAATCATACATGATCTTTCTGCTGAGAATTGATAGGTCCAGCTTGTTCTACATCCAAATAATAATTCAACAAATGATTTGTGAGCACCT
DEL1:GYPA-3':chromosome        TAAGTCCATTGTGCTATACTGCTGGAATCAGGAATCATACATGATCTTTCTGCTGAGAATTGATAGGTCCAGCTTGTTCTACATCCAAATAATAATTCAACACATGATTTGTGAGCACCT
                               ********* ********************** ********* **********************************************************  *****************

DEL1:GYPE-GYPB:chromosome      ACCGGGCACTGTGCCAAGTGTGGAAGACACAACCTTCAGTAACTCAAGTTTCCCTTCAGGAACTGACAGCTTAATTTGGAAACAAATAAATTAAGAAGGCAATTGCATTATGGAGTGTCA
DEL1:GYPB-GYPA:chromosome      ACCGGGCACTGTGCCAAGTGTGGAAGACACAACCTTCAGTAACTCAAGTTTCCCTTCAGGAACTGACAGCTTAATTGGGAAACAAATAAAGTAAGAAGGCAATTGCATTATGGAGTGTCA
DEL1:GYPA-3':chromosome        ACTGGGCACTGTGCCAAGTGTGGAAGATACAACCTTCAGTAACTCAAGTTTCCCTTCAGGAACTGACAGCTTAATTGGGAAACAAAT----TAAGAAGGCAACTGCATTATGGAGTGTCA
                               ** ************************ ************************************************ **********    *********** *****************

DEL1:GYPE-GYPB:chromosome      AGTGGATGATAGTAGTAAGCAAAGGGTGTTATAAGGGTACATAAGAGGGGTACCTAACTCAGGGTTGAGGTTCTGGAAAGGTTTTCCAGAGCAAGCAATGAGACCTAAAGGATGAGTAGA
DEL1:GYPB-GYPA:chromosome      AGTGGATGATAGTAGTAAGCAAAGGGTGTTATAAGAGTACATAAGAGGGGTACCTAACTCAGACTTGGGGTTCTGGAAAGGTTTTCCAGAGCAAGCA---AGACCTAAAGGATGAGTAGA
DEL1:GYPA-3':chromosome        AGTGGATGATAGTAGTAAGCAAAGGGTGTTATAAGAGTACATAAGAGGGGTACCTAACTCAGACTTGGGGTTCTGGAAAGGTTTTCCAGAGCAAGCAATGAGACCTAAAGGATGAGTAGA
                               *********************************** **************************  *** *****************************   ********************

DEL1:GYPE-GYPB:chromosome      CAAGTCACGGGAGAAGTAAGAACATTTTAAGCAGAGAAAACAGCATGAGCAATGGTCTGATATCAAGAAAGGCAGAGATAGTGCAAGAATACACAATGTAGTAGTTTTCAAAACTGTTTG
DEL1:GYPB-GYPA:chromosome      CAAGTCACGGGAGAAGTAAGAACATTTTAAGCAGAGAAAACAGCATGAGCAATGGTCTGATACCAAGAAAGAGAGAGATAGTGCAAGAATACACAATGTAGTAGTTTTCAAAACTGTTTG
DEL1:GYPA-3':chromosome        CAAGTCACGGGAGAATTAAGAACATTTTAAGCAGAGAAAGCAGCATGAGCAATGGTCTGATATCAAGAAAGAGAGAGATAGTGCAAGAGTACACAATGTAGTAGTTTTCAAAACTGTTTG
                               *************** *********************** ********************** ********  *************** *******************************

DEL1:GYPE-GYPB:chromosome      AAATTTTAATCTCACATTTGTTCTTTTCAATGTCTAGGATATCAATTCATTCTGCGATTTACCTTTGTAAAGATGAATTTAGCCCTGGTTCTCTATATGAATACTAGAGGAAGTATATTG
DEL1:GYPB-GYPA:chromosome      AAATTTTAATCTCACATTGGTTCTTTTCAATGTCTAGTATATCAATTCATTCTGCCGTTTACCTTTGTAAAGATGAATTTAGCCCTGGTTCTCTATATGAATACTAGAGGAAGTATACTG
DEL1:GYPA-3':chromosome        AAATTTTAATCTCACATTTGTTCTTTTCAATGTCTAGGATATCAATTCATTCTGCCGTTTACCTTTGTAAACATGAATTTAGCCCTGTTTCTCTATATGAATACTAGCGGAAGTATATTG
                               ****************** ****************** *****************  ************** *************** ******************* ********* **

DEL1:GYPE-GYPB:chromosome      ATAAAAGAAAAGGAACAAATATGTAGTCATTTCTAAGATTTATTTTTATTAAAATGTAACACTCACAAACTGTAAGTGTGTAACTCATATATATATATACACACATAT------ACACACACA
DEL1:GYPB-GYPA:chromosome      ATAAAAGAAAAGGAACAAATATGTAGTCATTTCTAAGATTTATTTTTATTAAAATGTAACACTCACAAACT--AAGTGTATAACTCATATGTATATATACACACATACACACACACACACACA
DEL1:GYPA-3':chromosome        ATAAAAGAAAAGGAACAAATATGTAGTCATTTCTAAGATTTATTTTTATTAAAATGTAACACTCACAAACTGTAAGTGTATAACTCATATATATATATACACACACATATACACACACACACA
                               ***********************************************************************  ****** ********** ************** *       *********

DEL1:GYPE-GYPB:chromosome      TACATGAGTATATGTGTGAAACTGTCACTCAAATCAAGACATAGAACATTTCAGATGTATTCCAGAGATCATGGTGGATGGGAGGCAGGACTGGATTGCAGCTCCCACTTGGACAGA
DEL1:GYPB-GYPA:chromosome      TACATGAGTATATGTGTGAAACTGTCACTCAAATCAAGACATAGAACATTTCAGATGTATTCCAGAGATCATGGTGGATGGGAGGCAGGACTGGATTGCAGCTCCCACTTGAACAGA
DEL1:GYPA-3':chromosome        TACATGAGTATATGTGTGAAACTGTCACTCAAATCAAGACATAGAACATTTCAGATGTATTCCAGAGATCATGGTGGATGGGAGGCAGGACTGGATTGCAGCTCCCACTTGGACAGA
                               *************************************************************************************************************** *****
                               <-------------------------------- Putative breakpoint region -------------------------------------------------->

DEL1:GYPE-GYPB:chromosome      CAAAGCAGCGTGTGGAGGCTTGTATCATGAACTTTGACTGCAGGAATAAATCAGGAACACTGAGAGAACCCACAGACCCTCAGAAAGAAGCGGATTGCTCCTGCAGGTCTCAGGAGACAC
DEL1:GYPB-GYPA:chromosome      CAAAGCAGCGTGTGGAGGCTTGTATCATGAACTTTGACTGCAGGAATAAATCAGGAAAGCTGAGAGAACCCACAGACCCTCTGAAGGAAGTGGATTGTTCCTGCAGGTCTCAGGAGACAC
DEL1:GYPA-3':chromosome        CAAAGCAGCGTGTGGAGGCTTGTATCATGAACTTTGACTGCAGGAATAAATCAGGAAAGCTGAGAGAACCCACAGACCCTCTGAAGGAAGTGAATTGCTCCTGCAGGTCTCAGGAGACAC
                               *********************************************************  ********************** *** **** * **** **********************
                                                                                                                             AciI site (CCGC on reverse strand)

DEL1:GYPE-GYPB:chromosome      CCCAAATGCTGTGGGAGCCCAAACTGCAAACTGTGGAAGTGGGAAAGGGGAATAGTCAGCTCCTGAACACACATCCTCACTGGGGAACCTAAAGGTCTAGATCACAGGAGAAGATTTTGA
DEL1:GYPB-GYPA:chromosome      CCCAAATGCTGTGGGAGCCCAAACTGCAAACTGTGGAAGTGGGAAAGGGGAATAGTCAGCTCCTGAACACACATCCTCACTGGGGAACCTAAAGGTCTAGATCACAGGAGAAGATTTTGA
DEL1:GYPA-3':chromosome        CCCAAATGCTGTGGGAGCCCAAACTGCAAACTGTGGAAGTGGGAAAGGGGAATAGTCAGCTCCTGAACACACATCCTCACTGGGGAACCTAAAGGTCTAGATCACAGGAGAAGATTTTGA
                               ************************************************************************************************************************

DEL1:GYPE-GYPB:chromosome      CCTTACTTGGAGCTGAGTCAATTTAGAGAGCCAAGTGACATACACTGCTAGAGAAAGCAGGGCGTAAGGCCCTGTGGGCTCACTGGGTTCCCTAGCCATCCATTTCTGCCTTGCCTCACG
DEL1:GYPB-GYPA:chromosome      CCTTACTTGGAGCTGAGTCAATTTAGAGAGCCAAGTGACATACACTGCTAGAGAAAGCAGCGCGAAAAGCCCTGTGGGCTCACTGGGTTCCCTAGCCATCCATTTCTGCCTTGCCTCACA
DEL1:GYPA-3':chromosome        CCTTACTTGGAGCTGAGTCAATTTAGAGAGCCAAGTGACATACACTGCTAGAGAAAGCAGCGCGAAAAGCCCTGTGGGCTCACAGGGTTCCCTAGCTATCCATTTCTGCCTTGCCTCTCA
                               ************************************************************ *** ** *************** ************ ******************** * 

DEL1:GYPE-GYPB:chromosome      GGGGTCCTTGAGGAGGGCTACCAGAGGCACTGGGAAATGGTCACAAAGAGAAGGAAACTTCCAGCTGAACTTTAAAACAATTTGAACAGATTGAGAAGTCTCCTAGCCAGAACTTGGGGG
DEL1:GYPB-GYPA:chromosome      GGGGTCCTTGAGGAGGGCTACCAGAGGCACTGGGAAATGGTCACAAAGAGAAGGAAACTTCCAGCTGAACTTTGTAACAATTTGAACAGATTGAGAAATCTCCTGGCCAGAACCTGGGGG
DEL1:GYPA-3':chromosome        GGGGTCCTTGAGGAGGGCTACCAGAGGCACTGGGAAATGGTCACAAAGAGAAGGAAACTTCCAGCTGAACTTTGTAACAATTTGAACAGATTGAGAAGTCTCCTGGCCAGAACCTGGGGG
                               *************************************************************************  ********************** ****** ******** *****
```

🡐 GYP_DEL1_R2B5 (5' – CTCTGGTAGCCCTCCTCAAG - '3)

```
DEL1:GYPE-GYPB:chromosome      AGGGCATGAATCCAGTGTGCAGACTCTACAGGTGGGGAAGTACCAAAGCCCTACTTGCTTTTGCAGCTGGGAGGCTGGTAGCCTGGGGCAAATTCTCAGCCCTGCTCACCCACTGCCTGG
DEL1:GYPB-GYPA:chromosome      AGGGCATGAATCCAGTGTGCAGACTCCACAGGTGGGGAAGTACCAAAGCCCTACTTGCTTTTGCAGCTGGGAGGCAGGTAGCCTGGGGCAGAATCTCAGCCCTGCTCACCCACTGCCTTG
DEL1:GYPA-3':chromosome        AGGGCATGAATCCAGTGTGCAGACTCCACAGGTGGGGAAGTACCAAAGCCCTACTTGCTTTTGCAGCTGGGAGGCTGGTAGCCTGGGGCAAATTCTCAGCCCTGCTCACCCACTGCCTTG
                               ************************** ************************************************ ************** * ************************* *

DEL1:GYPE-GYPB:chromosome      AAACAGACTCGGTGCTGTTGCAGGGGCTATGGTGGGAGTGAGACCAGCCCTTTGGGTTATGTGGGAGCTGGGTGAGGCCTGTGACTTCAACTTCCCTGACAACCTGCATGGGATAAAAAT
DEL1:GYPB-GYPA:chromosome      AAACAGACTCGGTGCTGTTGTAGGGGCTATGGTGGGAGTGAGACCAGCCCTTCGGGTTATGTGGGAGCTGGGTGAGGCCTATGACTTCAACTTCCCTGACAACCTGCATGGGATAAAAAT
DEL1:GYPA-3':chromosome        AAACAGACTCAGTGCTGTTGTAGGGGCTATGGTGGGAGTGAGACCAGCCCTTCGGGTTATGTGGGAGCTGGGTGAGGCCTGTGACTTCAACTTCCCTGACAACCTGCATGGGATAAAAAT
                               ********** ********* ******************************* *************************** ***************************************

DEL1:GYPE-GYPB:chromosome      CAGGGATAAAAATCACTACAGCTCTGCTCTCAGGAAGCCACATCCCTGGGAAAAGTGGGAGAGTACTACATCAAGGGAACACCCTGTGGGACAAAAGAATCTGAAGAACAGCCTTGAGCC
DEL1:GYPB-GYPA:chromosome      CAGGGATAAAAATCACTACAGCTCTGTCCTCAGGAAGCCACATCCCTAGGAAAAGTGGGAGAGTACTATATCAAGGGAACACCCTGTGGGACAAAAGAATCTGAACAACAGCCTTGAGCC
DEL1:GYPA-3':chromosome        CAGGGATAAAAATCACTACAGCTCTGCCCTCAGGAAGCCACATCCCTGGGAAAAGTGGGAGAGTACTACATCAAGGGAACACCCCGTGGGACAAAAGAATCTGAACAACAGCCTTGAGCC
                               **************************  ******************* ******************** *************** ******************** **************

DEL1:GYPE-GYPB:chromosome      CTAGACTTTCCCTCTGACAGAGCCTACCCAAAGGAGAAGGAACCAGAAAGCCAACTGTGGTAATATGACAAAACAAGGTTTTTTAACACCCCCCCAAAATCTCACTAGCTCACCAGCAAT
DEL1:GYPB-GYPA:chromosome      CTAGACTTTCCCTCTGACAGAGCCTACCCAAAGGAGAAGGAACCAGAAAGCCCACTGTGGTAATATGACAAAACAAGGTTTTTTAACACCCCCCCAAAATCTCACTAGCTCACCAGCAAT
DEL1:GYPA-3':chromosome        CTAGACTTTCCCTCTGACAGAGCCTACCCAAAGGAGAAGGAACCAGAAAGCCAACTCTGGTAATATGACAAAACAAGGTTTTTTAACACCCCCCCAAAATCTCACTAGCTCACCAGCAAT
                               **************************************************** *** ***************************************************************

DEL1:GYPE-GYPB:chromosome      GGATCCAAATGAAGAAGAAATCCCTGATTTACGTGGAAAAGAATTCAGAAGGTTAGTTATTAAGCTAATCAAGGAGGTACCAGAAAAGGCAAAGCCCAATTTAAGGAAGTGAAAAAAAAT
DEL1:GYPB-GYPA:chromosome      GGATCCAAATGAAGAAGGAATCCCTGATTTACCTGGAAAAGAATTCAGAAGATTAGTTATTAAGCTAATCAAGGAGGTACCAGAAAAGGCAAAGCCCAATTTAAGGAAGTGAAAAAAAAA
DEL1:GYPA-3':chromosome        GGATCCAAATGAAGAAGAAATCCCTGATTTACGTGGAAAAGAATTCAGAAGGTTAGTTATTAAGCTAATCAAGGAGGTACCAAAAAAGGCAAAGCCCAATTTAAGGAAGTGAAAAAAAAA
                               ***************** ************** ****************** ****************************** ************************************

DEL1:GYPE-GYPB:chromosome      ---------GATACAAGAAATGAGGGGAGAAAGTTTCAATTAAATAGATAGCATAAATAACAAACAATCAAAACTTCAGGAAAAAAATGGATGCGCTTATAGAAATGCAAAATGCTCTGG
DEL1:GYPB-GYPA:chromosome      --------TGATAGAAGAAATGAGGGGAGAAATTTTCAATTAAATAGATAGGATAAATAACAAACAATCAAAACTTCAGGA-AAAAATGGATGCACTTATAGAAATGCAAAATGCTCTGG
DEL1:GYPA-3':chromosome        AGATAAAATGATACAAGAAATGAGGGGAGAAATTTTCAATTAAATAGATAGCATAAATAACAA----TCAAAACTTCAGGA-AAAAATGGGTGCACTTATAGAAATGCAAAATGCTCTGG
                                        **** ****************** ****************** ***********    ************** ******** *** *************************

DEL1:GYPE-GYPB:chromosome      AAAGTCTCAGCAATAGATTCCAACAAGCAAAAGAAAGAACTTCAGAGCTCGAAGACGAGGTTTTCAAATTAACCCAAACCAACAAAGACAAAGAAAAAAGAATAAGAAAATACAAGGAAA
DEL1:GYPB-GYPA:chromosome      AAAGTCTCAGCAATAGATTCCAACAAGTAAAAGAAAGAACTTCAGAGCTCGAAGACGAGGTTTTCAAATTAACCCAAACCAACAAAGACAAAGAAAAAAGAATAAGAAAATACAAGCAAA
DEL1:GYPA-3':chromosome        AAAGTCTCAGCAATAGATTCCAACAAGCAAAAGAAAGAACTTCAGAGCTCGAAGACGAGGTTTTCAAATTAACCCAAACCAACAAAGACAAAGAAAAAAGAATAAGAAAATACAAGCAAA
                               *************************** **************************************************************************************** ***

DEL1:GYPE-GYPB:chromosome      GCCTGCAAGAAATCTGGGATTATGTTAAACAACCAAACTAAGAATTATCAGCCTTCTTGAGGAAGAAGAGAAATC
DEL1:GYPB-GYPA:chromosome      GCCTGCAAGAAATCTGGGATTATGTTAAACAACCAAACTAAGAATTATCAGCCTTCTTGAGGAAGAAGAGAAATC
DEL1:GYPA-3':chromosome        GCCTGCAAGAAATCTGGGATTATGTTAAACAACCAAACTAAGAATTATCAGCCTTCTTGAGGAAGAAGAGA----
                               ***********************************************************************
```

Amuzu *et al.*, Supplementary File 2: DEL2 pile-up.

Sequences of GYPE, B, A around the DEL2 breakpoint in FASTA format.
Sequences were downloaded from Ensembl and are with respect to GRCH37 (http://grch37.ensembl.org/index.html)

>DEL2:5'-GYPE:GRCh37:4:144790086:144794131:GRCh38:4:143868933:143872978:1
GTCTCAAGTACTTGATATATCAACACACTATTGCCCCTTTTTTTCACACCTGAAGTTTGTAGAATAAACCACTAAAAATTTCATAGTATCTGAGGAGAATGGATCTCTCCAGATGATGTTTTCTTGCCTGGGGCCAATATGTGCACCATAGAGTCAGTCCCCT
TTATCTGCCATAAAACTTTGTTTATGAAGCATTGCTTAATCAATCTTCACATGGTAGGACGATACAGGCAAAAGCTGAGGTCTTGCTATACATCTCTTTCAAAGTTTTCCGTCAAGGCCATTTTGCTTTTAGTCACTTTTTTTTAATATTTTCGGCAGCAGTT
ATTTATTTCATTTCATTGTATTTGACCCATCCAAATGTTTAACATGGTTTTATAAAAGTAACTTTATTTTCAGCTTGTCTGTAGATTCAATTTTCTTGTAGTTGCTGGTATAAGTAATAGGGAAAAATGTATCAGTCTTATACAATAAAGAAGTTCAGATGAG
CGTGTGTGGGAGTGTGTGATATAGGAAGAAGCAATCGGTATATAACAACATATGTCTGCCTGCTACGCTTTTAATACTTGTTAGAGTTTTAGTTTCTATGAGTATCTTGGAGGTGCTGGAATATATATGCAAATTTCCTTCTAAAAGATTGCACCAAGTTACA
TTACCATAACAACAATAAGAATTTACTGCATCCTTAGCACATTGAATATTATATTAGAAATACCACTTTGTAGGTTTCATAGATAATGGATAAATTTTCTGTTGTTTTAATTTACGTTTTAAAAGTAGTGAGGTTGAAAATGATGCAATTTGAAATGCATGTG
CATAAGAAGTTGAATAGAAGTTTATTGGTCAGAATGTGGAATGAGTTTTGAACAAGTTAAATGTTTTGAAAAACAGTATGAATGTACTGAATACTTTTGAGGTGTGATCTTCTAATTGCTCTAGCAAATTGAATTGGTCATAAGAAAACGTTTGAATTTTTCC
GATCAGTCAGTCATTGTATTAGTTCATTCCCATGCTGCTATAAAGAAGTGCCTGAGACTGGGTAATTTATAAAGGAAAGAAGTTTAATTGACTCACAATTCTGCGTGGCTGAGAAGGTTTCAGGAAACTTACAATCGTGGCAGAAGGGGAAGCAAACACATCG
TTTTTCACATGATGGCAAAAGTAAATGGGGAAGCCCCTTATAAAACCATCAGATCTCATGAGAATTTGCTCACTATCATGAAAATAGCATGGGGAAACTGCCACAATGATTCAATTACCTCCCACTACATTCCTCCCACAACACATGGGGATTGTGGGAACT
ACAATTCAAGATGAGATTTGGGTGGGGACACAGCCAAACCACATCACTATGCCCCTGACCCCTCCCAAATCTCATGTCCTCACATTTCAAAACACAATCATGCCTTCCAAACAGTCCCCCAAAGTCTAAACTCATTCCAGCATTAACTCAAAAGTCCAAGTCC
AAAGTCTCATCTGAGACAAGGCAAGTCCCTTCCACCTATGAGCCTGTAAAATCAAAAGCAAGTTAGTTACTTCCTAGATACAATGAGGGTATAGGCAATGGGTAAATGCACCCATTCTAAATAGGATAAATTGGCCAAAAACAGGAGCTACAGGCCCATGCAA
GTCTGAAATCCAGTGGAGCAGTAATTAAATCTTAAAGCATCTTAATAATCTCTTTTGACTCCATGTCTCACATCCAGGTAATGCTGTTGCAAGAGGTGGGCTCCCACAGTCTTGGGAAGCTCAGCTCCTGTGGCTTTGCATGGTAAACCCCCCCGCCTCGGCT
GCTTTCACAGGCTGTCTGTTATCTAGTTCCAAAGTCACTTCTGCATTTTTAGGTATCCTTATAGCAGCACCCCACCTCTAGTACCAACTTACTGTATTAGTCTGTTCTCATGCTGTTATAAAAAACTGCCCAAGACTGTGTAATTTATAAAGGAAAGAGGTTT
TATTGATCTACAGTTTTGCATGGCTGGGAAGGTCTCAGGATACTTACAATCATGACCAAAGGGGAAACAAACACATCTTTCTTACATAGTGGCAGGAAGGAGAAGAATGAGAGCTGAGTGAAGGGGGAAGCTCCTTTATAAAACTATCAGATTATGTGAGAAT
TTACTCACTATCATGAGAATAGCACAGGGGAAACCACCGCAATGATTCAAGTACCTCCCACTGGGTTCCTCCCATGACATGTGGGGATTATTGGAACTACAATTCAAGATGAGATTTGAGTGGGAACACAGCCAAACCATATCAGTCATTCCACATATTGAGT
GATTTCTCTCTTCTATTCAGTATTCTTCCACAGAGAGGGACCCATAGTCATTACCTTCAAGGAACCTAAATCCTGGTGTTTTTATGGTTACGGGTGGCATATAATATACAGGTAAGCCAATGTTACAGGATGTTGTGGAACTATTCCTATTCTTCCATAAGTA
GCCACTAGGACCACGGCTGAAACCAAGAGGCACTGATTACCACCTCACTCTAAAGAGAATTACTGAGTTTGTGTTCACTGCTGGGGCCAGATCTCTTTGCAGGGCTATGTTAGGCTTTACACAATGTGACCCTCTAGTCTGTACAAGGAGAGGAATGCAGAGG
GAAAGCGATTCACTGGAGTCTCTCGTCAGTGACCTGGTGGTGAATATATGGTAATGGGTAAATCCCACTTCACCTAATGAAGAAAAGGAGAAGGATGCACTTCATTCTTAGAATCTAAGCAGGAACAGGAAAGGGAAAATCTAGGAGAAAAGCTTACAAAGAC
TGTAAATGCCTTCAAGAAGGTGAGAGAAACTAATATTTGGTTTACTGTCAGCAGTCTGTTCGGGCGGCATTTTGCCAATATAGCCCTATCCTAAGGGAAAGGGAATTGTGGTTGGACAACCGAGTTCTGAGAAAGGCAGTGATTGAGCAGCTGAATTCCATCC
ATTTGGTGGGTAAGGACCTCAGAGTATTCAAAGTATCAAATGGAATGTGACTGAAGAGTTATCTTGCTGATCTTACCCAGGAGGACTGTCCCCTAGGCAAGGGGACACCAAACACAATGAGGCATGGGATAAGGATTTCGTGATGAGAATCGGGCAGAACTGG
AGTTTAATCCTCATGTCATGGTAAGGCCCTCTGAAATTCTCTCTACGCCTTTGAGAGACTCAGCATTTAGTTTCTGACTCCTAGAGTCCCTTAGTAGCCAGTCAACGTAAGCCAAGTTAGTAGCTACAGCCGTCAGGCACACAATTACACCATGAACAGTGAA
GTAAAAAAATGTCCTTTCTTTGCTTTCTTCCTCTAATCTCAATCCCAGAGGCATGAAAACCTAGAAAAAAGAAAGTAGCTGTATAGCTTTCATTTACATATAAGATTGATGTTTTGAAATTTTAATAACAAAAACAACCAGAAAGTTGCCTATCTCCCCAAGA
TGTTTCTAGGGAATAGGAATAAGAAATAGGAAAAGTATATTCCATGCAGTATAAGCAAATTTAAAAAATTAATTTTTTTTACCCTACTGAATTGAGACTTGCCAATAACCAGTTATACAAATAAAGAGGCTAGTCCTGGGAGCTGAGTGCTATGAAAAAAATA
CAGGTAGAAAATGAAAGAGAAATTAATGGGAAGGAGGGAAGAAGACTACAAGCTGCTTTGCGTTGGGTAGGCAGGGAAGACCTATTTCATTCAATGAAATTTTACTGAGTTTTCCTGTGTTCTAGCCATTATTCTGCGCTCTGAGGATACATCGTAAACAAAG
CAGATAAAAAAATACAGTCATCAGAATTTTCCTTGAAGGTGTGAGACAGGAATAATATGGAGGCAGTCAAGCAAAGATATGAAGCAAAAAAAGGAGAAAAAATTAGAACAAAGTTCCTAAGAAAGAGCAAGCTTAGTAAGTTGGAGTTTTGGGGAAAAGTTCAG
TGGTTGCAACCAAATGACAGGAGAGAGTGGGAGAGGGTTGAAGGGGGCAGGCCAGGTGAGCTGAGCATGTGGACAGGTCAGAACTTCAGAGAGCATGCTAACAAGTGGTGATTTTATTCTGGTGCAGTGAAAAA

>DEL2:GYPE-GYPB::GRCh37:4:144910881:144914932:GRCh38:4:143989728:143993779:1
CTGTGATTGCCTCTAGCAAGCAGTCTCAAGTACTTGATATATAAACACATTATTGCCCCTTTTCTTCACACTTGAAGTTTGTAGAATAAACCACTAAAAATATCACAGTATCTGAGGAGAAAGGACCTCTCCAGATGATGTTTTCTTACCTGGGGCCAATATG
TGCACCATAGAGTCCCCTCTATCTGCCATAAAACTTTGTTTATTGCTTTAATCAATCTTCACATGGTAGGACGATACAGGCAAAAGCTGAGGTCTTCCTATACATCTCTTTCAAAGTTTTCCTTCAAGACCATTTTGCTTTTGGTCACTTTTTCTTTATATTT
TCAGCAGCAGTTATTTATTTCATTTCATTCTATTTGACCCATCCAAATGTTTAACATGGTTTTATAAAAGTAACTTTATTTTTAGCTTGTCTGTAGATTCAATTTTCTTGTAGTTGCTGGTATAAGTAATAGGGAAAAATGTATCAGTCTTATACAATAAAGA
AGTTCAGATGAGTGTGTGATATAGGAAGAAGCAATCGGTATGTAACAACATATGTCTGCCTGCTACGCTTTTAATACTTGTTAGAGTTTTAGTTTCTATGAGTATCTTGGAGGTGCTGGAATATATATGCAAATTTCCTTCTAAAAGATTGCACCAAGTTACA
TTACCATAACAACAATAAGAATTTACTGCAGCCTTAGCACATTGGATATTATCTTAGAAATACCACTTTGTAGGTTTCATAGATAATGGATAAATTTTCTGTTGTTTTAATTTACATTTTAAAAGGAGTGAGGTTGAAAATGATGCAATTTGAAATGCATGTG
CATAAGAAGTTGAATAGAAGTTTATTGGTCAGAATGTGAAATGAGTTTTGAACAAGTTAAATGTTTTGAAAAACAGTATGAATGTACTGAATACTTTTGAGGTGTGATCTTCTAATTGCTCTAGCAAATTGAATTGGTCATGAGAAAACGTTTGAATTTTTCC
AGTCAGTCAGTCATTGTATTAGTTCATTCCCATGCTGCTATAAAGAAGTGCCTGAGACTGGGTAATTTATAAAGGAAAGAAGTTTAATTGACTCACAATTCTGCGTGGCTGAGAAGGTTTCAGGAAACTTACAATCGTGGCAGAAGGGGAAGCAAACACATCC
TTTTTCACATGATGGCAAAAGTAAATGGGGAAGCCCCTTATAAAACCATCAGATTTCATGAGAATTTGCTCACTATCATGAAAATAGCATGGGGAAACTGCCACAATGATTCAATTACCTCCCACTACATTCCTCCCACAACACGTGGGGATTGTGGGAACT
ACAATTCAAGAGGAGATTTGGGTGGGGACACAGCCAAACCACATCACTATGCCCCTGACCCCTCCCAAATCTCATGTCCTCACATTTCAAAACACAATCATGCCTTCCAAACAGTCCCCCAAAGTCTAAACTCATTCCAGCATTAACTCAAAAGTCCAAGTCC
AAAGTCTCATCTGAGACAAGGCAAGTCCCTTCCACCTATGAGCCTGTAAAATCAAAAGCAAGTTAGTTACTTCCTAGATACAATGAGGGTATAGGCAATGGGTAAATGCACCCACTCTAAATAGGAGCAATTGGCCAAAAACAGGGGCTACAGGCCCATGCAA
GTCTGAAATCCAGTGGGGCAGTAATTAAATCTTAAAGCACCTTAATAATCTCTTTGGACTCCATGTCTCACATCCAGTTAATGCTGATGCAAGAGGTGGGCTCCCACAGTCTTGGGAAGCT**CCGCTC**CTGTGGCTTTGCATAGTACAACC**CCGCTC**TCGGCTG
CTTTCACAGGCTGTCTGTTATCCAGTTCCAAAGTCACTTCTGCATTTTTAGGTATCCTTATAGCAGCACCCCACCTCTAGTACCAACTTACTGTATTAGTCTGTTCTCATGCTGCTATAAAAAACTGCCCAAGACTGTGTAATTTATAAAGGAAAGAGGTTTA
GTTGATCTACAGTTTTGCATGGCTTGGAAGGTCTTAGGATACTTACAATCATGACCAAAGGGGAAACAAACACATCTTTCTTACATAGTGGCAGGAAGGAGAAGAATGAGAGCTGAGTGAAGGGGGAAGCTCCTTTATAAAACTATCAGATTATGTGAGAATT
TACTCACTATCATGAGAATAGCACAGGGGAAACCACCGCAATGATTCAAGTACCTCCCACTGGGTTCCTCCCATGACATGTGGGGATTATTGGAACTACAATTCAAGATGAGATTTGAGTGGGAACACAGTCAAACCATATTAGTCATTCCACATATTGAGTG
ATTTCTCTCTTCTATTCACTATTCTTCCACAGAGAGGGACCCATAGTCATTACCTTCAAGGAACCTAAATCCTGGCATCTTTATTGTTATGGGTGGTGTATAATATACAGGTAAGCCAATGTTACAGGATGTTGTGAGACTATTCCTATCCTTCCATAATTAG
CCACTAGGACCACAGCTGAAACCAAGAGGCACTGATTACCCCCTCACTCTAAAGAGAATTACTGAGCTTGTGCTCACTGCGGGGGCCAGATCTTTTTGCAGGGCTATGTTAGGCTTTACACAATGTGACCCTCTAGTCTGTACAAGGAGAGGAATGCAGAGGG
GAAGAGATTCACTGGAGTCTCTCGTCAGTGACCTGGTGGTGAATATATGGTAATGGGTAAATCCCACTTCACCTGATGAAGAAAAGGAGAAGGATGCACTTCATTCTTAGAATCTAAGCAGGAACGGGAAAGGGAAAATTAGGAGAAAAGCTTACAAAGACTG
TAAATGCCTTCAAGAAGGTGAGAGAAACTAATATTTGGTTTACTGTCAGCAGGCTGTTCGGGCGGCATTTTGCCAATATAGCCCTATCCCTACGTGAGTAGGGGAAGGGAATTGTGGTTGGACAACAGAGTTCTGAGAAAGGCAGTGATTGAGCAGCTGAACT
CCATCCATTTGGTGGGTAAGGACCCCAGAGTATTCAAAGTATCAAATGGAATGTGACTGAAGTGTTATCTTGCTGATGTTACCCAGGAAAACTGTCCCCTAGGCAAGGGGACACCAAACACAAAGAGGCACGGGATAAGGATTTCGTGACGAGAATTGGGCAG
AACTGGAGTTTAATCCCCATGTCATGGTAAGGCCCTCTGAAATTCTCTCTACACCTTTGAGACACTCAGCATTTAGATTCTGACTCCTAGAGTCCCTTAGTGGTCAGTCAATGTAAGCCAAGTTAGTAGCTACAGCCGTTAGGCACACGATTACACTGTGAAC
AGTGAAGTAAAAAAATGTCCTTTCTTTGCTTTCTTTCTCTAATCCCAATCCCAGAGGCATGAAAACCTAGAAAAAAGAAAGTAGCTGTATAGCTTTCATTTACATATAAGATTGGTGTTTTGAAATTTTAATAACAAAAAAGACCAGAAAGTTTCGTATCTCCC
CAAGATGTTTCTAGGGAATAGAAATAAGAAATAGGAAAAGTACATTCCATGCAGTATGAGCAAATTTAAAGAATTAATTTTTTTTACCCTACTGAATTGAGACTTGCCAATAACCAGTTATACACATAAAGAGGGTAGTCTTGGGAGCTGAGTGCTATGAAAA
AAATACAGGAAGAAAATGAAAGAGAAATTAATGGGAAGGAAGGAAGAAGACTACAAGCTGCTTTGCGTTGGGTAGGCAGGGAAGACCTATTTCATTCAATGAAATCTTACTGAGTTTTCCTGTGTTCTAGCCATTATTCTGGGCTCTGAGGATGCATAGTAAA
CAAAGCAGATGAAAAATACTGTCATCAGAATTTTCTTTGAAGGTGTGAGACAGGAATAATATGGAGGCAGTCAAGCAAAGATATGAAGCAAAAAAAGGAGAAAAAATTAGAACAAAGTTCCTAAGAAAGGAGCAAGCTTAGTATGTTGGAGTTTTGGGGAAAA
GTTCAGTGGTTGCAACCTAAAGACAGGAGAGAGTGGGAGCAGGTTGAGAGGGGCAGGCCAGGTGACCTGAGCATGTGGACAGGTCAGAACTTCAGAGAGCATGCTAACAAGTGGTGATTTTATTCTGGTGCAGTGAAAAA

>DEL2:GYPB-GYPA:GRCh37:4:145014146:145018188:GRCh38:4:144092993:144097035:1
GCAGTCTCAAGTACTTGATATATCAACACACTATTGCCCCTTTTTTTCACACCTGAAGTTTGTAGAATAAACCACTAAAAATTTCATAGTATCTGAGGAGAATGGATCTCTCCAGATGATGTTTTCTTGCCTGGGGCCAATATGTGCACCATAGAGTCCCCTC
TATCTGCCATAAAACTTTGTTTATGAAGCATTGCTTTAATCAATCTTCACATGGTAGGACGATACAGGCAAAAGCTGAGGTCTTGCTATACATCTCTTTCAAAGTTTTCCTTCAAGACCATTTTGCTTTTAGTCACTTTTTTTTAATATTTTTGGCAGCAGTT
ATTTATTTCATTTCATTCTATTTGACCCATCCAAATGTTTAACATGGTTTTATAAAAGTAACTTTATTTTCAGCTTGTCTGTAGGTTCAGTTTTCTTGTAGTTGCTAGTATAAGTAATAGGGAAAAATGTATCAGTCTTATACAATAAAGACGTTCAGATGAG
TGTGTGTGGGAGTGTGTGATATAGGAAGAAGCAATCGGTATGTAACAACATATGTCTGCCTGCTACGCTTTTAATACTTGTTAGAGTTTTAGTTTCTATGAGTATCTTGGAGGTACTGGAATATATATGCAAATTTCCTTCTAAAAGATTGCACCAACTTACA
TTGCCATAACAACAATAAGAATTTACTGCAACCTTAGCACGTTGGATATTATATAAGAAATACCACTTAGTAGGTTTCATAGATAATGGATAAATTTTCTGTTGTTTTAATTTACATTTTGAAAGTAGTGAAGTTGAAAGTGACGCTATTTGAAATGCATGTG
CATAAGAAGTTGAATAGAAGTTTATTAGTCAGAAGGTGGAATGAGTTTTGAACAAGTTAATTGTTTTGAAAAACAGTATGAATGTACTGAATACTTTTGAGGTGTGATCTTCTAATTGCTCTAGCAAATTGTATTGGTCATAAGAAAATGTTTGAATTTTTCC
AGTCAGTCAGTCATTGTATTAGTTCATTCCCATGCTGCTATAAAGAAGTGCCTGAGACTGGGTAATTTATAAAGGAAAGAAGTTTAATTGACTCACAATTCTGCGTGGCTGAGAAGGTTTCAGGAAACTTACAATCGTGGCAGAAGGGGAAGCAAACACATCC
TTTTTCACATGATGACAGAAGTAAATGGGGAAGAGCCTTATAAAACCATCAGATCTCATGAGAATTTGCTCACTATCATGAAAATAGCATGGGGGAAACAGCCACAATGATTCAATTACCTCCCACTACATTCCTCCCACAACACGTGGGGATTGTGGGAACT
ACAATTCAAGATGAGATTTGGGTGGGGACACAGCCAAACCACATCACTATGCCCCTGACCCCTCGCAAATCTCATGTCCTCACATTTCAAAACACAATCATGCCTTCCAAACGGTCCCCCAAAGTCTTAACTCATTCTAGCATTAACTCAAAAGCCCAAGTCC
AAAGTCTCATCTGAGACAAGGCAAGTACCTTCCACCTATGAGCCTGTAAAATCAAAAGCAAGTTAATTACTTCCTAGATACAATGAGGGTATAGGCAATGGGTAAATGCACCCATTCTAAATAGGATAAATTGGCCAAAAACAGGAGCTACAGGCCCATGCAA
GTCTGAAATCCAGTGGGGCAGTAATTAAATCTTAAAGCACCTTAATAATCTCTTTTAACTCCATGTCTCACATCCATGTAATGCTGATGCAAGAGGTGGGTTCCCACAGTCTTGGGAAGCTCTGCTCCTGTGGCTTTGCATGGTACAACCCCCCTCTCGGCTG
CTTTCACGGGCTGTTATCCAATTCCAAAGTCACTTCTGCATTTTTAGGTATCCTTATAGCAGCACCCCACCTCTAGTACCAACTTACTGTATTAGTCTGTCTCATGCTGCTATAAAAAACTTCCCAAGACTGTGTAATTTATAAAGAGGTTTAATTGATCTAC
AGTTTTGCATGGCTGGGAAGGTCTCAGGATACTTACAATCATGACCAAAGGGGAAACAAACACATCTTTCTTACATAGTGGCAGGAAGGAGAAGAATGAGAGCTGAGTGAAGGGGGAAGCTCCTTTATAAAACTATCAGATTATGTGAGAATTTATTCACTCT
CATGAGAATAGCATAGGGGAAACCACCGCAATGATTCAAGTACCTCCCACTGGGTTCCTCCCATGACACGTGGGGATTATTGGAACTACAATTCAAGATGAGATTTGAGTGGGAACACAGCCAAACCATATCAGTCATTCCACATATTGAGTGATTTCTCTCT
TCTATTCACTATTCTTCCACAGAGAGGGACCTGTAGTCATTACCTTCAAGGAACCTAAATCCTGGTGTCTTTATGGTTATGGGTGGCATATAATATACAGGTAAGCCAATGTTACAGGATGTTGTGAGACTATTCCTGTTCTTCCATAATTAGCCACTAGGAC
CACGGCTGAAACCAAGAGGCACTGATTACCGCCTCACTCTAAAGAGAATTACTGAGTTTGTGCTCACTGCTGGGGCCAGATCTCTTTGCAGGGCTATGTTTGGCTTTACACAATGTGACCCTCTAGTCTGTACAAGGAGAGGAATGCAGAGGGAAAGAGATTC
ACTGGAGTCTCTCGTCAGTGACCTGGTGGTGAATATATGGTAATGGGTAAATCCCACTTCACCTGATGAAGAAAAGGAGAAGGATGCACTTCATTCTTAGAATCTAAGCAGGAACAGGAAAGGGAAAATCTAGGAGAAAAGCTTACAAAGACTAAATGTCTTC
AAGAAGGTGAGAGAAACTAATATTTGGTTTACTGTCAGCAGGCTGTTTGGGCGGCATTTTGCCAATATTAGCCCTATCCCTACGTGAGTAGGGGAAAGGGAATTGTGGTTGGACAACCGAGTTCTGAGAAAGGCAGTGATTGAGCAGCTGAACTCCATCCATT
TGGTGGGTAAGGACCCCAGAGTATTCAAAGGATCAAATGGAATGTGACTGAAGAGTTATCTTGCCGATCTTACCCAGGAAGACTGTCCCCTAGGCAAGGGGACACCAAACACAAAGAGGCATGGGATAAGGATTTCAAGATGAGAGTTGGGCAGAACTGGAGT
TTAATCCCCATGTCATGGTAAGGCCCTCTGAAATTCTCTCTATGCCTTTGAGAGACTCACCATTTAGATTCTGACTCCTAGAGTCCCTTAGTGGCCAGTCAACGTAAGTCAAGTTAGTAGCTACAGCCGTCAGGCACACGATTACACCGTGAACAGTGAAGTA
AAAAAATGTCCTTTCTTTGCTTTCTTCCTCTAATCACCATCCCAGAGGCATGAAAACCTAGAAAAAAGAAAGTAGCTGTATAGCTTTCATTTATGTGTAAGATTGGTGTTTTGAAATTTTAATAACAAAAAAGACTAGAAAGTTGCCTATCTCCCCAGAATGT
TTCTAGTGAATAGGAATAAGAAATAGGAAAAGTATATTCCATACAGTATAAGCAAATTTAAAAAATTGATTTTTTACCCTACTGAATTGAGACTTGCCAATAACCAGTTATACAAATAAAGAGGGTAGTCCTGGGAGCTGAGTGCTATGAAAAAAATACAGGA
AGAAAATGAAAGAGAAATTAATGGGAAGGAGGGAAGAAGACTACAAGCTGCTTTGCGTTGGGTAGGCAGGGAAGACCTATTTCATTCAATGAAATTTTCCTGAGTTTTCCTGTGTTCTAGCCATTATTCTGGGCTCTGAGGATACATAGTAAACAAAGCAGAT
GAAAAATACAGTCATCAGAATTTTCTTTGAAGGTGTGAGACAGGAATAATATGGAGGCAGTCAAACAAAGATATGAAGCAAAAAAAGGAGAAAAAATTAGAACAAAGTTCCTAAGAAAGGAGCAAGCTTAGTATGTTGGAGTTTTGGGGAAAAGTTCAGTGGC
TGCAACCTAATGACAGGAGAGAGTGGGAGAGGGTTGAAGGGGGCAGGCCAGGTGAGCTGAGCATGTGGACAAGTCAGAACTTCAGAGAGCATGCTAACAAGTGGTGATTTTATTCTGGTGCAGTGAAAAAC

The forward (DEL2_GYPEBAc_F3) and reverse (DEL2_GYPEBAs_R3) PCR primers are highlighted in yellow. The location of the 2 x BsrBI restriction enzyme sites are highlighted in green (#1 on forward strand; #2 on reverse strand); BsrBI is non-palindromic. The motifs recognised and cut but BsrBI are located in the GYPE-GYPB region sequence (red-bolded text). The putative DEL2 breakpoint is shown highlighted in blue in the GYPE-GYPB and GYPB-GYPA regions. An alignment with additional feature highlights is shown below.

C: DEL2 PCR strategy

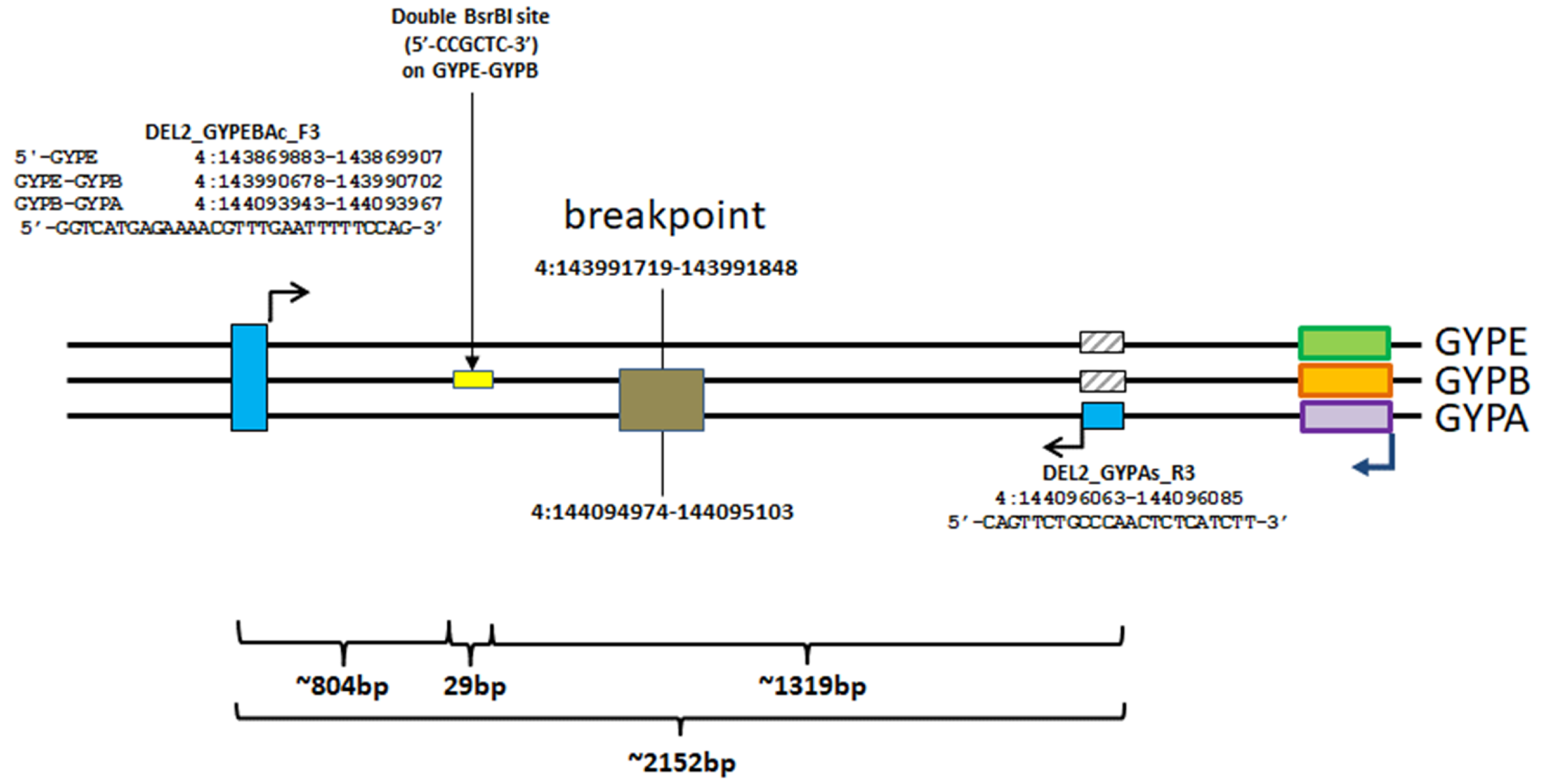

D: DEL2 RFLP strategy

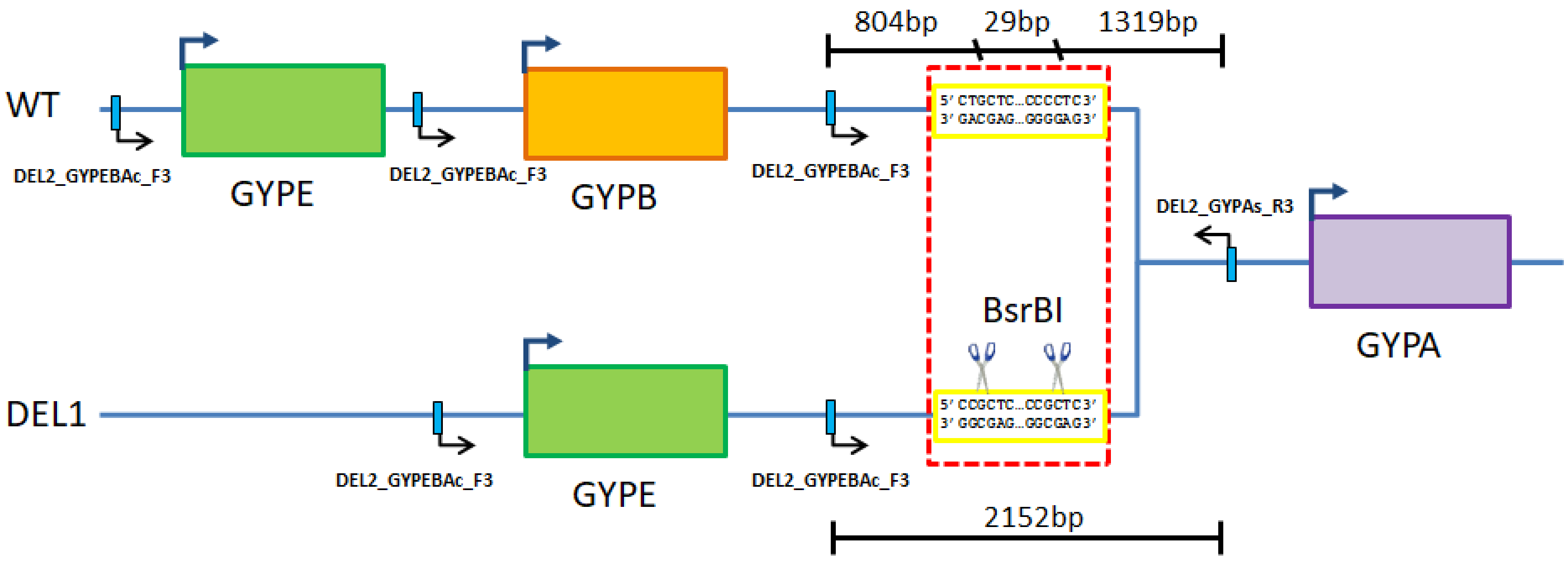

Supplementary Figure 3: Schematic representation of strategies for developing assays for the DEL2 GYP structural variant.
A: schematic of the alignment for the GYP SDUs showing the location of PCR primers, putative breakpoint and BsrBI restriction site.
The common forward PCR primer is shown towards the left of the sequence (DEL2_GYPEBAc_F3). The unique reverse PCR primer (DEL2_GYPAs_R3) is shown located 5' to GYPA

B: alternate schematic of the DEL2 RFLP assay showing a normal chromosome and the DEL2 chromosomes aligned. Genes and primers are indicated as well as the 2 x BsrBI restriction sites (that are located 29bp apart) and PCR-digestion fragment lengths.

Coordinates are given with respect to GRCh38.

DEL2 breakpoint region pileup of 2kb of the sequences above.

Sequences shown are between the forward and reverse PCR primers (the remaining sequences were removed for clarity in the alignment below). The alignment below was created using Clustal Omega (https://www.ebi.ac.uk/Tools/msa/clustalo/) and then manually finished and highlighted. All sequences are from the forward strand of the human reference genome.

The forward (DEL2_GYPEBAc_F3) and reverse (DEL2_GYPEBAs_R3) PCR primers are highlighted in grey with red-bolded text. Two primers used for sequencing the PCR product (DEL2_BP_seq_Rev1, and DEL2_BP_seq_Fwd) are also shown highlighted in grey with red-bolded text.
The 2 x BsrBI restriction enzyme sites are shown (#1 on forward strand; #2 on reverse strand); BsrBI is non-palindromic.
All differences between the 3 sequences are highlighted in green or yellow. The putative DEL2 breakpoint is shown highlighted in blue.

```
                                   DEL2_GYPEBAc_F3 →
DEL2:5'-GYPE        GGTCATAAGAAAACGTTTGAATTTTTCCGATCAGTCAGTCATTGTATTAGTTCATTCCCATGCTGCTATAAAGAAGTGCCTGAGACTGGGTAATTTATAAAGGAAAGAAGTTTAATTGAC   120
DEL2:GYPE-GYPB      GGTCATGAGAAAACGTTTGAATTTTTCCAGTCAGTCAGTCATTGTATTAGTTCATTCCCATGCTGCTATAAAGAAGTGCCTGAGACTGGGTAATTTATAAAGGAAAGAAGTTTAATTGAC   120
DEL2:GYPB-GYPA      GGTCATAAGAAAATGTTTGAATTTTTCCAGTCAGTCAGTCATTGTATTAGTTCATTCCCATGCTGCTATAAAGAAGTGCCTGAGACTGGGTAATTTATAAAGGAAAGAAGTTTAATTGAC   120
                    ****** ****** **************  ******************************************************************************************

DEL2:5'-GYPE        TCACAATTCTGCGTGGCTGAGAAGGTTTCAGGAAACTTACAATCGTGGCAGAAGGGGAAGCAAACACATCGTTTTTCACATGATGGCAAAAGTAAATGGGGAAGCCCCTTATAAAACCAT   240
DEL2:GYPE-GYPB      TCACAATTCTGCGTGGCTGAGAAGGTTTCAGGAAACTTACAATCGTGGCAGAAGGGGAAGCAAACACATCCTTTTTCACATGATGGCAAAAGTAAATGGGGAAGCCCCTTATAAAACCAT   240
DEL2:GYPB-GYPA      TCACAATTCTGCGTGGCTGAGAAGGTTTCAGGAAACTTACAATCGTGGCAGAAGGGGAAGCAAACACATCCTTTTTCACATGATGACAGAAGTAAATGGGGAAGAGCCTTATAAAACCAT   240
                    ********************************************************************** ************** ** ***************  **************

DEL2:5'-GYPE        CAGATCTCATGAGAATTTGCTCACTATCATGAAAATAGCATGGGGGAAACTGCCACAATGATTCAATTACCTCCCACTACATTCCTCCCACAACACATGGGGATTGTGGGAACTACAATT   360
DEL2:GYPE-GYPB      CAGATTTCATGAGAATTTGCTCACTATCATGAAAATAGCATGGGGGAAACTGCCACAATGATTCAATTACCTCCCACTACATTCCTCCCACAACACGTGGGGATTGTGGGAACTACAATT   360
DEL2:GYPB-GYPA      CAGATCTCATGAGAATTTGCTCACTATCATGAAAATAGCATGGGGGAAACAGCCACAATGATTCAATTACCTCCCACTACATTCCTCCCACAACACGTGGGGATTGTGGGAACTACAATT   360
                    ***** ******************************************** ********************************************* ***********************

DEL2:5'-GYPE        CAAGATGAGATTTGGGTGGGGACACAGCCAAACCACATCACTATGCCCCTGACCCCTCCCAAATCTCATGTCCTCACATTTCAAAACACAATCATGCCTTCCAAACAGTCCCCCAAAGTC   480
DEL2:GYPE-GYPB      CAAGAGGAGATTTGGGTGGGGACACAGCCAAACCACATCACTATGCCCCTGACCCCTCCCAAATCTCATGTCCTCACATTTCAAAACACAATCATGCCTTCCAAACAGTCCCCCAAAGTC   480
DEL2:GYPB-GYPA      CAAGATGAGATTTGGGTGGGGACACAGCCAAACCACATCACTATGCCCCTGACCCCTCGCAAATCTCATGTCCTCACATTTCAAAACACAATCATGCCTTCCAAACGGTCCCCCAAAGTC   480
                    ***** **************************************************** *********************************************** *************

DEL2:5'-GYPE        TAAACTCATTCCAGCATTAACTCAAAAGTCCAAGTCCAAAGTCTCATCTGAGACAAGGCAAGTCCCTTCCACCTATGAGCCTGTAAAATCAAAAGCAAGTTAGTTACTTCCTAGATACAA   600
DEL2:GYPE-GYPB      TAAACTCATTCCAGCATTAACTCAAAAGTCCAAGTCCAAAGTCTCATCTGAGACAAGGCAAGTCCCTTCCACCTATGAGCCTGTAAAATCAAAAGCAAGTTAGTTACTTCCTAGATACAA   600
DEL2:GYPB-GYPA      TTAACTCATTCTAGCATTAACTCAAAAGCCCAAGTCCAAAGTCTCATCTGAGACAAGGCAAGTACCTTCCACCTATGAGCCTGTAAAATCAAAAGCAAGTTAATTACTTCCTAGATACAA   600
                    * ********* **************** ********************************** ************************************** *****************

DEL2:5'-GYPE        TGAGGGTATAGGCAATGGGTAAATGCACCCATTCTAAATAGGATAAATTGGCCAAAAACAGGAGCTACAGGCCCATGCAAGTCTGAAATCCAGTGGAGCAGTAATTAAATCTTAAAGCAT   720
DEL2:GYPE-GYPB      TGAGGGTATAGGCAATGGGTAAATGCACCCACTCTAAATAGGAGCAATTGGCCAAAAACAGGGGCTACAGGCCCATGCAAGTCTGAAATCCAGTGGGGCAGTAATTAAATCTTAAAGCAC   720
DEL2:GYPB-GYPA      TGAGGGTATAGGCAATGGGTAAATGCACCCATTCTAAATAGGATAAATTGGCCAAAAACAGGAGCTACAGGCCCATGCAAGTCTGAAATCCAGTGGGGCAGTAATTAAATCTTAAAGCAC   720
                    ******************************* *********** ***************** ********************************* **********************

                                                                                           BsrBI restriction sites (CCGCTC) on FWD strand
                                                                                              #1                       #2
DEL2:5'-GYPE        CTTAATAATCTCTTTTGACTCCATGTCTCACATCCAGGTAATGCTGTTGCAAGAGGTGGGCTCCCACAGTCTTGGGAAGCTCAGCTCCTGTGGCTTTGCATGGTA-AACCCCCCCGCCTC--G 840
DEL2:GYPE-GYPB      CTTAATAATCTCTTTGGACTCCATGTCTCACATCCAGTTAATGCTGATGCAAGAGGTGGGCTCCCACAGTCTTGGGAAGCTCCGCTCCTGTGGCTTTGCATAGTACAACC----CCGCTCTCG 839
DEL2:GYPB-GYPA      CTTAATAATCTCTTTTAACTCCATGTCTCACATCCATGTAATGCTGATGCAAGAGGTGGGTTCCCACAGTCTTGGGAAGCTCTGCTCCTGTGGCTTTGCATGGTACAACC----CCCCTCTCG 839
                    ***************  *******************  ******** ************* ********************* ****************** *** ****    *  ***  *

DEL2:5'-GYPE        GCTGCTTTCACAGGCTGTCTGTTATCTAGTTCCAAAGTCACTTCTGCATTTTTAGGTATCCTTATAGCAGCACCCCACCTCTAGTACCAACTTACTGTATTAGTCTGTTCTCATGCTGTT   960
DEL2:GYPE-GYPB      GCTGCTTTCACAGGCTGTCTGTTATCCAGTTCCAAAGTCACTTCTGCATTTTTAGGTATCCTTATAGCAGCACCCCACCTCTAGTACCAACTTACTGTATTAGTCTGTTCTCATGCTGCT   959
DEL2:GYPB-GYPA      GCTGCTTTCACGGGC----TGTTATCCAATTCCAAAGTCACTTCTGCATTTTTAGGTATCCTTATAGCAGCACCCCACCTCTAGTACCAACTTACTGTATTAGTCTGTC-TCATGCTGCT   954
                    *********** ***    ******* * *******************************************************************************  ******** *

DEL2:5'-GYPE        ATAAAAAACTGCCCAAGACTGTGTAATTTATAAAGGAAAGAGGTTTTATTGATCTACAGTTTTGCATGGCTGGGAAGGTCTCAGGATACTTACAATCATGACCAAAGGGGAAACAAACA 1079
DEL2:GYPE-GYPB      ATAAAAAACTGCCCAAGACTGTGTAATTTATAAAGGAAAGAGGTTTAGTTGATCTACAGTTTTGCATGGCTTGGAAGGTCTTAGGATACTTACAATCATGACCAAAGGGGAAACAAACA 1078
DEL2:GYPB-GYPA      ATAAAAAACTTCCCAAGACTGTGTAATTTATAAA-----GAGGTTTAATTGATCTACAGTTTTGCATGGCTGGGAAGGTCTCAGGATACTTACAATCATGACCAAAGGGGAAACAAACA 1068
                    ********** * *********************     *******  *********************** ********* *************************************

DEL2:5'-GYPE        CATCTTTCTTACATAGTGGCAGGAAGGAGAAGAATGAGAGCTGAGTGAAGGGGGAAGCTCCTTTATAAAACTATCAGATTATGTGAGAATTTACTCACTATCATGAGAATAGCACAGGGG   1199
DEL2:GYPE-GYPB      CATCTTTCTTACATAGTGGCAGGAAGGAGAAGAATGAGAGCTGAGTGAAGGGGGAAGCTCCTTTATAAAACTATCAGATTATGTGAGAATTTACTCACTATCATGAGAATAGCACAGGGG   1198
DEL2:GYPB-GYPA      CATCTTTCTTACATAGTGGCAGGAAGGAGAAGAATGAGAGCTGAGTGAAGGGGGAAGCTCCTTTATAAAACTATCAGATTATGTGAGAATTTATTCACTCTCATGAGAATAGCATAGGGG   1188
                    ********************************************************************************************* ***** ************** *****

DEL2:5'-GYPE        AAACCACCGCAATGATTCAAGTACCTCCCACTGGGTTCCTCCCATGACATGTGGGGATTATTGGAACTACAATTCAAGATGAGATTTGAGTGGGAACACAGCCAAACCATATCAGTCATT   1319
DEL2:GYPE-GYPB      AAACCACCGCAATGATTCAAGTACCTCCCACTGGGTTCCTCCCATGACATGTGGGGATTATTGGAACTACAATTCAAGATGAGATTTGAGTGGGAACACAGTCAAACCATATTAGTCATT   1318
DEL2:GYPB-GYPA      AAACCACCGCAATGATTCAAGTACCTCCCACTGGGTTCCTCCCATGACACGTGGGGATTATTGGAACTACAATTCAAGATGAGATTTGAGTGGGAACACAGCCAAACCATATCAGTCATT   1308
                    ************************************************* *************************************************** ********** *******

                                                    ← DEL2_BP_seq_Rev1   ((5'-CTATGGGTCCCTCTCTGTGGA -3')
DEL2:5'-GYPE        CCACATATTGAGTGATTTCTCTCTTCTATTCAGTATTCTTCCACAGAGAGGGACCCATAGTCATTACCTTCAAGGAACCTAAATCCTGGTGTTTTATGGTTACGGGTGGCATATAATAT   1439
DEL2:GYPE-GYPB      CCACATATTGAGTGATTTCTCTCTTCTATTCACTATTCTTCCACAGAGAGGGACCCATAGTCATTACCTTCAAGGAACCTAAATCCTGGCATCTTTATTGTTATGGGTGGTGTATAATAT   1438
DEL2:GYPB-GYPA      CCACATATTGAGTGATTTCTCTCTTCTATTCACTATTCTTCCACAGAGAGGGACCTGTAGTCATTACCTTCAAGGAACCTAAATCCTGGTGTCTTTATGGTTATGGGTGGCATATAATAT   1428
                    ******************************** **********************  ********************************  * ***** **** ******  ********

DEL2:5'-GYPE        ACAGGTAAGCCAATGTTACAGGATGTTGTGGAACTATTCCTATTCTTCCATAAGTAGCCACTAGGACCACGGCTGAAACCAAGAGGCACTGATTACCACCTCACTCTAAAGAGAATTACT   1559
DEL2:GYPE-GYPB      ACAGGTAAGCCAATGTTACAGGATGTTGTGAGACTATTCCTATCCTTCCATAATTAGCCACTAGGACCACAGCTGAAACCAAGAGGCACTGATTACCCCCTCACTCTAAAGAGAATTACT   1558
DEL2:GYPB-GYPA      ACAGGTAAGCCAATGTTACAGGATGTTGTGAGACTATTCCTGTTCTTCCATAATTAGCCACTAGGACCACGGCTGAAACCAAGAGGCACTGATTACCGCCTCACTCTAAAGAGAATTACT   1548
                    **** *************************  ********* * ********* **************** ************************** **********************

DEL2:5'-GYPE        GAGTTTGTGTTCACTGCTGGGGCCAGATCTCTTTGCAGGGCTATGTTAGGCTTTACACAATGTGACCCTCTAGTCTGTACAAGGAGAGGAATGCAGAGGGAAAGCGATTCACTGGAGTCT   1679
DEL2:GYPE-GYPB      GAGCTTGTGCTCACTGCGGGGGCCAGATCTTTTTGCAGGGCTATGTTAGGCTTTACACAATGTGACCCTCTAGTCTGTACAAGGAGAGGAATGCAGAGGGGAAGAGATTCACTGGAGTCT   1678
DEL2:GYPB-GYPA      GAGTTTGTGCTCACTGCTGGGGCCAGATCTCTTTGCAGGGCTATGTTTGGCTTTACACAATGTGACCCTCTAGTCTGTACAAGGAGAGGAATGCAGAGGGAAAGAGATTCACTGGAGTCT   1668
                    *** ***** ******* ************ **************** **************************************************** *** ***************

DEL2:5'-GYPE        CTCGTCAGTGACCTGGTGGTGAATATATGGTAATGGGTAAATCCCACTTCACCTAATGAAGAAAAGGAGAAGGATGCACTTCATTCTTAGAATCTAAGCAGGAACAGGAAAGGGAAAATC   1799
DEL2:GYPE-GYPB      CTCGTCAGTGACCTGGTGGTGAATATATGGTAATGGGTAAATCCCACTTCACCTGATGAAGAAAAGGAGAAGGATGCACTTCATTCTTAGAATCTAAGCAGGAACGGGAAAGGGAAAATT   1798
DEL2:GYPB-GYPA      CTCGTCAGTGACCTGGTGGTGAATATATGGTAATGGGTAAATCCCACTTCACCTGATGAAGAAAAGGAGAAGGATGCACTTCATTCTTAGAATCTAAGCAGGAACAGGAAAGGGAAAATC   1788
                    ****************************************************** ************************************************** *************

DEL2:5'-GYPE        TAGGAGAAAAGCTTACAAAGACTGTAAATGCCTTCAAGAAGGTGAGAGAAACTAATATTTGGTTTACTGTCAGCAGTCTGTTCGGGCGGCATTTTGCCAATATA-GCCCTAT--------   1910
DEL2:GYPE-GYPB      AGGAG-AAAAGCTTACAAAGACTGTAAATGCCTTCAAGAAGGTGAGAGAAACTAATATTTGGTTTACTGTCAGCAGGCTGTTCGGGCGGCATTTTGCCAATATA-GCCCTATCCCTACGT   1916
DEL2:GYPB-GYPA      TAGGA-GAAAA-GCTTACAAAGACTAAATGTCTTCAAGAAGGTGAGAGAAACTAATATTTGGTTTACTGTCAGCAGGCTGTTTGGGCGGCATTTTGCCAATATTAGCCCTATCCCTACGT   1906
                      *    ***       * * *  ****** ********************************************* ***** ********************  *******

DEL2:5'-GYPE        CC-TAAGGGAAAGGGAATTGTGGTTGGACAACCGAGTTCTGAGAAAGGCAGTGATTGAGCAGCTGAATTCCATCCATTTGGTGGGTAAGGACCTCAGAGTATTCAAAGTATCAAATGGAA   2029
DEL2:GYPE-GYPB      GAGTA-GGGGAAGGGAATTGTGGTTGGACAACAGAGTTCTGAGAAAGGCAGTGATTGAGCAGCTGAACTCCATCCATTTGGTGGGTAAGGACCCCAGAGTATTCAAAGTATCAAATGGAA   2035
DEL2:GYPB-GYPA      GAGTAGGGGAAAGGGAATTGTGGTTGGACAACCGAGTTCTGAGAAAGGCAGTGATTGAGCAGCTGAACTCCATCCATTTGGTGGGTAAGGACCCCAGAGTATTCAAAGGATCAAATGGAA   2026
                       ** *** ********************** ********************************** ************************* ************** ***********

DEL2:5'-GYPE        TGTGACTGAAGAGTTATCTTGCTGATCTTACCCAGGAGGACTGTCCCCTAGGCAAGGGGACACCAAACACAATGAGGCATGGGATAAGGATTTCGTGATGAGAATCGGGCAGAACTG   2146
DEL2:GYPE-GYPB      TGTGACTGAAGTGTTATCTTGCTGATGTTACCCAGGAAAACTGTCCCCTAGGCAAGGGGACACCAAACACAAAGAGGCACGGGATAAGGATTTCGTGACGAGAATTGGGCAGAACTG   2152
DEL2:GYPB-GYPA      TGTGACTGAAGAGTTATCTTGCCGATCTTACCCAGGAAGACTGTCCCCTAGGCAAGGGGACACCAAACACAAAGAGGCATGGGATAAGGATTTCAAGATGAGAGTTGGGCAGAACTG   2143
                    *********** ********* *** **********  **************************************** **************  ** **** * ***********
                                                                                                              ← DEL2_GYPEBAs_R3
                                                                                                            (5'-CAGTTCTGCCCAACTCTCATCTT-3')
```

Amuzu *et al.*, Supplementary File 4: DEL1 - Sanger Sequence Pile-Ups

Sanger Sequence alignments for DEL1 PCR products.
Coordinates are with respect to GRCH37 and GRCh38 and the forward strand of the human reference sequence.

GYP target	Primers	Sequence (5'-3')	Dir	GC (%)	T_m (°C)	GYP region	GRCh37 locations	GRCh38 locations
DEL1	GYP_DEL1_F10	GGACTGCCGCATGTTCAG	Fwd	61	53	GYPE-GYPB	4:144833378-144833395	4:143912225-143912242
DEL1	GYP_DEL1_R2B5	CTCTGGTAGCCCTCCTCAAG	Rev	60	56	GYPE-GYPB	4:144835652-144835671	4:143914499-143914518
						GYPB-GYPA	4:144945890-144945909	4:144024737-144024756
						GYPA-3'	4:145067236-145067255	4:144146083-144146102

Table 1: Primer sequences used for DEL1 PCR and Sanger Sequencing

**Putative Breakpoint:**	**GRCh37**	**GRCh38**	
GYPE-GYPB:	4:144835169-144835279	4:143914020-143914126	~8.5kb 3' to the GYPE ATG start site (GRCH37:4:144826660 and GRCH38:4:143905507).
GYPB-GYPA:	4:144945407-144945517	4:144024258-144024364	~4.9kb 3' to the GYPB ATG start site (GRCH37:4:144940440 and GRCH38: 4:144019287)

AciI decriminating sites (common to both the normal and hybrid products are highlighted in Magenta): note that this enzyme is strand-specific and not palindromic. Recognition site: 3'-CCGC-5' with the complementary strand 5'-GCGG-3'

		**GRCH37**	**GRCH38**	**site**
**AciI** decriminating sites (magenta):	5'-GYPE:	4:144835375-144835378	4:143914222-143914225	CUT (GCGG)
This is labelled 3' to the	GYPE-GYPB:	4:144945613-144945616	4:144024460-144024463	NOT-CUT (GTGG)
breakpoint region	GYPB-GYPA:	4:145066959-145066962	4:144145806-144145809	NOT-CUT (GTGA)

DEL1 alignments of Sanger sequence data with the GYPE-GYPB and GYPB-GYPA reference sequences across the PCR region (note that the GYPB-GYPA sequence begins just before the start of the homologous sequence with GYPE-GYPB sequence [vertical arrow])

PCR/Sequencing primers are indicated and highlighted in grey with red text

Differences between sequences:

- Yellow highlights the GYPE-GYPB reference nucleotides;
- blue highlights the GYPB-GYPA reference sequence;
- magenta highlights the AciI restrictions sites;
- green highlights the putative breakpoint region.

Marked-up bases (bases that are different between the reference sequences):

- X; discriminates between GYPE-GYPB and GYPB-GYPA regions
- B; non-discriminatory polymorphism
- Z; 5' region of the putative breakpoint. Complex set of polymorphisms including 2 di-nucleotide repeat motifs. The TA motif may also show its own length polymorphism (samples marked **). The key discriminatory polymorphisms between GYPE-GYPB and the GYPB-GYPA regions is the 8 base CA_4 repeat. This forms the 5' end of the breakpoint region.
- ZZ; 3' end of the breakpoint. This is the first polymorphic base after the CA4 motif that shows a clear GYPE-GYPB or GYPB-GYPA origin. The breakpoint region is therefore within this 111 base region.

Samples (see main text Table 2):

- GM19140; 1000G/HapMap cell line - normal
- GX0387; Ghanaian with normal PCR for DEL1/DEL2/Dup4
- GX0540; Ghanaian with normal PCR for DEL1/DEL2/Dup4
- GX0531; Ghanaian with normal PCR for DEL1/DEL2/Dup4

- HG02464; 1000G/HapMap cell line – DEL1 homozygote
- HG02545; 1000G/HapMap cell line – DEL1 homozygote
- GX0258; Ghanaian DEL1 homozygote & normal PCR for DEL2/Dup4
- GX0458; Ghanaian DEL1 homozygote & normal PCR for DEL2/Dup4
- GX0537; Ghanaian DEL1 homozygote & normal PCR for DEL2/Dup4

- GX0300; Ghanaian DEL1/DEL2 heterozygote & normal PCR for Dup4

All sequences look like the GYPE-GYPB region from the specific GYP_DEL1_F10 forward primer, as expected, through to the 8-base deletion (CACACACA) (~1670 and 1900 bases from sequence start). After the green highlighted putative breakpoint, the normal samples still match the GYPE-GYPB region while the other samples identified as DEL1 match the GYPB-GYPA region, through to the 'common' GYP_DEL1_R2B5 reverse primer.

```
                                                            GYP_DEL1_F10 (fwd) ➔
GYPB-GYPA             ------------------------------------------------------------        0
GYPE-GYPB             CTCCGCCGCTGCCTGTCCGGAGCCTGGGGTCGCCCGCAGGGACTGCCGCATGTTCAGGGC        60


                                    AciI                                     AciI
GYPB-GYPA             ------------------------------------------------------------        0
GYPE-GYPB             GCTAAACGCGCCGGCCGCCGCTCAGTCGCTGGTCACTTCCTTCCCGGAAGTCGGCCCGCT        120
>GM19140_D1_F10                                                    GGAAGTCGGCCCGCT
>GX0387-C_D1_F10                               TCGCTGGTCACTTCCTTCCCGGAAGTCGGCCCGCT
>GX0531-C_D1_F10                               TCGCTGGTCACTTCCTTCCCGGAAGTCGGCCCGCT
>GX0540-C_D1_F10                               TCGCTGGTCACTTCCTTCCCGGAAGTCGGCCCGCT
>HG02464_D1_F10                                                    GGAAGTCGGCCCGCT
>HG02545_D1_F10                               GTCGCTGGTCACTTCCTTCCCGGAAGTCGGCCCGCT
>GX0258-C_D1_F10                                  CTGGTCACTTCCTTCCCGGAAGTCGGCCCGCT
>GX0458-C_D1_F10                                CGCTGGTCACTTCCTTCCCGGAAGTCGGCCCGCT
>GX0537-C_D1_F10                                CGCTGGTCACTTCCTTCCCGGAAGTCGGCCCGCT
>GX0300-C_D1_F10                                 GCTGGTCACTTCCTTCCCGGAAGTCGGCCCGCT
                                                                   ***************


                                                 Start of homologous region ↓
GYPB-GYPA             -----------------------------------------------------GCATTCT        7
GYPE-GYPB             CTGCGACGCTGCTCGGGGACCCCTTGAGGAAAGCCCAGCGACGCCGGGCCAGCGCATTCT        240
>GM19140_D1_F10       CTGCGACGCTGCTCGGGGACCCCTTGAGGAAAGCCCAGCGACGCCGGGCCAGCGCATTCT
>GX0387-C_D1_F10      CTGCGACGCTGCTCGGGGACCCCTTGAGGAAAGCCCAGCGACGCCGGGCCAGCGCATTCT
>GX0531-C_D1_F10      CTGCGACGCTGCTCGGGGACCCCTTGAGGAAAGCCCAGCGACGCCGGGCCAGCGCATTCT
>GX0540-C_D1_F10      CTGCGACGCTGCTCGGGGACCCCTTGAGGAAAGCCCAGCGACGCCGGGCCAGCGCATTCT
>HG02464_D1_F10       CTGCGACGCTGCTCGGGGACGCCTTGAGGAAAGCCCAGCGACGCCGGGCCAGCGCATTCT
>HG02545_D1_F10       CTGCGACGCTGCTCGGGGACGCCTTGAGGAAAGCCCAGCGACGCCGGGCCAGCGCATTCT
>GX0258-C_D1_F10      CTGCGACGCTGCTCGGGGACGCCTTGAGGAAAGCCCAGCGACGCCGGGCCAGCGCATTCT
>GX0458-C_D1_F10      CTGCGACGCTGCTCGGGGACGCCTTGAGGAAAGCCCAGCGACGCCGGGCCAGCGCATTCT
>GX0537-C_D1_F10      CTGCGACGCTGCTCGGGGACGCCTTGAGGAAAGCCCAGCGACGCCGGGCCAGCGCATTCT
>GX0300-C_D1_F10      CTGCGACGCTGCTCGGGGACGCCTTGAGGAAAGCCCAGCGACGCCGGGCCAGCGCATTCT
                      ************************************************************


                             XX                             X       X
GYPB-GYPA             TATTTTAAATATGAGCAAACTATGGAATGGAAAAGAGACTATGAGTGTATTACTTGATTT        67
GYPE-GYPB             TATTTTATGTATGAGCAAACTATGGAATGGAAAAGAGATTATGAGT-TATTACTTGATTT        299
>GM19140_D1_F10       TATTTTATGTATGAACAAACTATGGAATGGAAAAGAGATTATGAGT-TATTACTTGATTT
>GX0387-C_D1_F10      TATTTTATGTATGAGCAAACTATGGAATGGAAAAGAGATTATGAGT-TATTACTTGATTT
>GX0531-C_D1_F10      TATTTTATGTATGAGCAAACTATGGAATGGAAAAGAGATTATGAGT-TATTACTTGATTT
>GX0540-C_D1_F10      TATTTTATGTATGAGCAAACTATGGAATGGAAAAGAGATTATGAGT-TATTACTTGATTT
>HG02464_D1_F10       TATTTTATGTATGAGCAAACTATGGAATGGAAAAGAGATTATGAGT-TATTACTTGATTT
>HG02545_D1_F10       TATTTTATGTATGAGCAAACTATGGAATGGAAAAGAGATTATGAGT-TATTACTTGATTT
>GX0258-C_D1_F10      TATTTTATGTATGAGCAAACTATGGAATGGAAAAGAGATTATGAGT-TATTACTTGATTT
>GX0458-C_D1_F10      TATTTTATGTATGAGCAAACTATGGAATGGAAAAGAGATTATGAGT-TATTACTTGATTT
>GX0537-C_D1_F10      TATTTTATGTATGAGCAAACTATGGAATGGAAAAGAGATTATGAGT-TATTACTTGATTT
>GX0300-C_D1_F10      TATTTTATGTATGAGCAAACTATGGAATGGAAAAGAGATTATGAGT-TATTACTTGATTT
                      *******  ***************************** ******* *************


                                                            X  X
GYPB-GYPA             GATTTGCCCTAAGTGTTTTTTAAACGTATGGATTTAGCTGCTAACATTTAAAAAGAAGGT        127
GYPE-GYPB             GATTTGCCCTAAGTGTTTTTTAAACGTATGGATTTAGC-GCCAACATTTAAAAAGAAGGT        358
>GM19140_D1_F10       GATTTGCCCTAAGTGTTTTTTAAACGTATGGATTTAGC-GCCAACATTTAAAAAGAAGGT
>GX0387-C_D1_F10      GATTTGCCCTAAGTGTTTTTTAAACGTATGGATTTAGC-GCCAACATTTAAAAAGAAGGT
>GX0531-C_D1_F10      GATTTGCCCTAAGTGTTTTTTAAACGTATGGATTTAGC-GCCAACATTTAAAAAGAAGGT
>GX0540-C_D1_F10      GATTTGCCCTAAGTGTTTTTTAAACGTATGGATTTAGC-GCCAACATTTAAAAAGAAGGT
>HG02464_D1_F10       GATTTGCCCTAAGTGTTTTTTAAACGTATGGATTTAGC-GCCAACATTTAAAAAGAAGGT
>HG02545_D1_F10       GATTTGCCCTAAGTGTTTTTTAAACGTATGGATTTAGC-GCCAACATTTAAAAAGAAGGT
>GX0258-C_D1_F10      GATTTGCCCTAAGTGTTTTTTAAACGTATGGATTTAGC-GCCAACATTTAAAAAGAAGGT
>GX0458-C_D1_F10      GATTTGCCCTAAGTGTTTTTTAAACGTATGGATTTAGC-GCCAACATTTAAAAAGAAGGT
>GX0537-C_D1_F10      GATTTGCCCTAAGTGTTTTTTAAACGTATGGATTTAGC-GCCAACATTTAAAAAGAAGGT
>GX0300-C_D1_F10      GATTTGCCCTAAGTGTTTTTTAAACGTATGGATTTAGC-GCCAACATTTAAAAAGAAGGT
```

```
                  ************************************** ** ******************

                                                    B              X    X
GYPB-GYPA         ATATTTTAATTAAAAATCAGGAATTGATTTAATTAAAAATTTGGCTCCTGTTACCGGAAG        187
GYPE-GYPB         ATATTTTAATTAAAAATCAGGAATTGATTTAATTTAAAATTTGGCTCCTCTTACAGGAAG        418
>GM19140_D1_F10   ATATTTTAATTAAAAATCAGGAATTGATTTAATTTAAAATTTGGCTCCTCTTACAGGAAG
>GX0387-C_D1_F10  ATATTTTAATTAAAAATCAGGAATTGATTTAATTTAAAATTTGGCTCCTCTTACAGGAAG
>GX0531-C_D1_F10  ATATTTTAATTAAAAATCAGGAATTGATTTAATTTAAAATTTGGCTCCTCTTACAGGAAG
>GX0540-C_D1_F10  ATATTTTAATTAAAAATCAGGAATTGATTTAATTTAAAATTTGGCTCCTCTTACAGGAAG
>HG02464_D1_F10   ATATTTTAATTAAAAATCAGGAATTGATTTAATTAAAAATTTGGCTCCTCTTACAGGAAG
>HG02545_D1_F10   ATATTTTAATTAAAAATCAGGAATTGATTTAATTAAAAATTTGGCTCCTCTTACAGGAAG
>GX0258-C_D1_F10  ATATTTTAATTAAAAATCAGGAATTGATTTAATTAAAAATTTGGCTCCTCTTACAGGAAG
>GX0458-C_D1_F10  ATATTTTAATTAAAAATCAGGAATTGATTTAATTAAAAATTTGGCTCCTCTTACAGGAAG
>GX0537-C_D1_F10  ATATTTTAATTAAAAATCAGGAATTGATTTAATTAAAAATTTGGCTCCTCTTACAGGAAG
>GX0300-C_D1_F10  ATATTTTAATTAAAAATCAGGAATTGATTTAATTAAAAATTTGGCTCCTCTTACAGGAAG
                  ********************************** ************** **** *****


                                   X                      X
GYPB-GYPA         ATCTGGCAAGAGTTAGCATGCAGTTCTACATTGTGACAATCCATGAGAGCTAAGGACTGG        247
GYPE-GYPB         ATCTGGCAAGAGTTAGCGTGCAGTTCTACATTGTGACAATTCATGAGAGCTAAGGACTGG        478
>GM19140_D1_F10   ATCTGGCAAGAGTTAGCGTGCAGTTCTACATTGTGACAATTCATGAGAGCTAAGGACTGG
>GX0387-C_D1_F10  ATCTGGCAAGAGTTAGCGTGCAGTTCTACATTGTGACAATTCATGAGAGCTAAGGACTGG
>GX0531-C_D1_F10  ATCTGGCAAGAGTTAGCGTGCAGTTCTACATTGTGACAATTCATGAGAGCTAAGGACTGG
>GX0540-C_D1_F10  ATCTGGCAAGAGTTAGCGTGCAGTTCTACATTGTGACAATTCATGAGAGCTAAGGACTGG
>HG02464_D1_F10   ATCTGGCAAGAGTTAGCGTGCAGTTCTACATTGTGACAATTCATGAGAGCTAAGGACTGG
>HG02545_D1_F10   ATCTGGCAAGAGTTAGCGTGCAGTTCTACATTGTGACAATTCATGAGAGCTAAGGACTGG
>GX0258-C_D1_F10  ATCTGGCAAGAGTTAGCGTGCAGTTCTACATTGTGACAATTCATGAGAGCTAAGGACTGG
>GX0458-C_D1_F10  ATCTGGCAAGAGTTAGCGTGCAGTTCTACATTGTGACAATTCATGAGAGCTAAGGACTGG
>GX0537-C_D1_F10  ATCTGGCAAGAGTTAGCGTGCAGTTCTACATTGTGACAATTCATGAGAGCTAAGGACTGG
>GX0300-C_D1_F10  ATCTGGCAAGAGTTAGCGTGCAGTTCTACATTGTGACAATTCATGAGAGCTAAGGACTGG
                  ***************** ********************** *******************


                                                                  X
GYPB-GYPA         CTGTCTCTTGGGACAAATAGGCTTTCTTGAGTCTGGTAAGTGCTCCACCTGATGCTTGCT        307
GYPE-GYPB         CTGTCTCTTGGGACAAATAGGCTTTCTTGAGTCTGGTAAGTGCTCCACTTGATGCTTGCT        538
>GM19140_D1_F10   CTGTCTCTTGGGACAAATAGGCTTTCTTGAGTCTGGTAAGTGCTCCACTTGATGCTTGCT
>GX0387-C_D1_F10  CTGTCTCTTGGGACAAATAGGCTTTCTTGAGTCTGGTAAGTGCTCCACTTGATGCTTGCT
>GX0531-C_D1_F10  CTGTCTCTTGGGACAAATAGGCTTTCTTGAGTCTGGTAAGTGCTCCACTTGATGCTTGCT
>GX0540-C_D1_F10  CTGTCTCTTGGGACAAATAGGCTTTCTTGAGTCTGGTAAGTGCTCCACTTGATGCTTGCT
>HG02464_D1_F10   CTGTCTCTTGGGACAAATAGGCTTTCTTGAGTCTGGTAAGTGCTCCACTTGATGCTTGCT
>HG02545_D1_F10   CTGTCTCTTGGGACAAATAGGCTTTCTTGAGTCTGGTAAGTGCTCCACTTGATGCTTGCT
>GX0258-C_D1_F10  CTGTCTCTTGGGACAAATAGGCTTTCTTGAGTCTGGTAAGTGCTCCACTTGATGCTTGCT
>GX0458-C_D1_F10  CTGTCTCTTGGGACAAATAGGCTTTCTTGAGTCTGGTAAGTGCTCCACTTGATGCTTGCT
>GX0537-C_D1_F10  CTGTCTCTTGGGACAAATAGGCTTTCTTGAGTCTGGTAAGTGCTCCACTTGATGCTTGCT
>GX0300-C_D1_F10  CTGTCTCTTGGGACAAATAGGCTTTCTTGAGTCTGGTAAGTGCTCCACTTGATGCTTGCT
                  ************************************************ ***********




GYPB-GYPA         TCCATTTCTTAGATTAGGTTCTACTGTGGTAAGGCCTCAAAATCTCAGTGTCTTGCAGCA        367
GYPE-GYPB         TCCATTTCTTAGATTAGGTTCTACTGTGGTAAGGCCTCAAAATCTCAGTGTCTTGCAGCA        598
>GM19140_D1_F10   TCCATTTCTTAGATTAGGTTCTACTGTGGTAAGGCCTCAAAATCTCAGTGTCTTGCAGCA
>GX0387-C_D1_F10  TCCATTTCTTAGATTAGGTTCTACTGTGGTAAGGCCTCAAAATCTCAGTGTCTTGCAGCA
>GX0531-C_D1_F10  TCCATTTCTTAGATTAGGTTCTACTGTGGTAAGGCCTCAAAATCTCAGTGTCTTGCAGCA
>GX0540-C_D1_F10  TCCATTTCTTAGATTAGGTTCTACTGTGGTAAGGCCTCAAAATCTCAGTGTCTTGCAGCA
>HG02464_D1_F10   TCCATTTCTTAGATTAGGTTCTACTGTGGTAAGGCCTCAAAATCTCAGTGTCTTGCAGCA
>HG02545_D1_F10   TCCATTTCTTAGATTAGGTTCTACTGTGGTAAGGCCTCAAAATCTCAGTGTCTTGCAGCA
>GX0258-C_D1_F10  TCCATTTCTTAGATTAGGTTCTACTGTGGTAAGGCCTCAAAATCTCAGTGTCTTGCAGCA
>GX0458-C_D1_F10  TCCATTTCTTAGATTAGGTTCTACTGTGGTAAGGCCTCAAAATCTCAGTGTCTTGCAGCA
>GX0537-C_D1_F10  TCCATTTCTTAGATTAGGTTCTACTGTGGTAAGGCCTCAAAATCTCAGTGTCTTGCAGCA
>GX0300-C_D1_F10  TCCATTTCTTAGATTAGGTTCTACTGTGGTAAGGCCTCAAAATCTCAGTGTCTTGCAGCA
                  ************************************************************
```

```
GYPB-GYPA           ACACTGGTTCATTTCCTGCTCACATTACATTTGGTCTATGACGGTGACAAGTTCCAGGAT        437
GYPE-GYPB           ACACTGGTTCATTTCCTGCTCACATTACATTTGGTCTATGACGGTGACAAGTTCCAGGAT        658
>GM19140_D1_F10     ACACTGGTTCATTTCCTGCTCACATTACATTTGGTCTATGACGGTGACAAGTTCCAGGAT
>GX0387-C_D1_F10    ACACTGGTTCATTTCCTGCTCACATTACATTTGGTCTATGACGGTGACAAGTTCCAGGAT
>GX0531-C_D1_F10    ACACTGGTTCATTTCCTGCTCACATTACATTTGGTCTATGACGGTGACAAGTTCCAGGAT
>GX0540-C_D1_F10    ACACTGGTTCATTTCCTGCTCACATTACATTTGGTCTATGACGGTGACAAGTTTCAGGAT
>HG02464_D1_F10     ACACTGGTTCATTTCCTGCTCACATTACATTTGGTCTATGACGGTGACAAGTTCCAGGAT
>HG02545_D1_F10     ACACTGGTTCATTTCCTGCTCACATTACATTTGGTCTATGACGGTGACAAGTTCCAGGAT
>GX0258-C_D1_F10    ACACTGGTTCATTTCCTGCTCACATTACATTTGGTCTATGACGGTGACAAGTTCCAGGAT
>GX0458-C_D1_F10    ACACTGGTTCATTTCCTGCTCACATTACATTTGGTCTATGACGGTGACAAGTTCCAGGAT
>GX0537-C_D1_F10    ACACTGGTTCATTTCCTGCTCACATTACATTTGGTCTATGACGGTGACAAGTTCCAGGAT
>GX0300-C_D1_F10    ACACTGGTTCATTTCCTGCTCACATTACATTTGGTCTATGACGGTGACAAGTTCCAGGAT
                    ************************************************************


GYPB-GYPA           CCAGACTGAAGGAGCCACCCCCATTTGGAATATGCTGTTCTTGTTGCAAACTGGGGGAAA        497
GYPE-GYPB           CCAGACTGAAGGAGCCACCCCCATTTGGAATATGCTGTTCTTGTTGCAAACTGGGGGAAA        718
>GM19140_D1_F10     CCAGACTGAAGGAGCCACCCCCATTTGGAATATGCTGTTCTTGTTGCAAACTGGGGGAAA
>GX0387-C_D1_F10    CCAGACTGAAGGAGCCACCCCCATTTGGAATATGCTGTTCTTGTTGCAAACTGGGGGAAA
>GX0531-C_D1_F10    CCAGACTGAAGGAGCCACCCCCATTTGGAATATGCTGTTCTTGTTGCAAACTGGGGGAAA
>GX0540-C_D1_F10    CCAGACTGAAGGAGCCACCCCCATTTGGAATATGCTGTTCTTGTTGCAAACTGGGGGAAA
>HG02464_D1_F10     CCAGACTGAAGGAGCCACCCCCATTTGGAATATGCTGTTCTTGTTGCAAACTGGGGGAAA
>HG02545_D1_F10     CCAGACTGAAGGAGCCACCCCCATTTGGAATATGCTGTTCTTGTTGCAAACTGGGGGAAA
>GX0258-C_D1_F10    CCAGACTGAAGGAGCCACCCCCATTTGGAATATGCTGTTCTTGTTGCAAACTGGGGGAAA
>GX0458-C_D1_F10    CCAGACTGAAGGAGCCACCCCCATTTGGAATATGCTGTTCTTGTTGCAAACTGGGGGAAA
>GX0537-C_D1_F10    CCAGACTGAAGGAGCCACCCCCATTTGGAATATGCTGTTCTTGTTGCAAACTGGGGGAAA
>GX0300-C_D1_F10    CCAGACTGAAGGAGCCACCCCCATTTGGAATATGCTGTTCTTGTTGCAAACTGGGGGAAA
                    ************************************************************


                           X                                 X  X X X --B---
GYPB-GYPA           AAGGCCAAATAAGGAATGGCTTTTAATGCTTCCTCTCAGACATGGTATGTGTTATTTTTT        557
GYPE-GYPB           AAGGCCAGATAAGGAATGGCTTTTAATGCTTCCTCTCAGAC-TGTGTTATGG----TTTT        773
>GM19140_D1_F10     AAGGCCAGATAAGGAATGGCTTTTAATGCTTCCTCTCAGAC-TGTGTTATGG----TTTT
>GX0387-C_D1_F10    AAGGCCAGATAAGGAATGGCTTTTAATGCTTCCTCTCAGAC-TGTGTTATGG----TTTT
>GX0531-C_D1_F10    AAGGCCAGATAAGGAATGGCTTTTAATGCTTCCTCTCAGAC-TGTGTTATGG----TTTT
>GX0540-C_D1_F10    AAGGCCAGATAAGGAATGGCTTTTAATGCTTCCTCTCAGAC-TGTGTTATGG----TTTT
>HG02464_D1_F10     AAGGCCAGATAAGGAATGGCTTTTAATGCTTCCTCTCAGAC-TGTGTTAT----TTTTTT
>HG02545_D1_F10     AAGGCCAGATAAGGAATGGCTTTTAATGCTTCCTCTCAGAC-TGTGTTAT----TTTTTT
>GX0258-C_D1_F10    AAGGCCAGATAAGGAATGGCTTTTAATGCTTCCTCTCAGAC-TGTGTTAT----TTTTTT
>GX0458-C_D1_F10    AAGGCCAGATAAGGAATGGCTTTTAATGCTTCCTCTCAGAC-TGTGTTAT----TTTTTT
>GX0537-C_D1_F10    AAGGCCAGATAAGGAATGGCTTTTAATGCTTCCTCTCAGAC-TGTGTTAT----TTTTTT
>GX0300-C_D1_F10    AAGGCCAGATAAGGAATGGCTTTTAATGCTTCCTCTCAGAC-TGTGTTAT----TTTTTT
                    ******* *********************************   **    *     ****


                           X                                          X
GYPB-GYPA           TTTCTCATATTCTATTAGCCAAAGTAAATCAGTGGCCAAGCTGACCTCAACGGGACAAGT        617
GYPE-GYPB           TTTCTCACATTCTATTAGCCAAAGTAAATCAGTGGCCAAGCTGACCTCAATGGGACAAGT        833
>GM19140_D1_F10     TTTCTCACATTCTATTAGCCAAAGTAAATCAGTGGCCAAGCTGACCTCAATGGGACAAGT
>GX0387-C_D1_F10    TTTCTCACATTCTATTAGCCAAAGTAAATCAGTGGCCAAGCTGACCTCAATGGGACAAGT
>GX0531-C_D1_F10    TTTCTCACATTCTATTAGCCAAAGTAAATCAGTGGCCAAGCTGACCTCAATGGGACAAGT
>GX0540-C_D1_F10    TTTCTCACATTCTATTAGCCAAAGTAAATCAGTGGCCAAGCTGACCTCAATGGGACAAGT
>HG02464_D1_F10     TTTCTCACATTCTATTACCCAAAGTAAATCAGGGGCCAAGCTGACCTCAACGGAACAAGT
>HG02545_D1_F10     TTTCTCACATTCTATTACCCAAAGTAAATCATTGGCCAAGCTAACCTCAACGGGACAAGT
>GX0258-C_D1_F10    TTT
>GX0458-C_D1_F10    TTT
>GX0537-C_D1_F10    TTT
>GX0300-C_D1_F10    TTT
                    ******* ****************************************** *********
```

```
                                                       X
GYPB-GYPA          AAATATAATTCTTCCAAATGGAAGTCCTCCAAGTCATAGGTCAATGGTGAGGATATATAA     677
GYPE-GYPB          AAATATAATTCTTCCAAATGGAAGTCCTGCAAGTCATAGGTCAATGGTGAGGATATATAA     893
>GM19140_D1_F10    AAATATAATTCTTCCAAATGGAAGTCCTGCAAGTCATAGGTCAATGGTGAGGATATATAA
>GX0387-C_D1_F10   AAATATAATTCTTCCAAATGGAAGTCCTGCAAGTCATAGGTCAATGGTGAGGATATATAA
>GX0531-C_D1_F10   AAATATAATTCTTCCAAATGGAAGTCCTGCAAGTCATAGGTCAATGGTGAGGATATATAA
>GX0540-C_D1_F10   AAATATAATTCTTCCAAATGGAAGTCCTGCAAGTCATAGGTCAATGGTGAGGATATATAA
>HG02464_D1_F10    AAATATAATTCTTCCAAATGGAAGTCCTGCAAGTCATAGGTCAATGGTGAGGATATATAA
>HG02545_D1_F10    AAATATAATTCTTCCAAATGAAAGTCCTGCAAGTCATAGGTCAATGGTGAGGATATATAA
                   **************************** *******************************

                                                         X               X
GYPB-GYPA          AACACTTACAGGAAGGGCCCTAATAATTGGGAGCGAAAACACAATCCATCACATTCATTT     737
GYPE-GYPB          AACACTTACAGGAAGGGCCCTAATAATTGGGAGCAAAAACACAATCCATCGCATTCATTT     953
>GM19140_D1_F10    AACACTTACAGGAAGGGCCCTAATAATTGGGAGCAAAAACACAATCCATCGCATTCATTT
>GX0387-C_D1_F10   AACACTTACAGGAAGGGCCCTAATAATTGGGAGCAAAACCACAATCCATCGCATTCATTT
>GX0531-C_D1_F10   AACACTTACAGGAAGGGCCCTAATAATTGGGAGCAAAACCACAATCCATCGCATTCATTT
>GX0540-C_D1_F10   AACACTTACAGGAAGGGCCCTAATAATTGGGAGCAAAACCACAATCCATCGCATTCATTT
>HG02464_D1_F10    AACACTTACAGGAAGGGCCCTAATAATTGGAAGCAAAAACACAATCCATCGCATTCATTT
>HG02545_D1_F10    AACACTTACAGGAAGGGCCCTAATAATTGGGAGCAAAAACACAATCCATCGCATTCATTT
                   ********************************** *************** *********

                                                                            X
GYPB-GYPA          ATACTCTTTCTAAGCTTTTGGCATTTGGGTTTGGAAATCTAATTTATTTAGGAGTCTCCT     797
GYPE-GYPB          ATACTCTTTCTAAGCTTTTGGCATTTGGGTTTGGAAATCTAATTTATTTAGGAGTCTTCT     1013
>GM19140_D1_F10    ATACTCTTTCTAAGCTTTTGGCATTTGGGTTTGGAAATCTAATTTATTTAGGAGTCTTCT
>GX0387-C_D1_F10   ATACTCTTTCTAAGCTTTTGGCATTTGGGTTTGGAAATCTAATTTATTTAGGAGTCTTCT
>GX0531-C_D1_F10   ATACTCTTTCTAAGCTTTTGGCATTTGGGTTTGGAAATCTAATTTATTTAGGAGTCTTCT
>GX0540-C_D1_F10   ATACTCTTTCTAAGCTTTTGGCATTTGGGTTTGGAAATCTAATTTATTTAGGAGTCTTCT
>HG02464_D1_F10    ATACTCTTTCTAAGCTTTTGGCATTTGGGTTTGGAAATCTAATTTATTTAGGAGTCTTCT
>HG02545_D1_F10    ATACTCTTTCTAACCTTTTGGCATTTGGGTTTGGAAATCTAATTTATTTAGGAGTCTTCT
                   ********************************************************* **

                                                                            X
GYPB-GYPA          TCAACACCTTCTAGTGATGAGATTTCTTTAACCCAGTTTTCAGTTTCCTTTCTTTAAGAT     857
GYPE-GYPB          TCAACACCTTCTAGTGATGAGATTTCTTTAACCCAGTTTTCAGTTTCCTTTCTTTAAAAT     1073
>GM19140_D1_F10    TCAACACCTTCTAGTGATGAGATTTCTTTAACCCAGTTTTCAGTTTCCTTTCTTTAAAAT
>GX0387-C_D1_F10   TCAACACCTTCTAGTGATGAGATTTCTTTAACCCAGTTTTCAGTTTCCTTTCTTTAAAAT
>GX0531-C_D1_F10   TCAACACCTTCTAGTGATGAGATTTCTTTAACCCAGTTTTCAGTTTCCTTTCTTTAAAAT
>GX0540-C_D1_F10   TCAACACCTTCTAGTGATGAGATTTCTTTAACCCAGTTTTCAGTTTCCTTTCTTTAAAAT
>HG02464_D1_F10    TCAACACCTTCTAGTGATGAGATTTCTTTAACCCAGTTTTCAGTTTCCTTTCTTTAAAAT
>HG02545_D1_F10    TCAACACCTTCTAGTGATGAGATTTCTTTAACCCA
                   ********************************************************* **

                                                                        X
GYPB-GYPA          TATAATGAGATGTGATGTGCGTTGAAGAAACCCCAGAAGTGAGAAACTCTCATTACTACA     917
GYPE-GYPB          TATAATGAGATGTGATGTGCGTTGAAGAAACCCCAGAAGTGAGAAACTCTCATCACTACA     1133
>GM19140_D1_F10    TATAATGAGATGTGATGTGCGTTGAAGAAACCCCAGAAGTGAGAAACTCTCATCACTACA
>GX0387-C_D1_F10   TATAATGAGATGTGATGTGCGTTGAAGAAACCCCAGAAGTGAGAAACTCTCATCACTACA
>GX0531-C_D1_F10   TATAATGAGATGTGATGTGCGTTGAAGAAACCCCAGAAGTGAGAAACTCTCATCACTACA
>GX0540-C_D1_F10   TATAATGAGATGTGATGTGCGTTGAAGAAACCCCAGAAGTGAGAAACTCTCATCACTACA
>HG02464_D1_F10    TATAATGAGATGTGATGTGCGTTGAA
                   ***************************************************** ******

                                     X                              X
GYPB-GYPA          ATGGTGATGTAGACAGTGAAAAACTGTTCCCAGTTGCCTCTAAGTCCATTGTGCTATACT     977
GYPE-GYPB          ATGGTGATGTAGACAGTGGAAAACTGTTCCCAGTTGCCTCTAAGTCCATCGTGCTATACT     1203
>GM19140_D1_F10    ATGGTGATGTAGACAGTGGAAAACTGTTCCCAGTTGCCTCTAAGTCCATCGTGCTATACT
>GX0387-C_D1_F10   ATGGTGATGTAGACAGTGGAAAACTGTTCCCAGTTGCCTCTAAGTCCATCGTGCTATACT
>GX0531-C_D1_F10   ATGGTGATGTAGACAGTGGAAAACTGTTCCCAGTTGCCTCTAAGTCCATCGTGCTATACT
>GX0540-C_D1_F10   ATGGTGATGTAGACAGTGGAAAACTGTTCCCAGTTGCCTCTAAGTCCATCGTGCTATACT
                   ****************** ****************************** **********
```

```
                              X         X
GYPB-GYPA         GCTGGAATCAGGAATCATACATGATCTTTCTGCTGAGAATTGATAGGTCCAGCTTGTTCT     1037
GYPE-GYPB         GCTGGAATCAGGTATCATACATAATCTTTCTGCTGAGAATTGATAGGTCCAGCTTGTTCT     1263
>GM19140_D1_F10   GCTGGAATCAGGTATCATACATAATCTTTCTGCTGAGAATTGATAGGTCCAGCTTGTTCT
>GX0387-C_D1_F10  GCTGGAATCAGGTATCATACATAATCTTTCTGCTGAGAATTGATAGGTCCAGCTTGTTCT
>GX0531-C_D1_F10  GCTGGAATCAGGTATCATACATAATCTTTCTGCTGAGAATTGATAGGTCCAGCTTGTTCT
>GX0540-C_D1_F10  GCTGGAATCAGGTATCATACATAATCTTTCTGCTGAGAATTGATAGGTCCAGCTTGGTCT
>HG02545_D1_R2B5  ----------------------------------------TGATAGGTCCAGCTTGTTCT
>GX0300-C_D1_R2B5 -------------------------------GGTGAGAATTGATAGGTCCAGCTTGTTCT
                  ************ ********* **************************************


                                     X
GYPB-GYPA         ACATCCAAATAATAATTCAACAAATGATTTGTGAGCACCTACCGGGCACTGTGCCAAGTG     1097
GYPE-GYPB         ACATCCAAATAATAATTCAACCAATGATTTGTGAGCACCTACCGGGCACTGTGCCAAGTG     1333
>GM19140_D1_F10   ACATCCAAATAATAATTCAACCAATGATTTGTGAGCACCTACCGGGCACTGTGCCAAGTG
>GM19140_D1_R2B5  ------------------------------------------------------CAAGTG
>GX0387-C_D1_F10  ACATCCAAATAATAATTCAACCAATGATTTGTGAGCACCTACCGGGCCTGGGCCAAGGTG
>GX0531-C_D1_F10  ACATCCAAATAATAATTCAACCAATGATTT------------------------------
>GX0540-C_D1_F10  ACATCCAAATAATAATTCAACCAAGGATTTGTGAGCACCTACCGGGGACTGGGGCCAA--
>GX0540-C_D1_R2B5 ACATCCAAATAATAATTCAACCAATGATTTGTGAGCACCTACCGGGCACTGTGCCAAGTG
>HG02545_D1_R2B5  ACATCCAAATAATAATTCAACCAATGATTTGTGAGCACCTACCGGGCACTGTGCCAAGTG
>GX0458-C_D1_R2B5 ACATCCAAATAATAATTCAACCAATGATTTGTGAGCACCTACCGGGCACTGTGCCAAGTG
>GX0537-C_D1_R2B5 ACATCCAAATAATAATTCAACCAATGATTTGTGAGCACCTACCGGGCACTGTGCCAAGTG
                  ********************* **************************************


                                                                        X
GYPB-GYPA         TGGAAGACACAACCTTCAGTAACTCAAGTTTCCCTTCAGGAACTGACAGCTTAATTGGGA     1157
GYPE-GYPB         TGGAAGACACAACCTTCAGTAACTCAAGTTTCCCTTCAGGAACTGACAGCTTAATTTGGA     1393
>GM19140_D1_R2B5  TGGAAGACACAACCTTCAGTAACTCAAGTTTCCCTTCAGGAACTGACAGCTTAATTTGGA
>GX0540-C_D1_R2B5 TGGAAGACACAACCTTCAGTAACTCAAGTTTCCCTTCAGGAACTGACAGCTTAATTTGGA
>HG02545_D1_R2B5  TGGAAGACACAACCTTCAGTAACTCAAGTTTCCCTTCAGGAACTGACAGCTTAATTTGGA
>GX0458-C_D1_R2B5 TGGAAGACACAACCTTCAGTAACTCAAGTTTCCCTTCAGGAACTGACAGCTTAATTTGGA
>GX0300-C_D1_R2B5 --------------CTTCAGTAACTCAAGTTTCCCTTCAGGAACTGACAGCTTAATTTGGA
>GX0537-C_D1_R2B5 TGGAAGACACAACCTTCAGTAACTCAAGTTTCCCTTCAGGAACTGACAGCTTAATTTGGA
                  ******************************************************** ***


                            X
GYPB-GYPA         AACAAATAAAGTAAGAAGGCAATTGCATTATGGAGTGTCAAGTGGATGATAGTAGTAAGC     1217
GYPE-GYPB         AACAAATAAATTAAGAAGGCAATTGCATTATGGAGTGTCAAGTGGATGATAGTAGTAAGC     1453
>GM19140_D1_R2B5  AACAAATAAATTAAGAAGGCAATTGCATTATGGAGTGTCAAGTGGATGATAGTAGTAAGC
>GX0540-C_D1_R2B5 AACAAATAAATTAAGAAGGCAATTGCATTATGGAGTGTCAAGTGGATGATAGTAGTAAGC
>HG02545_D1_R2B5  AACAAATAAATTAAGAAGGCAGTTGCATTATGGAGTGTCAAGTGGATAATAGTAGTAAGC
>GX0458-C_D1_R2B5 AACAAATAAATTAAGAAGGCAGTTGCATTATGGAGTGTCAAGTGGATAATAGTAGTAAGC
>GX0300-C_D1_R2B5 AACAAATAAATTAAGAAGGCAGTTGCATTATGGAGTGTCAAGTGGATAATAGTAGTAAGC
>GX0537-C_D1_R2B5 AACAAATAAATTAAGAAGGCAGTTGCATTATGGAGTGTCAAGTGGATAATAGTAGTAAGC
                  ********** *************************************************


                                 X                          XX   X
GYPB-GYPA         AAAGGGTGTTATAAGAGTACATAAGAGGGGTACCTAACTCAGACTTGGGGTTCTGGAAAG     1277
GYPE-GYPB         AAAGGGTGTTATAAGGGTACATAAGAGGGGTACCTAACTCAGGGTTGAGGTTCTGGAAAG     1513
>GM19140_D1_R2B5  AAAGGGTGTTATAAGGGTACATAAGAGGGGTACCTAACTCAGGGTTGAGGTTCTGGAAAG
>GX0540-C_D1_R2B5 AAAGGGTGTTATAAGGGTACATAAGAGGGGTACCTAACTCAGGGTTGAGGTTCTGGAAAG
>HG02545_D1_R2B5  AAAGGGTGTTATAAGGGTACATAAGAGGGGTACCTAACTCAGGGTTGAGGTTCTGGAAAG
>GX0458-C_D1_R2B5 AAAGGGTGTTATAAGGGTACATAAGAGGGGTACCTAACTCAGGGTTGAGGTTCTGGAAAG
>GX0300-C_D1_R2B5 AAAGGGTGTTATAAGGGTACATAAGAGGGGTACCTAACTCAGGGTTGAGGTTCTGGAAAG
>GX0537-C_D1_R2B5 AAAGGGTGTTATAAGGGTACATAAGAGGGGTACCTAACTCAGGGTTGAGGTTCTGGAAAG
                  *************** **************************  *** ************
```

```
                                   -X-
GYPB-GYPA         GTTTTCCAGAGCAAGCA---AGACCTAAAGGATGAGTAGACAAGTCACGGGAGAAGTAAG        1334
GYPE-GYPB         GTTTTCCAGAGCAAGCAATGAGACCTAAAGGATGAGTAGACAAGTCACGGGAGAAGTAAG        1573
>GM19140_D1_R2B5  GTTTTCCAGAGCAAGCAATGAGACCTAAAGGATGAGTAGACAAGTCACGGGAGAAGTAAG
>GX0540-C_D1_R2B5 GTTTTCCAGAGCAAGCAATGAGACCTAAAGGATGAGTAGACAAGTCACGGGAGAAGTAAG
>HG02545_D1_R2B5  GTTTTCCAGAGCAAGCAATGAGACCTAAAGGATGAGTAGACAAGTCACGGGAGAAGTAAG
>GX0458-C_D1_R2B5 GTTTTCCAGAGCAAGCAATGAGACCTAAAGGATGAGTAGACAAGTCACGGGAGAAGTAAG
>GX0300-C_D1_R2B5 GTTTTCCAGAGCAAGCAATGAGACCTAAAGGATGAGTAGACAAGTCACGGGAGAAGTAAG
>GX0537-C_D1_R2B5 GTTTTCCAGAGCAAGCAATGAGACCTAAAGGATGAGTAGACAAGTCACGGGAGAAGTAAG
                  *****************   ****************************************


                                                            X        XX
GYPB-GYPA         AACATTTTAAGCAGAGAAAACAGCATGAGCAATGGTCTGATACCAAGAAAGAGAGAGATA        1394
GYPE-GYPB         AACATTTTAAGCAGAGAAAACAGCATGAGCAATGGTCTGATATCAAGAAAGGCAGAGATA        1633
>GM19140_D1_R2B5  AACATTTTAAGCAGAGAAAACAGCATGAGCAATGGTCTGATATCAAGAAAGGCAGAGATA
>GX0540-C_D1_R2B5 AACATTTTAAGCAGAGAAAACAGCATGAGCAATGGTCTGATATCAAGAAAGGCAGAGATA
>HG02464_D1_R2B5  -------------------------------------TGATATCAAGAAAGGCAGAGATA
>HG02545_D1_R2B5  AACATTTTAAGCAGAGAAAACAGCATGAGCAATGGTCTGATATCAAGAAAGGCAGAGATA
>GX0458-C_D1_R2B5 AACATTTTAAGCAGAGAAAACAGCATGAGCAATGGTCTGATATCAAGAAAGGCAGAGATA
>GX0300-C_D1_R2B5 AACATTTTAAGCAGAGAAAACAGCATGAGCAATGGTCTGATATCAAGAAAGGCAGAGATA
>GX0537-C_D1_R2B5 AACATTTTAAGCAGAGAAAACAGCATGAGCAATGGTCTGATATCAAGAAAGGCAGAGATA
                  ****************************************** ********  *******


                                                                            X
GYPB-GYPA         GTGCAAGAATACACAATGTAGTAGTTTTCAAAACTGTTTGAAATTTTAATCTCACATTGG        1454
GYPE-GYPB         GTGCAAGAATACACAATGTAGTAGTTTTCAAAACTGTTTGAAATTTTAATCTCACATTTG        1693
>GM19140_D1_R2B5  GTGCAAGAATACACAATGTAGTAGTTTTCAAAACTGTTTGAAATTTTAATCTCACATTTG
>GX0540-C_D1_R2B5 GTGCAAGAATACACAATGTAGTAGTTTTCAAAACTGTTTGAAATTTTAATCTCACATTTG
>HG02464_D1_R2B5  GTGCAAGAATACACAATGTAGTAGTTTTCAAAACTGTTTGAAATTTTAATCTCACATTTG
>HG02545_D1_R2B5  GTGCAAGAATACACAATGTAGTAGTTTTCAAAACT-TT-GAAATTTTAATCTCACATTTG
>GX0258-C_D1_R2B5 ----------------------------------T-TT-GAAATTTTAATCTCACATTTG
>GX0458-C_D1_R2B5 GTGCAAGAATACACAATGTAGTAGTTTTCAAAACTGTTTGAAATTTTAATCTCACATTTG
>GX0300-C_D1_R2B5 GTGCAAGAATACACAATGTAGTAGTTTTCAAAACTGTTTGAAATTTTAATCTCACATTTG
>GX0537-C_D1_R2B5 GTGCAAGAATACACAATGTAGTAGTTTTCAAAACTGTTTGAAATTTTAATCTCACATTTG
                  ********************************************************** *


                                   X                 XX
GYPB-GYPA         TTCTTTTCAATGTCTAGTATATCAATTCATTCTGCCGTTTACCTTTGTAAAGATGAATTT        1514
GYPE-GYPB         TTCTTTTCAATGTCTAGGATATCAATTCATTCTGCGATTTACCTTTGTAAAGATGAATTT        1753
>GM19140_D1_R2B5  TTCTTTTCAATGTCTAGGATATCAATTCATTCTGCGATTTACCTTTGTAAAGATGAATTT
>GX0540-C_D1_R2B5 TTCTTTTCAATGTCTAGGATATCAATTCATTCTGCGATTTACCTTTGTAAAGATGAATTT
>HG02464_D1_R2B5  TTCTTTTCAATGTCTAGGATATCAATTCATTCTGCGATTTACCTTTGTAAAGATGAATTT
>HG02545_D1_R2B5  TTCTTTTCAATGTCTAGGATATCAATTCATTCTGCGATTTACCTTTGTAAAGATGAATTT
>GX0258-C_D1_R2B5 TTCTTTTCAATGTCTAGGATATCAATTCATTCTGCGATTTACCTTTGTAAAGATGAATTT
>GX0458-C_D1_R2B5 TTCTTTTCAATGTCTAGGATATCAATTCATTCTGCGATTTACCTTTGTAAAGATGAATTT
>GX0300-C_D1_R2B5 TTCTTTTCAATGTCTAGGATATCAATTCATTCTGCGATTTACCTTTGTAAAGATGAATTT
>GX0537-C_D1_R2B5 TTCTTTTCAATGTCTAGGATATCAATTCATTCTGCGATTTACCTTTGTAAAGATGAATTT
                  ***************** *****************  ***********************


                                                       X
GYPB-GYPA         AGCCCTGGTTCTCTATATGAATACTAGAGGAAGTATACTGATAAAAGAAAAGGAACAAAT        1574
GYPE-GYPB         AGCCCTGGTTCTCTATATGAATACTAGAGGAAGTATATTGATAAAAGAAAAGGAACAAAT        1813
>GM19140_D1_R2B5  AGCCCTGGTTCTCTATATGAATACTAGAGGAAGTATATTGATAAAAGAAAAGGAACAAAT
>GX0540-C_D1_R2B5 AGCCCTGGTTCTCTATATGAATACTAGAGGAAGTATATTGATAAAAGAAAAGGAACAAAT
>HG02464_D1_R2B5  AGCCCTGGTTCTCTATATGAATACTAGAGGAAGTATATTGATAAAAGAAAAGGAACAAAT
>HG02545_D1_R2B5  AGCCCTGGTTCTCTATATGAATACTAGAGGAAGTATATTGATAAAAGAAAAGGAACAAAT
>GX0258-C_D1_R2B5 AGCCCTGGTTCTCTATATGAATACTAGAGGAAGTATATTGATAAAAGAAAAGGAACAAAT
>GX0458-C_D1_R2B5 AGCCCTGGTTCTCTATATGAATACTAGAGGAAGTATATTGATAAAAGAAAAGGAACAAAT
>GX0300-C_D1_R2B5 AGCCCTGGTTCTCTATATGAATACTAGAGGAAGTATATTGATAAAAGAAAAGGAACAAAT
>GX0537-C_D1_R2B5 AGCCCTGGTTCTCTATATGAATACTAGAGGAAGTATATTGATAAAAGAAAAGGAACAAAT
                  ************************************* **********************
```

```
                                                                     XX
GYPB-GYPA          AATGTAGTCATTTCTAAGATTTATTTTTATTAAAATGTAACACTCACAAACT--AAGTGT     1632
GYPE-GYPB          -ATGTAGTCATTTCTAAGATTTATTTTTATTAAAATGTAACACTCACAAACTGTAAGTGT     1872
>GM19140_D1_R2B5   -ATGTAGTCATTTCTAAGATTTATTTTTATTAAAATGTAACACTCACAAACTGTAAGTGT
>GX0387-C_D1_R2B5  -ATATACTCATTTATGAGATTTATTTTTATTAAAATGTCACTCTCACAAACTGTAAGTGT   **
>GX0531-C_D1_R2B5  --------------------------------------CACTCTCACAAACTGTAAGTGT   **
>GX0540-C_D1_R2B5  -ATGTAGTCATTTCTAAGATTTATTTTTATTAAAATGTAACACTCACAAACTGTAAGTGT
>HG02464_D1_R2B5   -ATGTAGTCATTTCTAAGATTTATTTTTATTAAAATGTAACACTCACAAACTGTAAGTGT
>HG02545_D1_R2B5   -ATGTAGTCATTTCTAAGATTTATTTTTATTAAAATGTAACACTCACAAACTGTAAGTGT
>GX0258-C_D1_R2B5  -ATGTAGTCATTTCTAAGATTTATTTTTATTAAAATGTAACACTCACAAACTGTAAGTGT
>GX0458-C_D1_R2B5  -ATGTAGTCATTTCTAAGATTTATTTTTATTAAAATGTAACACTCACAAACTGTAAGTGT
>GX0300-C_D1_R2B5  -ATGTAGTCATTTCTAAGATTTATTTTTATTAAAATGTAACACTCACAAACTGTAAGTGT
>GX0537-C_D1_R2B5  -ATGTAGTCATTTCTAAGATTTATTTTTATTAAAATGTAACACTCACAAACTGTAAGTGT
                    ***************************************************  ******


                   -----------------------Z----------------------
                   X X   BB   X      BB        XX        XXXXXXXX
GYPB-GYPA          AT-ACTCATATGTATATATACACACATA--CACACACACACACACATACATGAGTATATGTGTGA   1691
GYPE-GYPB          GTAACTCATATATATATATACACACATATACACACACA--------TACATGAGTATATGTGTGA   1924
>GM19140_D1_R2B5   GTAACTCATATATATATA--CACACATATACACACACA--------TACATGAGTATATGTGTGA
>GX0387-C_D1_R2B5  GTATCTCATATATATATA--CACACATATACACACACA--------TACATGAGTATATGTGTGA   **
>GX0531-C_D1_R2B5  GTATCTCATATATATATA--CACACATATACACACACA--------TACATGAGTATATGTGTGA   **
>GX0540-C_D1_R2B5  GTAACTCATATATATATA--CACACATATACACACACA--------TACATGAGTATATGTGTGA
>HG02464_D1_R2B5   GTAACT--TATATATATATACACACATATACACACACA--------TACATGAGTATATGTGTGA
>HG02545_D1_R2B5   GTAACT--TATATATATAT-CACACATATACACACACA--------TACATGAGTATATGTGTGA
>GX0258-C_D1_R2B5  GTAACT--TATATATATATACACACATATACACACACA--------TACATGAGTATATGTGTGA
>GX0458-C_D1_R2B5  GTAACT--TATATATATATACACACATATACACACACA--------TACATGAGTATATGTGTGA
>GX0300-C_D1_R2B5  GTAACT--TATATATATATACACACATATACACACACA--------TACATGAGTATATGTGTGA
>GX0537-C_D1_R2B5  GTAACT--TATATATATATACACACATATACACACACA--------TACATGAGTATATGTGTGA
                    * ***  *** ******  ********  ********        *******************


GYPB-GYPA          AACTGTCACTCAAATCAAGACATAGAACATTTCAGATGTATTCCAGAGATCATGGTGGAT     1751
GYPE-GYPB          AACTGTCACTCAAATCAAGACATAGAACATTTCAGATGTATTCCAGAGATCATGGTGGAT     1984
>GM19140_D1_R2B5   AACTGTCACTCAAATCAAGACATAGAACATTTCAGATGTATTCCAGAGATCATGGTGGAT
>GX0387-C_D1_R2B5  AACTGTCACTCAAATCAAGACATAGAACATTTCAGATGTATTCCAGAGATCATGGTGGAT
>GX0531-C_D1_R2B5  AACTGTCACTCAAATCAAGACATAGAACATTTCAGATGTATTCCAGAGATCATGGTGGAT
>GX0540-C_D1_R2B5  AACTGTCACTCAAATCAAGACATAGAACATTTCAGATGTATTCCAGAGATCATGGTGGAT
>HG02464_D1_R2B5   AACTGTCACTCAAATCAAGACATAGAACATTTCAGATGTATTCCAGAGATCATGGTGGAT
>HG02545_D1_R2B5   AACTGTCACTCAAATCAAGACATAGAACATTTCAGATGTATTCCAGAGATCATGGTGGAT
>GX0258-C_D1_R2B5  AACTGTCACTCAAATCAAGACATAGAACATTTCAGATGTATTCCAGAGATCATGGTGGAT
>GX0458-C_D1_R2B5  AACTGTCACTCAAATCAAGACATAGAACATTTCAGATGTATTCCAGAGATCATGGTGGAT
>GX0300-C_D1_R2B5  AACTGTCACTCAAATCAAGACATAGAACATTTCAGATGTATTCCAGAGATCATGGTGGAT
>GX0537-C_D1_R2B5  AACTGTCACTCAAATCAAGACATAGAACATTTCAGATGTATTCCAGAGATCATGGTGGAT
                   ************************************************************


                                                   ZZ
GYPB-GYPA          GGGAGGCAGGACTGGATTGCAGCTCCCACTTGAACAGACAAAGCAGCGTGTGGAGGCTTG     1811
GYPE-GYPB          GGGAGGCAGGACTGGATTGCAGCTCCCACTTGGACAGACAAAGCAGCGTGTGGAGGCTTG     2044
>GM19140_D1_R2B5   GGGAGGCAGGACTGGATTGCAGCTCCCACTTGGACAGACAAAGCAGCGTGTGGAGGCTTG
>GX0387-C_D1_R2B5  GGGAGGCAGGACTGGATTGCAGCTCCCACTTGGACAGACAAAGCAGCGTGTGGAGGCTTG
>GX0531-C_D1_R2B5  GGGAGGCAGGACTGGATTGCAGCTCCCACTTGGACAGACAAAGCAGCGTGTGGAGGCTTG
>GX0540-C_D1_R2B5  GGGAGGCAGGACTGGATTGCAGCTCCCACTTGGACAGACAAAGCAGCGTGTGGAGGCTTG
>HG02464_D1_R2B5   GGGAGGCAGGACTGGATTGCAGCTCCCACTTGAACAGACAAAGCAGCGTGTGGAGGCTTG
>HG02545_D1_R2B5   GGGAGGCAGGACTGGATTGCAGCTCCCACTTGAACAGACAAAGCAGCGTGTGGAGGCTTG
>GX0258-C_D1_R2B5  GGGAGGCAGGACTGGATTGCAGCTCCCACTTGAACAGACAAAGCAGCGTGTGGAGGCTTG
>GX0458-C_D1_R2B5  GGGAGGCAGGACTGGATTGCAGCTCCCACTTGAACAGACAAAGCAGCGTGTGGAGGCTTG
>GX0300-C_D1_R2B5  GGGAGGCAGGACTGGATTGCAGCTCCCACTTGAACAGACAAAGCAGCGTGTGGAGGCTTG
>GX0537-C_D1_R2B5  GGGAGGCAGGACTGGATTGCAGCTCCCACTTGAACAGACAAAGCAGCGTGTGGAGGCTTG
                   ******************************** ***************************
```

```
                                                     XX                      X
GYPB-GYPA         TATCATGAACTTTGACTGCAGGAATAAATCAGGAAAGCTGAGAGAACCCACAGACCCTCT        1871
GYPE-GYPB         TATCATGAACTTTGACTGCAGGAATAAATCAGGAACACTGAGAGAACCCACAGACCCTCA        2104
>GM19140_D1_R2B5  TATCATGAACTTTGACTGCAGGAATAAATCAGGAACACTGAGAGAACCCACAGACCCTCA
>GX0387-C_D1_R2B5 TATCATGAACTTTGACTGCAGGAATAAATCAGGAACACTGAGAGAACCCACAGACCCTCA
>GX0531-C_D1_R2B5 TATCATGAACTTTGACTGCAGGAATAAATCAGGAACACTGAGAGAACCCACAGACCCTCA
>GX0540-C_D1_R2B5 TATCATGAACTTTGACTGCAGGAATAAATCAGGAACACTGAGAGAACCCACAGACCCTCA
>HG02464_D1_R2B5  TATCATGAACTTTGACTGCAGGAATAAATCAGGAAAGCTGAGAGAACCCACAGACCCTCT
>HG02545_D1_R2B5  TATCATGAACTTTGACTGCAGGAATAAATCAGGAAAGCTGAGAGAACCCACAGACCCTCT
>GX0258-C_D1_R2B5 TATCATGAACTTTGACTGCAGGAATAAATCAGGAAAGCTGAGAGAACCCACAGACCCTCT
>GX0458-C_D1_R2B5 TATCATGAACTTTGACTGCAGGAATAAATCAGGAAAGCTGAGAGAACCCACAGACCCTCT
>GX0300-C_D1_R2B5 TATCATGAACTTTGACTGCAGGAATAAATCAGGAAAGCTGAGAGAACCCACAGACCCTCT
>GX0537-C_D1_R2B5 TATCATGAACTTTGACTGCAGGAATAAATCAGGAAAGCTGAGAGAACCCACAGACCCTCT
                  ***********************************  ********************** 


                     X    X      X
GYPB-GYPA         GAAGGAAGTGGATTGTTCCTGCAGGTCTCAGGAGACACCCCAAATGCTGTGGGAGCCCAA        1931
GYPE-GYPB         GAAAGAAGCGGATTGCTCCTGCAGGTCTCAGGAGACACCCCAAATGCTGTGGGAGCCCAA        2164
>GM19140_D1_R2B5  GAAAGAAGCGGATTGCTCCTGCAGGTCTCAGGAGACACCCCAAATGCTGTGGGAGCCCAA
>GX0387-C_D1_R2B5 GAAAGAAGCGGATTGCTCCTGCAGGTCTCAGGAGACACCCCAAATGCTGTGGGAGCCCAA
>GX0531-C_D1_R2B5 GAAAGAAGCGGATTGCTCCTGCAGGTCTCAGGAGACACCCCAAATGCTGTGGGAGCCCAA
>GX0540-C_D1_R2B5 GAAAGAAGCGGATTGCTCCTGCAGGTCTCAGGAGACACCCCAAATGCTGTGGGAGCCCAA
>HG02464_D1_R2B5  GAAGGAAGTGGATTGCTCCTGCAGGTCTCAGGAGACACCCCAAATGCTGTGGGAGCCCAA
>HG02545_D1_R2B5  GAAGGAAGTGGATTGCTCCTGCAGGTCTCAGGAGACACCCCAAATGCTGTGGGAGCCCAA
>GX0258-C_D1_R2B5 GAAGGAAGTGGATTGCTCCTGCAGGTCTCAGGAGACACCCCAAATGCTGTGGGAGCCCAA
>GX0458-C_D1_R2B5 GAAGGAAGTGGATTGCTCCTGCAGGTCTCAGGAGACACCCCAAATGCTGTGGGAGCCCAA
>GX0300-C_D1_R2B5 GAAGGAAGTGGATTGCTCCTGCAGGTCTCAGGAGACACCCCAAATGCTGTGGGAGCCCAA
>GX0537-C_D1_R2B5 GAAGGAAGTGGATTGCTCCTGCAGGTCTCAGGAGACACCCCAAATGCTGTGGGAGCCCAA
                  *** **** ****** ********************************************
                         AciI site (discriminating)


GYPB-GYPA         ACTGCAAACTGTGGAAGTGGGAAAGGGGAATAGTCAGCTCCTGAACACACATCCTCACTG        1991
GYPE-GYPB         ACTGCAAACTGTGGAAGTGGGAAAGGGGAATAGTCAGCTCCTGAACACACATCCTCACTG        2224
>GM19140_D1_R2B5  ACTGCAAACTGTGGAAGTGGGAAAGGGGAATAGTCAGCTCCGGAACACACATCCTCACTG
>GX0387-C_D1_R2B5 ACTGCAAACTGTGGAAGTGGGAAAGGGGAATAGTCAGCTCCGGAACACACATCCTCACTG
>GX0531-C_D1_R2B5 ACTGCAAACTGTGGAAGTGGGAAAGGGGAATAGTCAGCTCCGGAACACACATCCTCACTG
>GX0540-C_D1_R2B5 ACTGCAAACTGTGGAAGTGGGAAAGGGGAATAGTCAGCTCCGGAACACACATCCTCACTG
>HG02464_D1_R2B5  ACTGCAAACTGTGGAAGTGGGAAAGGGGAATAGTCAGCTCCTGAACACACATCCTCACTG
>HG02545_D1_R2B5  ACTGCAAACTGTGGAAGTGGGAAAGGGGAATAGTCAGCTCCTGAACACACATCCTCACTG
>GX0258-C_D1_R2B5 ACTGCAAACTGTGGAAGTGGGAAAGGGGAATAGTCAGCTCCTGAACACACATCCTCACTG
>GX0458-C_D1_R2B5 ACTGCAAACTGTGGAAGTGGGAAAGGGGAATAGTCAGCTCCTGAACACACATCCTCACTG
>GX0300-C_D1_R2B5 ACTGCAAACTGTGGAAGTGGGAAAGGGGAATAGTCAGCTCCTGAACACACATCCTCACTG
>GX0537-C_D1_R2B5 ACTGCAAACTGTGGAAGTGGGAAAGGGGAATAGTCAGCTCCTGAACACACATCCTCACTG
                  ************************************************************


GYPB-GYPA         GGGAACCTAAAGGTCTAGATCACAGGAGAAGATTTTGACCTTACTTGGAGCTGAGTCAAT        2051
GYPE-GYPB         GGGAACCTAAAGGTCTAGATCACAGGAGAAGATTTTGACCTTACTTGGAGCTGAGTCAAT        2284
>GM19140_D1_R2B5  GGGAACCTAAAGGTCTAGATCACAGGAGAAGATTTTGACCTTACTTGGAGCTGAGTCAAT
>GX0387-C_D1_R2B5 GGGAACCTAAAGGTCTAGATCACAGGAGAAGATTTTGACCTTACTTGGAGCTGAGTCAAT
>GX0531-C_D1_R2B5 GGGAACCTAAAGGTCTAGATCACAGGAGAAGATTTTGACCTTACTTGGAGCTGAGTCAAT
>GX0540-C_D1_R2B5 GGGAACCTAAAGGTCTAGATCACAGGAGAAGATTTTGACCTTACTTGGAGCTGAGTCAAT
>HG02464_D1_R2B5  GGGAACCTAAAGGTCTAGATCACAGGAGAAGATTTTGACCTTACTTGGAGCTGAGTCAAT
>HG02545_D1_R2B5  GGGAACCTAAAGGTCTAGATCACAGGAGAAGATTTTGACCTTACTTGGAGCTGAGTCAAT
>GX0258-C_D1_R2B5 GGGAACCTAAAGGTCTAGATCACAGGAGAAGATTTTGACCTTACTTGGAGCTGAGTCAAT
>GX0458-C_D1_R2B5 GGGAACCTAAAGGTCTAGATCACAGGAGAAGATTTTGACCTTACTTGGAGCTGAGTCAAT
>GX0300-C_D1_R2B5 GGGAACCTAAAGGTCTAGATCACAGGAGAAGATTTTGACCTTACTTGGAGCTGAGTCAAT
>GX0537-C_D1_R2B5 GGGAACCTAAAGGTCTAGATCACAGGAGAAGATTTTGACCTTACTTGGAGCTGAGTCAAT
                  ************************************************************
```

```
                                                                      X   B  X
GYPB-GYPA          TTAGAGAGCCAAGTGACATACACTGCTAGAGAAAGCAGCGCGAAAAGCCCTGTGGGCTCA        2111
GYPE-GYPB          TTAGAGAGCCAAGTGACATACACTGCTAGAGAAAGCAGGGCGTAAGGCCCTGTGGGCTCA        2344
>GM19140_D1_R2B5   TTAGAGAGCCAAGTGACATACACTGCTAGAGAAAGCAGGGCGAAAGGCCCTGTGGGCTCA
>GX0387-C_D1_R2B5  TTAGAGAGCCAAGTGACATACACTGCTAGAGAAAGCAGGGCGAAAGGCCCTGTGGGCTCA
>GX0531-C_D1_R2B5  TTAGAGAGCCAAGTGACATACACTGCTAGAGAAAGCAGGGCGTAAGGCCCTGTGGGCTCA
>GX0540-C_D1_R2B5  TTAGAGAGCCAAGTGACATACACTGCTAGAGAAAGCAGGGCGAAAGGCCCTGTGGGCTCA
>HG02464_D1_R2B5   TTAGAGAGCCAAGTGACATACACTGCTAGAGAAAGCAGCGCGAAAAGCCCTGTGGGCTCA
>HG02545_D1_R2B5   TTAGAGAGCCAAGTGACATACACTGCTAGAGAAAGCAGCGCGAAAAGCCCTGTGGGCTCA
>GX0258-C_D1_R2B5  TTAGAGAGCCAAGTGACATACACTGCTAGAGAAAGCAGCGCGAAAAGCCCTGTGGGCTCA
>GX0458-C_D1_R2B5  TTAGAGAGCCAAGTGACATACACTGCTAGAGAAAGCAGCGCGAAAAGCCCTGTGGGCTCA
>GX0300-C_D1_R2B5  TTAGAGAGCCAAGTGACATACACTGCTAGAGAAAGCAGCGCGAAAAGCCCTGTGGGCTCA
>GX0537-C_D1_R2B5  TTAGAGAGCCAAGTGACATACACTGCTAGAGAAAGCAGCGCGAAAAGCCCTGTGGGCTCA
                   ************************************** *** ** **************
```

```
                                                                              ← GYP_DEL1_R2B5 (rev)
GYPB-GYPA          CTGGGTTCCCTAGCCATCCATTTCTGCCTTGCCTCACAGGGGTCCTTGAGGAGGGCTACCAGAGGCACTGGG        2171
GYPE-GYPB          CTGGGTTCCCTAGCCATCCATTTCTGCCTTGCCTCACGGGGGTCCTTGAGGAGGGCTACCAGAGGCACTGGG        2404
>GM19140_D1_R2B5   CTGGGTTCCCTAGCC---------------------------------------------------------
>GX0387-C_D1_R2B5  CTGGGTTCCCTAGCCATCCA----------------------------------------------------
>GX0531-C_D1_R2B5  CTGGGTTCCC--------------------------------------------------------------
>GX0540-C_D1_R2B5  CTGGGTTCCCTAGCCATCCA----------------------------------------------------
>HG02464_D1_R2B5   CTGGG-------------------------------------------------------------------
>HG02545_D1_R2B5   CTGGGTTCCCTAGCC---------------------------------------------------------
>GX0458-C_D1_R2B5  CTGG--------------------------------------------------------------------
>GX0300-C_D1_R2B5  CTGGGTTCCCTAGCCATCC-----------------------------------------------------
>GX0537-C_D1_R2B5  CTGGGTCCC---------------------------------------------------------------
                   ************************************* **********************************
```

Amuzu et al, Supplementary File 5 DEL2 Sanger Sequence pile-ups

Sanger Sequence alignments for DEL2 PCR products.
Coordinates are with respect to GRCH37 and GRCh38 and the forward strand of the human reference sequence.

GYP target	Primers	Sequence (5'-3')	Dir	GC (%)	T_m (°C)	GYP region	GRCh37 location	GRCh38 location
DEL2	DEL2_GYPEBAc_F3	GGTCATGAGAAAACGTTTGAATTTTCCAG	Fwd	37.9	59	5'-GYPE	4:144791036-144791060	4:143869883-143869907
						GYPE-GYPB	4:144911831-144911855	4:143990678-143990702
						GYPB-GYPA	4:145015096-145015120	4:144093943-144093967
DEL2	DEL2_GYPBAs_R3	CAGTTCTGCCAACTCTCATCTT	Rev	45	56	GYPB-GYPA	4:145017216-145017238	4:144096063-144096085
DEL2	DEL2_BP_seq_Rev1	CTATGGGTCCCTCTCTGTGGA	Rev			5'-GYPE	4:144792394-144792414	4:143870624-143870648
						GYPE-GYPB	4:144913188-144913208	4:143991419-143991443
						GYPB-GYPA	4:145016443-145016458	4:144094684-144094708
DEL2	DEL2_BP_seq_Fwd	CATGTCTCACATCCAGTTAATGCTG	Fwd			5'-GYPE	4:144791777-144791801	4:143871241-143871261
						GYPE-GYPB	4:144912572-144912596	4:143992035-143992055
						GYPB-GYPA	4:145015837-145015861	4:144095290-144095305

Table 1: Primer sequences used for DEL2 PCR and Sanger Sequencing

**Putative Breakpoint:**	**GRCh37**	**GRCh38**	
GYPE-GYPB:	4:144912872-144913001	4:143991719-143991848	~86kb 3' to the GYPE ATG start site (GRCH37:4:144826660 and GRCH37:4:143905507).
GYPB-GYPA:	4:145016127-145016256	4:144094974-144095103	~76kb 3' to the GYPB ATG start site (GRCH37:4:144940440 and GRCH38: 4:144019287).

BsrBI (CCGCTC) discriminating sites (common to both the normal and hybrid products are highlighted in Magenta in the pile-up below)

	**SITE**	**#1 GRCh37**			**#2 GRCh37**		
**BsrBI** (CCGCTC) discriminating sites (magenta):	5'-GYPE:	4:144791837-144791842	NOT-CUT	(CAGCTC)	4:144791869-144791874	NOT-CUT	(CGCCTC)
	GYPE-GYPB:	4:144912632-144912637	CUT	(CCGCTC)	4:144912661-144912666	CUT	(CCGCTC)
	GYPB-GYPA:	4:145015897-145015902	NOT-CUT	(CTGCTC)	4: 145015926-144094777	NOT-CUT	(CCCCTC)

DEL2 alignments of Sanger sequence data with the 5'-GYPE, GYPE-GYPB and GYPB-GYPA homologous reference sequences across the PCR region

PCR/Sequencing primers are indicated and highlighted in grey with red text

Differences between sequences:

- Yellow highlights the GYPE-GYPB reference nucleotides;
- green highlights the GYPB-GYPA reference nucleotides;
- Grey highlights the 5'-GYPE sequence where different from the others, and other bases at a given position not matching the GYPE-GYPB or GYPB-GYPA bases.
- magenta highlights the BsrBI restrictions sites;
- blue highlights the putative breakpoint region.

Marked-up bases (bases that are different between the reference sequences):

- X; discriminates between GYPE-GYPB and GYPB-GYPA regions.
- B; non-discriminatory 'polymorphism' for GYPE-GYPB and GYPB-GYPA.
- Z; 5' region of the putative breakpoint. Last discriminatory bases between GYPE-GYPB and the GYPB-GYPA regions that separate normal samples from DEL2 samples. From this point moving 5' to the common forward PCR primer, all potential discriminatory bases show the different sequences for normal and DEL2 samples. This region forms the putative 5' end of the breakpoint region for DEL2.
- ZZ; 3' end of the breakpoint. These are the first polymorphic bases between reference sequences that show no differences between sample sequences, and that they indicate GYPB-GYPA origin. Similarly all other discriminatory bases 3' from here up to the specific GYPB-GYPA reverse primer indicates all sample sequences are GYPB-GYPA. The putative breakpoint region is therefore within this 130 base region (z to zz) highlighted in blue.

Samples (see main text Table 2):

- GM19140; 1000G/HapMap cell line - normal
- GX0387; Ghanaian with normal PCR for DEL1/DEL2/Dup4
- GX0540; Ghanaian with normal PCR for DEL1/DEL2/Dup4
- GX0531; Ghanaian with normal PCR for DEL1/DEL2/Dup4

- HG03385; 1000G/HapMap cell line – DEL2 homozygote
- GX0403; Ghanaian DEL2 homozygote & normal PCR for DEL1/Dup4

- GX0300; Ghanaian DEL1/DEL2 heterozygote & normal PCR for Dup4

All sequences look like the GYPB-GYPA region from the specific DEL2_GYPBAs_R3 primer through to region highlighted in blue (putative breakpoint region).
From the common forward primer (DEL2_GYPEBAc_F3) through to the putative breakpoint, the sequences fall into 2 clear groups; those matching the full GYPB-GYPA sequence (normal sequences); and those matching the part GYPB-GYPA and part GYPE-GYPB sequences (samples with the DEL2 deletion).
In the sequence alignments below, the numbering refers to the reference sequences; B-A indicates sample sequences matching the GYPB-GYPA reference, while E-B indicates sample sequences matching the GYPE-GYPB reference.

The 5'-GYP sequence is included as this is also an homologous sequence region and because both forward and reverse primers are not in wholly unique sequence regions. The specific reverse primer is dependent on several mismatches to the GYPE-GYPB and 5'-GYPE sequences for its specificity (unlike the DEL1 assay where a wholly specific primer can be used). It can be seen from the sequences obtained that they do not match the 5'-GYPE sequence; particular from the 5'-GYPE specific bases/indels through the sequence.

Reference sequences from GRCh37 as per coordinates indicated in the primer table above.

```
                                            DEL2_GYPEBAc_F3 =>
GYPE_F3-R3                 GGTCATAAGAAAACGTTTGAATTTTTCCGATCAGTCAGTCATTGTATTAGTTCATTCCCA        60
GYPE-GYPB_F3-R3            GGTCATGAGAAAACGTTTGAATTTTTCCAGTCAGTCAGTCATTGTATTAGTTCATTCCCA        60
GYPB-GYPA_F3-R3            GGTCATAAGAAAATGTTTGAATTTTTCCAGTCAGTCAGTCATTGTATTAGTTCATTCCCA        60
GM19140_NORM_concensus     ------------------------------------------------------------
>GX0387_concensus          ---------------------------------------TCTTGTATTAGTTCATTCCCA
>GX0531_concensus          ---------------------------------------TCTTGTATTAGTTCATTCCCA
>GX0540_concensus          ---------------------------------------TCTTGTATTAGTTCATTCCCA
HG03385_DEL2_hom           ------------------------------------------------------------
>GX0403-C_D2_concensus     ------------------------------------------------------------
>GX0300-C_D2_concensus     ------------------------------------------------------TTCCCA
                                                                                 ******


GYPE_F3-R3                 TGCTGCTATAAAGAAGTGCCTGAGACTGGGTAATTTATAAAGGAAAGAAGTTTAATTGAC        120
GYPE-GYPB_F3-R3            TGCTGCTATAAAGAAGTGCCTGAGACTGGGTAATTTATAAAGGAAAGAAGTTTAATTGAC        120
GYPB-GYPA_F3-R3            TGCTGCTATAAAGAAGTGCCTGAGACTGGGTAATTTATAAAGGAAAGAAGTTTAATTGAC        120
GM19140_NORM_concensus     TGCTGCTATAAAGAAGTGCCTGAGACTGGGTAATTTATAAAGGAAAGAAGTTTAATTGAC        B-A
>GX0387_concensus          TGCTGCTATAAAGAAGTGCCTGAGACTGGGTAATTTAYAAAGGAAAGAAGTTTAATTGAC
>GX0531_concensus          TGCTGCTATAAAGAAGTGCCTGAGACTGGGTAATTTATAAAGGAAAGAAGTTTAATTGAC
>GX0540_concensus          TGCTGCTATAAAGAAGTGCCTGAGACTGGGTAATTTATAAAGGAAAGAAGTTTAATTGAC
HG03385_DEL2_hom           --CTGCTATAAAGAAGTGCCTGAGACTGGGTAATTTATAAAGGAAAGAAGTTTAATTGAC        E-B
>GX0403-C_D2_concensus     TGCTGCTATAAAGAAGTGCCTGAGACTGGGTAATTTATAAAGGAAAGAAGTTTAATTGAC
>GX0300-C_D2_concensus     TGCTGCTATAAAGAAGTGCCTGAGACTGGGTAATTTATAAAGGAAAGAAGTTTAATTGAC
>GX0440-C_D2_concensus     TGCTGCTATAAAGAAGTGCCTGAGACTGGGTAATTTATAAAGGAAAGAAGTTTAATTGAC
                             **********************************************************


                                                B
GYPE_F3-R3                 TCACAATTCTGCGTGGCTGAGAAGGTTTCAGGAAACTTACAATCGTGGCAGAAGGGGAAG        180
GYPE-GYPB_F3-R3            TCACAATTCTGCGTGGCTGAGAAGGTTTCAGGAAACTTACAATCGTGGCAGAAGGGGAAG        180
GYPB-GYPA_F3-R3            TCACAATTCTGCGTGGCTGAGAAGGTTTCAGGAAACTTACAATCGTGGCAGAAGGGGAAG        180
GM19140_NORM_concensus     TCACAATTCTGCGTGGCTGAGAAGGTTTCAGGAAACTTACAATCGTGGCAGAAGGGGAAG        B-A
>GX0387_concensus          TCACAATTCTGCGTGGCTGAGAAGGTTTCAGGAAACTTACAATCGTGGCAGAAGGGGAAG
>GX0531_concensus          TCACAATTCTGCGTGGCTGAGAAGGTTTCAGGAAACTTACAATCGTGGCAGAAGGGGAAG
>GX0540_concensus          TCACAATTCTGCGTGGCTGAGAAGGTTTCAGGAAACTTACAATCGTGGCAGAAGGGGAAG
HG03385_DEL2_hom           TCACAATTCTGCGTGGCTGAGGAGGTTTCAGGAAACTTACAATCGTGGCAGAAGGGGAAG        E-B
>GX0403-C_D2_concensus     TCACAATTCTGCGTGGCTGAGGAGGTTTCAGGAAACTTACAATCGTGGCAGAAGGGGAAG
>GX0300-C_D2_concensus     TCACAATTCTGCGTGGCTGAGGAGGTTTCAGGAAACTTACAATCGTGGCAGAAGGGGAAG
>GX0440-C_D2_concensus     TCACAATTCTGCGTGGCTGAGGAGGTTTCAGGAAACTTACAATCGTGGCAGAAGGGGAAG
                           ********************* **************************************


                                      B              X  X               XX
GYPE_F3-R3                 CAAACACATCGTTTTTCACATGATGGCAAAAGTAAATGGGGAAGCCCCTTATAAAACCAT        240
GYPE-GYPB_F3-R3            CAAACACATCCTTTTTCACATGATGGCAAAAGTAAATGGGGAAGCCCCTTATAAAACCAT        240
GYPB-GYPA_F3-R3            CAAACACATCCTTTTTCACATGATGACAGAAGTAAATGGGGAAGAGCCTTATAAAACCAT        240
GM19140_NORM_concensus     CAAACACATCCTTTTTCACATGATGACAGAAGTAAATGGGGAAGAGCCTTATAAAACCAT        B-A
>GX0387_concensus          CAAACACATCCTTTTTCACATGATGACAGAAGTAAATGGGGAAGAGCCTTATAAAACCAT
>GX0531_concensus          CAAACACATCCTTTTTCACATGATGACAGAAGTAAATGGGGAAGAGCCTTATAAAACCAT
>GX0540_concensus          CAAACACATCCTTTTTCACATGATGACAGAAGTAAATGGGGAAGAGCCTTATAAAACCAT
HG03385_DEL2_hom           CAAACACATCCTTTTTCACATGATGGCAAAAGTAAATGGGGAAGCCCCTTATAAAACCAT        E-B
>GX0403-C_D2_concensus     CAAACACATCCTTTTTCACATGATGGCAAAAGTAAATGGGGAAGCCCCTTATAAAACCAT
>GX0300-C_D2_concensus     CAAACACATCCTTTTTCACATGATGGCAAAAGTAAATGGGGAAGCCCCTTATAAAACCAT
>GX0440-C_D2_concensus     CAAACACATCCTTTTTCACATGATGGCAAAAGTAAATGGGGAAGCCCCTTATAAAACCAT
                           ********** ************** ** ***************  **************


                                X                                              X
GYPE_F3-R3                 CAGATCTCATGAGAATTTGCTCACTATCATGAAAATAGCATGGGGGAAACTGCCACAATG        300
GYPE-GYPB_F3-R3            CAGATTTCATGAGAATTTGCTCACTATCATGAAAATAGCATGGGGGAAACTGCCACAATG        300
GYPB-GYPA_F3-R3            CAGATCTCATGAGAATTTGCTCACTATCATGAAAATAGCATGGGGGAAACAGCCACAATG        300
GM19140_NORM_concensus     CAGATCTCATGAGAATTTGCTCACTATCATGAAAATAGCATGGGGGAAACAGCCACAATG        B-A
>GX0387_concensus          CAGATCTCATGAGAATTTGCTCACTATCATGAAAATAGCATGGGGGAAACAGCCACAATG
>GX0531_concensus          CAGATCTCATGAGAATTTGCTCACTATCATGAAAATAGCATGGGGGAAACAGCCACAATG
>GX0540_concensus          CAGATCTCATGAGAATTTGCTCACTATCATGAAAATAGCATGGGGGAAACAGCCACAATG
HG03385_DEL2_hom           CAGATTTCATGAGAATTTGCTCACTATCATGAAAATAGCATGGGGGAAACTGCCACAATG        E-B
>GX0403-C_D2_concensus     CAGATTTCATGAGAATTTGCTCACTATCATGAAAATAGCATGGGGGAAACTGCCACAATG
>GX0300-C_D2_concensus     CAGATTTCATGAGAATTTGCTCACTATCATGAAAATAGCATGGGGGAAACTGCCACAATG
>GX0440-C_D2_concensus     CAGATTTCATGAGAATTTGCTCACTATCATGAAAATAGCATGGGGGAAACTGCCACAATG
                           ***** ******************************************** *********
```

```
                                              B                   B
GYPE_F3-R3                    ATTCAATTACCTCCCACTACATTCCTCCCACAACACATGGGGATTGTGGGAACTACAATT      360
GYPE-GYPB_F3-R3               ATTCAATTACCTCCCACTACATTCCTCCCACAACACGTGGGGATTGTGGGAACTACAATT      360
GYPB-GYPA_F3-R3               ATTCAATTACCTCCCACTACATTCCTCCCACAACACGTGGGGATTGTGGGAACTACAATT      360
GM19140_NORM_concensus        ATTCAATTACCTCCCAYTACATTCCTCCCACAACACGTGGGGATTGTGGGAACTACAATT      B-A
>GX0387_concensus             ATTCAATTACCTCCCACTACATTCCTCCCACAACACGTGGGGATTGTGGGAACTACAATT
>GX0531_concensus             ATTCAATTACCTCCCACTACATTCCTCCCACAACACGTGGGGATTGTGGGAACTACAATT
>GX0540_concensus             ATTCAATTACCTCCCACTACATTCCTCCCACAACACGTGGGGATTGTGGGAACTACAATT
HG03385_DEL2_hom              ATTCAATTACCTCCCACTACATTCCTCCCACAACACGTGGGGATTGTGGGAACTACAATT      E-B
>GX0403-C_D2_concensus        ATTCAATTACCTCCCACTACATTCCTCCCACAACACGTGGGGATTGTGGGAACTACAATT
>GX0300-C_D2_concensus        ATTCAATTACCTCCCACTACATTCCTCCCACAACACGTGGGGATTGTGGGAACTACAATT
>GX0440-C_D2_concensus        ATTCAATTACCTCCCACTACATTCCTCCCACAACACGTGGGGATTGTGGGAACTACAATT
                              **************** ******************* ***********************
```

```
                                   X                                                    X
GYPE_F3-R3                    CAAGATGAGATTTGGGTGGGGACACAGCCAAACCACATCACTATGCCCCTGACCCCTCCC      420
GYPE-GYPB_F3-R3               CAAGAGGAGATTTGGGTGGGGACACAGCCAAACCACATCACTATGCCCCTGACCCCTCCC      420
GYPB-GYPA_F3-R3               CAAGATGAGATTTGGGTGGGGACACAGCCAAACCACATCACTATGCCCCTGACCCCTCGC      420
GM19140_NORM_concensus        CAAGATGAGATTTGGGTGGGGACACAGCCAAACCACATCACTATGCCCCTGACCCCTCGC      B-A
>GX0387_concensus             CAAGATGAGATTTGGGTGGGGACACAGCCAAACCACATCACTATGCCCCTGACCCCTCGC
>GX0531_concensus             CAAGATGAGATTTGGGTGGGGACACAGCCAAACCACATCACTATGCCCCTGACCCCTCGC
>GX0540_concensus             CAAGATGAGATTTGGGTGGGGACACAGCCAAACCACWTCACTATGCCCCTGACCCCTCGC
HG03385_DEL2_hom              CAAGAGGAGATTTGGGTGGGGACACAGCCAAACCACATCACTATGCCCCTGACCCCTCCC      E-B
>GX0403-C_D2_concensus        CAAGAGGAGATTTGGGTGGGGACACAGCCAAACCACATCACTATGCCCCTGACCCCTCCC
>GX0300-C_D2_concensus        CAAGAGGAGATTTGGGTGGGGACACAGCCAAACCACATCACTATGCCCCTGACCCCTCCC
>GX0440-C_D2_concensus        CAAGAGGAGATTTGGGTGGGGACACAGCCAAACCACATCACTATGCCCCTGACCCCTCCC
                              ***** **************************************************** *
```

```
                                                                             X
GYPE_F3-R3                    AAATCTCATGTCCTCACATTTCAAAACACAATCATGCCTTCCAAACAGTCCCCCAAAGTC      480
GYPE-GYPB_F3-R3               AAATCTCATGTCCTCACATTTCAAAACACAATCATGCCTTCCAAACAGTCCCCCAAAGTC      480
GYPB-GYPA_F3-R3               AAATCTCATGTCCTCACATTTCAAAACACAATCATGCCTTCCAAACGGTCCCCCAAAGTC      480
GM19140_NORM_concensus        AAATCTCATGTCCTCACATTTCAAAACACAATCATGCCTTCCAAACGGTCCCCCAAAGTC      B-A
>GX0387_concensus             AAATCTCATGTCCTCACATTTCAAAACACAATCATGCCTTCCAAACGGTCCCCCAAAGTC
>GX0531_concensus             AAATCTCATGTCCTCACATTTCAAAACACAATCATGCCTTCCAAACGGTCCCCCAAAGTC
>GX0540_concensus             AAATCTCATGTCCTCACATTTCAAAACACAATCATGCCTTCCAAACGGTCCCCCAAAGTC
HG03385_DEL2_hom              AAATCTCATGTCCTCACATTTCAAAACACAATCATGCCTTCCAAACAGTCCCCCAAAGTC      E-B
>GX0403-C_D2_concensus        AAATCTCATGTCCTCACATTTCAAAACACAATCATGCCTTCCAAACAGTCCCCCAAAGTC
>GX0300-C_D2_concensus        AAATCTCATGTCCTCACATTTCAAAACACAATCATGCCTTCCAAACAGTCCCCCAAAGTC
>GX0440-C_D2_concensus        AAATCTCATGTCCTCACATTTCAAAACACAATCATGCCTTCCAAACAGTCCCCCAAAGTC
                              *********************************************** ************
```

```
                               X         X                X         B
GYPE_F3-R3                    TAAACTCATTCCAGCATTAACTCAAAAGTCCAAGTCCAAAGTCTCATCTGAGACAAGGCA      540
GYPE-GYPB_F3-R3               TAAACTCATTCCAGCATTAACTCAAAAGTCCAAGTCCAAAGTCTCATCTGAGACAAGGCA      540
GYPB-GYPA_F3-R3               TTAACTCATTCTAGCATTAACTCAAAAGCCCAAGTCCAAAGTCTCATCTGAGACAAGGCA      540
GM19140_NORM_concensus        TTAACTCATTCTAGCATTAACTCAAAAGCCCAAGTCCAAAGTCTCATCTGAGACAAGGCA      B-A
>GX0387_concensus             TTAACTCATTCTAGCATTAACTCAAAAGCCCAAGTCCARAGTCTCATCTGAGACAAGGCA
>GX0531_concensus             TTAACTCATTCTAGCATTAACTCAAAAGCCCAAGTCCARAGTCTCATCTGAGACAAGGCA
>GX0540_concensus             TTAACTCATTCTAGCATTAACTCAAAAGCCCAAGTCCAAAGTCTCATCTGAGACAAGGCA
HG03385_DEL2_hom              TAAACTCATTCCAGCATTAACTCAAAAGTCCAAGTCCAAAGTCTCATCTGAGACAAGGCA      E-B
>GX0403-C_D2_concensus        TAAACTCATTCCAGCATTAACTCAAAAGTCCAAGTCCAAAGTCTCATCTGAGACAAGGCA
>GX0300-C_D2_concensus        TAAACTCATTCCAGCATTAACTCAAAAGTCCAAGTCCAAAGTCTCATCTGAGACAAGGCA
>GX0440-C_D2_concensus        TAAACTCATTCCAGCATTAACTCAAAAGTCCAAGTCCAAAGTCTCATCTGAGACAAGGCA
                              * ********* **************** *******************************
```

```
                               X                                      X
GYPE_F3-R3                  AGTCCCTTCCACCTATGAGCCTGTAAAATCAAAAGCAAGTTAGTTACTTCCTAGATACAA        600
GYPE-GYPB_F3-R3             AGTCCCTTCCACCTATGAGCCTGTAAAATCAAAAGCAAGTTAGTTACTTCCTAGATACAA        600
GYPB-GYPA_F3-R3             AGTACCTTCCACCTATGAGCCTGTAAAATCAAAAGCAAGTTAATTACTTCCTAGATACAA        600
GM19140_NORM_concensus      AGTACCTTCCACCTATGAGCCTGTAAAATCAAAAGCAAGTTAATTACTTCCTAGATACAA        B-A
>GX0387_concensus           AGTACCTTCCACCTATGAGCCTGTAAAATCAAAAGCAAGTTAATTACTTCCTAGATACAA
>GX0531_concensus           AGTACCTTCCACCTATGAGCCTGTAAAATCAAAAGCAAGTTAATTACTTCCTAGATACAA
>GX0540_concensus           AGTACCTTCCACCTATGAGCCTGTAAAATCAAAAGCAAGTTAATTACTTCCTAGATACAA
HG03385_DEL2_hom            AGTCCCTTCCACCTATGAGCCTGTAAAATCAAAAGCAAGTTAGTTACTTCCTAGATACAA        E-B
>GX0403-C_D2_concensus      AGTCCCTTCCACCTATGAGCCTGTAAAATCAAAAGCAAGTTAGTTACTTCCTAGATACAA
>GX0300-C_D2_concensus      AGTCCCTTCCACCTATGAGCCTGTAAAATCAAAAGCAAGTTAGTTACTTCCTAGATACAA
>GX0440-C_D2_concensus      AGTCCCTTCCACCTATGAGCCTGTAAAATCAAAAGCAAGTTAGTTACTTCCTAGATACAA
                            *** ************************************** *****************
```

```
                                                           X            X
GYPE_F3-R3                  TGAGGGTATAGGCAATGGGTAAATGCACCCATTCTAAATAGGATAAATTGGCCAAAAACA        660
GYPE-GYPB_F3-R3             TGAGGGTATAGGCAATGGGTAAATGCACCCACTCTAAATAGGAGCAATTGGCCAAAAACA        660
GYPB-GYPA_F3-R3             TGAGGGTATAGGCAATGGGTAAATGCACCCATTCTAAATAGGATAAATTGGCCAAAAACA        660
GM19140_NORM_concensus      TGAGGGTATAGGCAATGGGTAAATGCACCCATTCTAAATAGGATAAATTGGCCAAAAACA        B-A
>GX0387_concensus           TGAGGGTATAGGCAATGGGTAAATGCACCCATTCTAAATAGGATAAATTGGCCAAAAACA
>GX0531_concensus           TGAGGGTATAGGCAATGGGTAAATGCACCCATTCTAAATAGGATAAATTGGCCAAAAACA
>GX0540_concensus           TGAGGGTATAGGCAATGGGTAAATGCACCCATTCTAAATAGGATAAATTGGCCAAAAACA
HG03385_DEL2_hom            TGAGGGTATAGGCAATGGGTAAATGCACCCACTCTAAATAGGAGCAATTGGCCAAAAACA        E-B
>GX0403-C_D2_concensus      TGAGGGTATAGGCAATGGGTAAATGCACCCACTCTAAATAGGAGCAATTGGCCAAAAACA
>GX0300-C_D2_concensus      TGAGGGTATAGGCAATGGGTAAATGCACCCACTCTAAATAGGAGCAATTGGCCAAAAACA
>GX0440-C_D2_concensus      TGAGGGTATAGGCAATGGGTAAATGCACCCACTCTAAATAGGAGCAATTGGCCAAAAACA
                            ******************************* ***********  ***************
```

```
                              X                                 B                      B
GYPE_F3-R3                  GGAGCTACAGGCCCATGCAAGTCTGAAATCCAGTGGAGCAGTAATTAAATCTTAAAGCAT        720
GYPE-GYPB_F3-R3             GGGGCTACAGGCCCATGCAAGTCTGAAATCCAGTGGGGCAGTAATTAAATCTTAAAGCAC        720
GYPB-GYPA_F3-R3             GGAGCTACAGGCCCATGCAAGTCTGAAATCCAGTGGGGCAGTAATTAAATCTTAAAGCAC
GM19140_NORM_concensus      GGAGCTACAGGCCCATGCAAGTCTGAAATCCAGTGGGGCAGTAATTAAATCTTAAAGCAC        B-A
>GX0387_concensus           GGAGCTACAGGCCCATGCAAGTCTGAAATCCAGTGGGGCAGTAATTAAATCTTAAAGCAC
>GX0531_concensus           GGAGCTACAGGCCCATGCAAGTCTGAAATCCAGTGGGGCAGTAATTAAATCTTAAAGCAC
>GX0540_concensus           GGAGCTACAGGCCCATGCAAGTCTGAAATCCAGTGGGGCAGTAATTAAATCTTAAAGCAC
HG03385_DEL2_hom            GGGGCTACAGGCCCATGCAAGTCTGAAATCCAGTGGGGCAGTAATTAAATCTTAAAGCAC        E-B
>GX0403-C_D2_concensus      GGGGCTACAGGCCCATGCAAGTCTGAAATCCAGTGGGGCAGTAATTAAATCTTAAAGCAC
>GX0300-C_D2_concensus      GGGGCTACAGGCCCATGCAAGTCTGAAATCCAGTGGGGCAGTAATTAAATCTTAAAGCAC
>GX0440-C_D2_concensus      GGGGCTACAGGCCCATGCAAGTCTGAAATCCAGTGGGGCAGTAATTAAATCTTAAAGCAC
                            ** ********************************* ********************** 
```

```
                                           XX                    XX       B
                                                        DEL2_BP_seq_FWD =>
GYPE_F3-R3                  CTTAATAATCTCTTTTGACTCCATGTCTCACATCCAGGTAATGCTGTTGCAAGAGGTGGG        780
GYPE-GYPB_F3-R3             CTTAATAATCTCTTTGGACTCCATGTCTCACATCCAGTTAATGCTGATGCAAGAGGTGGG        780
GYPB-GYPA_F3-R3             CTTAATAATCTCTTTTAACTCCATGTCTCACATCCATGTAATGCTGATGCAAGAGGTGGG        780
GM19140_NORM_concensus      CTTAATAATCTCTTTTAACTCCATGTCTCACATCCATGTAATGCTGATGCAAGAGGTGGG        B-A
>GX0387_concensus           CTTAATAATCTCTTTTAACTCCATGTCTCACATCCATGTAATGCTGATGCAAGAGGTGGG
>GX0531_concensus           CTTAATAATCTCTTTTAACTCCATGTCTCACATCCATGTAATGCTGATGCAAGAGGTGGG
>GX0540_concensus           CTTAATAATCTCTTTTAACTCCATGTCTCACATCCATGTAATGCTGATGCAAGAGGTGGG
HG03385_DEL2_hom            CTTAATAATCTCTTTGGACTCCATGTCTCACATCCAGTTAATGCTGATGCAAGAGGTGGG        E-B
>GX0403-C_D2_concensus      CTTAATAATCTCTTTGGACTCCATGTCTCACATCCAGTTAATGCTGATGCAAGAGGTGGG
>GX0300-C_D2_concensus      CTTAATAATCTCTTTGGACTCCATGTCTCACATCCAGTTAATGCTGATGCAAGAGGTGGG
>GX0440-C_D2_concensus      CTTAATAATCTCTTTGGACTCCATGTCTCACATCCAGTTAATGCTGATGCAAGAGGTGGG
                            ***************  *******************  ******** *************
```

```
                            B                     X                  X   B    -B-- BB   BB
GYPE_F3-R3                  CTCCCACAGTCTTGGGAAGCTCAGCTCCTGTGGCTTTGCATGGTA-AACCCCCCCGCCTC--G     840
GYPE-GYPB_F3-R3             CTCCCACAGTCTTGGGAAGCTCCGCTCCTGTGGCTTTGCATAGTACAACC----CCGCTCTCG     839
GYPB-GYPA_F3-R3             TTCCCACAGTCTTGGGAAGCTCTGCTCCTGTGGCTTTGCATGGTACAACC----CCCCTCTCG     839
GM19140_NORM_concensus      CTCCCACAGTCTTGGGAAGCTCTGCTCCTGTGGCTTTGCATGGTACAACC----CCCCTCTCG     B-A
>GX0387_concensus           CTCCCACAGTCTTGGGAAGCTCTGCTCCTGTGGCTTTGCATGGTACAACC----CCCCTCTCG
>GX0531_concensus           CTCCCACAGTCTTGGGAAGCTCTGCTCCTGTGGCTTTGCATGGTACAACC----CCCCTCTCG
>GX0540_concensus           TTCCCACAGTCTTGGGAAGCTCTGCTCCTGTGGCTTTGCATGGTACAACC----CCCCTCTCG
HG03385_DEL2_hom            CTCCCACAGTCTTGGGAAGCTCCGCTCCTGTGGCTTTGCATAGTACAACC----CCCCTCTCG              E-B

>GX0403-C_D2_concensus      CTCCCACAGTCTTGGGAAGCTCCGCTCCTGTGGCTTTGCATAGTACAACC----CCCCTCTCG
>GX0300-C_D2_concensus      CTCCCACAGTCTTGGGAAGCTCCGCTCCTGTGGCTTTGCATAGTACAACC----CCCCTCTCG
>GX0440-C_D2_concensus      CTCCCACAGTCTTGGGAAGCTCCGCTCCTGTGGCTTTGCATAGTACAACC----CCCCTCTCG
                             ********************* ****************** *** * **    *  ***  *
                                                 --#1--                           --#2--
                                                     BsrBI restriction sites (CCGCTC)
                                Norm (GYPB-GYPA) not-cut        Norm (GYPB-GYPA)   not-cut
                                DEL2 (GYPE-GYPB)   cut          DEL2 (GYPE-GYPB)   cut(ref) not-cut(samples)
```

```
                                       X   -X--         X
GYPE_F3-R3                  GCTGCTTTCACAGGCTGTCTGTTATCTAGTTCCAAAGTCACTTCTGCATTTTTAGGTATC        900
GYPE-GYPB_F3-R3             GCTGCTTTCACAGGCTGTCTGTTATCCAGTTCCAAAGTCACTTCTGCATTTTTAGGTATC        899
GYPB-GYPA_F3-R3             GCTGCTTTCACGGGC----TGTTATCCAATTCCAAAGTCACTTCTGCATTTTTAGGTATC        895
GM19140_NORM_concensus      GCTGCTTTCACGGGC----TGTTATCCAATTCCAAAGTCACTTCTGCATTTTTAGGTATC        B-A
>GX0387_concensus           GCTGCTTTCACGGGC----TGTTATCCAATTCCAAAGTCACTTCTGCATTTTTAGGTATC
>GX0531_concensus           GCTGCTTTCACGGGC----TGTTATCCAATTCCAAAGTCACTTCTGCATTTTTAGGTATC
>GX0540_concensus           GCTGCTTTCACGGGC----TGTTATCCAATTCCAAAGTCACTTCTGCATTTTTAGGTATC
HG03385_DEL2_hom            GCTGCTTTCACAGGCTGTCTGTTATCCAGTTCCAAAGTCACTTCTGCATTTTTAGGTATC        E-B
>GX0403-C_D2_concensus      GCTGCTTTCACAGGCTGTCTGTTATCCAGTTCCAAAGTCACTTCTGCATTTTTAGGTATC
>GX0300-C_D2_concensus      GCTGCTTTCACAGGCTGTCTGTTATCCAGTTCCAAAGTCACTTCTGCATTTTTAGGTATC
>GX0440-C_D2_concensus      GCTGCTTTCACAGGCTGTCTGTTATCCAGTTCCAAAGTCACTTCTGCATTTTTAGGTATC
                            *********** ***    ******* * *******************************
```

```
                                                                             X        B
GYPE_F3-R3                  CTTATAGCAGCACCCCACCTCTAGTACCAACTTACTGTATTAGTCTGTTCTCATGCTGTT        960
GYPE-GYPB_F3-R3             CTTATAGCAGCACCCCACCTCTAGTACCAACTTACTGTATTAGTCTGTTCTCATGCTGCT        959
GYPB-GYPA_F3-R3             CTTATAGCAGCACCCCACCTCTAGTACCAACTTACTGTATTAGTCTGTC-TCATGCTGCT        954
GM19140_NORM_concensus      CTTATAGCAGCACCCCACCTCTAGTACCAACTTACTGTATTAGTCTGTC-TCATGCTGCT        B-A
>GX0387_concensus           CTTATAGCAGCACCCCACCTCTRGTACCAACTTACTGTATTAGTCTGTC-TCATGCTGCT
>GX0531_concensus           CTTATAGCAGCACCCCACCTCTRGTACCAACTTACTGTATTAGTCTGTC-TCATGCTGCT
>GX0540_concensus           CTTATAGCAGCACCCCACCTCTAGTACCAACTTACTGTATTAGTCTGTC-TCATGCTGCT
HG03385_DEL2_hom            CTTATAGCAGCACCCCACCTCTAGTACCAACTTACTGTATTAGTCTGTTCTCATGCTGCT        E-B
>GX0403-C_D2_concensus      CTTATAGCAGCACCCCACCTCTAGTACCAACTTACTGTATTAGTCTGTTCTCATGCTGCT
>GX0300-C_D2_concensus      CTTATAGCAGCACCCCACCTCTAGTACCAACTTACTGTATTAGTCTGTTCTCATGCTGCT
>GX0440-C_D2_concensus      CTTATAGCAGCACCCCACCTCTAGTACCAACTTACTGTATTAGTCTGTTCTCATGCTGCT
                            ************************************************  ******** *
```

```
                                      X B                     --X--       XX
GYPE_F3-R3                  ATAAAAAACTGCCCAAGACTGTGTAATTTATAAAGGAAAGAGGTTTTATTGATCTACAGT        1020
GYPE-GYPB_F3-R3             ATAAAAAACTGCCCAAGACTGTGTAATTTATAAAGGAAAGAGGTTTAGTTGATCTACAGT        1019
GYPB-GYPA_F3-R3             ATAAAAAACTTCCCAAGACTGTGTAATTTATAAA-----GAGGTTTAATTGATCTACAGT        1009
GM19140_NORM_concensus      ATAAAAAACTTCCCAAGACTGTGTAATTTATAAA-----GAGGTTTAATTGATCTACAGT        B-A
>GX0387_concensus           ATAAAAAACTTCCCAAGACTGTGTAATTTATAAA-----GAGGTTTAATTGATCTACAGT
>GX0531_concensus           ATAAAAAACTTCCCAAGACTGTGTAATTTATAAA-----GAGGTTTAATTGATYTACAGT
>GX0540_concensus           ATAAAAAACTTCCCAAGACTGTGTAATTTATAAA-----GAGGTTTAATTGATCTACAGT
HG03385_DEL2_hom            ATAAAAAACTGCGCAAGACTGTGTAATTTATAAAGGAAAGAGGTTTAGTTGATCTACAGT        E-B
>GX0403-C_D2_concensus      ATAAAAAACTGCGCAAGACTGTGTAATTTATAAAGGAAAGAGGTTTAGTTGATCTACAGT
>GX0300-C_D2_concensus      ATAAAAAACTGCGCAAGACTGTGTAATTTATAAAGGAAAGAGGTTTAGTTGATCTACAGT
>GX0440-C_D2_concensus      ATAAAAAACTGCGCAAGACTGTGTAATTTATAAAGGAAAGAGGTTTAGTTGATCTACAGT
                            ********** * *********************     *******  ************
```

```
                                              -----Z------
                                              X         BX
GYPE_F3-R3                   TTTGCATGGCTGGGAAGGTCT-CAGGATACTTACAATCATGACCAAAGGGGAAACAAACA       1079
GYPE-GYPB_F3-R3              TTTGCATGGCTTGGAAGGTCT-TAGGATACTTACAATCATGACCAAAGGGGAAACAAACA       1078
GYPB-GYPA_F3-R3              TTTGCATGGCTGGGAAGGTCT-CAGGATACTTACAATCATGACCAAAGGGGAAACAAACA       1068
GM19140_NORM_concensus       TTTGCATGGCTGGGAAGGTCTTCAGGATACTTACAATCATGACCAAAGGGGAAACAAACA       B-A
>GX0387_concensus            TTTGCATGGCTGGGAAGGTCT-CAGGATACTTACAATCATGACCAAAGGGGAAACAAACA
>GX0531_concensus            TTTGCATGGCTGGGAAGGTCT-CAGGATACTTACAATCATGACCAAAGGGGAAACAAACA
>GX0540_concensus            TTTGCATGGCTGGGAAGGTCT-CAGGATACTTACAATCATGACCAAAGGGGAAACAAACA
HG03385_DEL2_hom             TTTGCATGGCTTGGAAGGTCT-TAGAATACTTACAATCATGACCAAAGGGGAAACAAACA       E-B
>GX0403-C_D2_concensus       TTTGCATGGCTTGGAAGGTCT-TAGAATACTTACAATCATGACCAAAGGGGAAACAAACA
>GX0300-C_D2_concensus       TTTGCATGGCTTGGAAGGTCT-TAGAATACTTACAATCATGACCAAAGGGGAAACAAACA
>GX0440-C_D2_concensus       TTTGCATGGCTTGGAAGGTCT-TAGAATACTTACAATCATGACCAAAGGGGAAACAAACA
                             *********** *********  *************************************


GYPE_F3-R3                   CATCTTTCTTACATAGTGGCAGGAAGGAGAAGAATGAGAGCTGAGTGAAGGGGGAAGCTC       1139
GYPE-GYPB_F3-R3              CATCTTTCTTACATAGTGGCAGGAAGGAGAAGAATGAGAGCTGAGTGAAGGGGGAAGCTC       1138
GYPB-GYPA_F3-R3              CATCTTTCTTACATAGTGGCAGGAAGGAGAAGAATGAGAGCTGAGTGAAGGGGGAAGCTC       1128
GM19140_NORM_concensus       CATCTTTCTTACATAGTGGCAGGAAGGAGAAGAATGAGAGCTGAGTGAAGGGGGAAGCTC
>GX0387_concensus            CATCTTTCTTACATAGTGGCAGGAAGGAGAAGAATGAGAGCTGAGTGAAGGGGGAAGCTC
>GX0531_concensus            CATCTTTCTTACATAGTGGCAGGAAGGAGAAGAATGAGAGCTGAGTGAAGGGGGAAGCTC
>GX0540_concensus            CATCTTTCTTACATAGTGGCAGGAAGGAGAAGAATGAGAGCTGAGTGAAGGGGGAAGCTC
HG03385_DEL2_hom             CATCTTTCTTACATAGTGGCAGGAAGGAGAAGAATGAGAGCTGAGTGAAGGGGGAAGCTC
>GX0403-C_D2_concensus       CATCTTTCTTACATAGTGGCAGGAAGGAGAAGAATGAGAGCTGAGTGAAGGGGGAAGCTC
>GX0300-C_D2_concensus       CATCTTTCTTACATAGTGGCAGGAAGGAGAAGAATGAGAGCTGAGTGAAGGGGGAAGCTC
>GX0440-C_D2_concensus       CATCTTTCTTACATAGTGGCAGGAAGGAGAAGAATGAGAGCTGAGTGAAGGGGGAAGCTC
                             ************************************************************


                                                                  ---------ZZ----------
                                                                  X     X              X
GYPE_F3-R3                   CTTTATAAAACTATCAGATTATGTGAGAATTTACTCACTATCATGAGAATAGCACAGGGG       1199
GYPE-GYPB_F3-R3              CTTTATAAAACTATCAGATTATGTGAGAATTTACTCACTATCATGAGAATAGCACAGGGG       1198
GYPB-GYPA_F3-R3              CTTTATAAAACTATCAGATTATGTGAGAATTTATTCACTCTCATGAGAATAGCATAGGGG       1188
GM19140_NORM_concensus       CTTTATAAAACTATCAGATTATGTGAGAATTTATTCACTCTCATGAGAATAGCATAGGGG       B-A
>GX0387_concensus            CTTTATAAAACTATCAGATTATGTGAGAATTTATTCACTCTCATGAGAATAGCATAGGGG
>GX0531_concensus            CTTTATAAAACTATCAGATTATGTGAGAATTTATTCACTCTCATGAGAATAGCATAGGGG
>GX0540_concensus            CTTTATAAAACTATCAGATTATGTGAGAATTTATTCACTCTCATGAGAATAGCATAGGGG
HG03385_DEL2_hom             CTTTATAAAACTATCAGATTATGTGAGAATTTATTCACTCTCATGAGAATAGCATAGGGG       B-A
>GX0403-C_D2_concensus       CTTTATAAAACTATCAGATTATGTGAGAATTTATTCACTCTCATGAGAATAGCATAGGGG
>GX0300-C_D2_concensus       CTTTATAAAACTATCAGATTATGTGAGAATTTATTCACTCTCATGAGAATAGCATAGGGG
>GX0440-C_D2_concensus       CTTTATAAAACTATCAGATTATGTGAGAATTTATTCACTCTCATGAGAATAGCATAGGGG
                             ********************************* ***** ************** *****


                                                                                 X
GYPE_F3-R3                   AAACCACCGCAATGATTCAAGTACCTCCCACTGGGTTCCTCCCATGACATGTGGGGATTA       1259
GYPE-GYPB_F3-R3              AAACCACCGCAATGATTCAAGTACCTCCCACTGGGTTCCTCCCATGACATGTGGGGATTA       1258
GYPB-GYPA_F3-R3              AAACCACCGCAATGATTCAAGTACCTCCCACTGGGTTCCTCCCATGACACGTGGGGATTA       1248
GM19140_NORM_concensus       AAACCACCGCAATGATTCAAGTACCTCCCACTGGGTTCCTCCCATGACACGTGGGGATTA       B-A
>GX0387_concensus            AAACCACCGCAATGATTCAAGTACCTCCCACTGGGTTCCTCCCATGACACGTGGGGATTA
>GX0531_concensus            AAACCACCGCAATGATTCAAGTACCTCCCACTGGGTTCCTCCCATGACACGTGGGGATTA
>GX0540_concensus            AAACCACCGCAATGATTCAAGTACCTCCCACTGGGTTCCTCCCATGACACGTGGGGATTA
HG03385_DEL2_hom             AAACCACCGCAATGATTCAAGTACCTCCCACTGGGTTCCTCCCATGACACGTGGGGATTA       B-A
>GX0403-C_D2_concensus       AAACCACCGCAATGATTCAAGTACCTCCCACTGGGTTCCTCCCATGACACGTGGGGATTA
>GX0300-C_D2_concensus       AAACCACCGCAATGATTCAAGTACCTCCCACTGGGTTCCTCCCATGACACGTGGGGATTA
>GX0440-C_D2_concensus       AAACCACCGCAATGATTCAAGTACCTCCCACTGGGTTCCTCCCATGACACGTGGGGATTA
                             ************************************************* **********
```

```
                                                                      X          X
GYPE_F3-R3                    TTGGAACTACAATTCAAGATGAGATTTGAGTGGGAACACAGCCAAACCATATCAGTCATT    1319
GYPE-GYPB_F3-R3               TTGGAACTACAATTCAAGATGAGATTTGAGTGGGAACACAGTCAAACCATATTAGTCATT    1318
GYPB-GYPA_F3-R3               TTGGAACTACAATTCAAGATGAGATTTGAGTGGGAACACAGCCAAACCATATCAGTCATT    1308
GM19140_NORM_concensus        TTGGAACTACAATTCAAGATGAGATTTGAGTGGGAACACAGCCAAACCATATCAGTCATT    B-A
>GX0387_concensus             TTGGAACTACAATTCAAGATGAGATTTGAGTGGGAACACAGCCAAACCATATCAGTCATT
>GX0531_concensus             TTGGAACTACAATTCAAGATGAGATTTGAGTGGGAACACAGCCAAACCATATCAGTCATT
>GX0540_concensus             TTGGAACTACAATTCAAGATGAGATTTGAGTGGGAACACAGCCAAACCATATCAGTCATT
HG03385_DEL2_hom              TTGGAACTACAATTCAAGATGAGATTTGAGTGGGAACACAGCCAAACCATATCAGTCATT    B-A
>GX0403-C_D2_concensus        TTGGAACTACAATTCAAGATGAGATTTGAGTGGGAACACAGCCAAACCATATCAGTCATT
>GX0300-C_D2_concensus        TTGGAACTACAATTCAAGATGAGATTTGAGTGGGAACACAGCCAAACCATATCAGTCATT
>GX0440-C_D2_concensus        TTGGAACTACAATTCAAGATGAGATTTGAGTGGGAACACAGCCAAACCATATCAGTCATT
                              ***************************************** ********** *******


                                                              B                       XX
                                                                     <=  DEL2_BP_seq_Rev1
GYPE_F3-R3                    CCACATATTGAGTGATTTCTCTCTTCTATTCAGTATTCTTCCACAGAGAGGGACCCATAG    1379
GYPE-GYPB_F3-R3               CCACATATTGAGTGATTTCTCTCTTCTATTCACTATTCTTCCACAGAGAGGGACCCATAG    1378
GYPB-GYPA_F3-R3               CCACATATTGAGTGATTTCTCTCTTCTATTCACTATTCTTCCACAGAGAGGGACCTGTAG    1368
GM19140_NORM_concensus        CCACATATTGAGTGATTTCTCTCTTCTATTCACTATTCTTCCACAGAGAGGGACCTGTAG    B-A
>GX0387_concensus             CCACATATTGAGTGATTTCTCTCTTCTATTCACTATTCTTCCACAGAGAGGGACCTGTAG
>GX0531_concensus             CCACATATTGAGTGATTTCTCTCTTCTATTCACTATTCTTCCACAGAGAGGGACCTGTAG
>GX0540_concensus             CCACATATTGAGTGATTTCTCTCTTCTATTCACTATTCTTCCACAGAGAGGGACCTGTAG
HG03385_DEL2_hom              CCACATATTGAGTGATTTCTCTCTTCTATTCACTATTCTTCCACAGAGAGGGACCTGTAG    B-A
>GX0403-C_D2_concensus        CCACATATTGAGTGATTTCTCTCTTCTATTCACTATTCTTCCACAGAGAGGGACCTGTAG
>GX0300-C_D2_concensus        CCACATATTGAGTGATTTCTCTCTTCTATTCACTATTCTTCCACAGAGAGGGACCTGTAG
>GX0440-C_D2_concensus        CCACATATTGAGTGATTTCTCTCTTCTATTCACTATTCTTCCACAGAGAGGGACCTGTAG
                              ******************************** ***********************  ***


                                                           XX B     X    B      XX
GYPE_F3-R3                    TCATTACCTTCAAGGAACCTAAATCCTGGTGTTTTTATGGTTACGGGTGGCATATAATAT    1439
GYPE-GYPB_F3-R3               TCATTACCTTCAAGGAACCTAAATCCTGGCATCTTTATTGTTATGGGTGGTGTATAATAT    1438
GYPB-GYPA_F3-R3               TCATTACCTTCAAGGAACCTAAATCCTGGTGTCTTTATGGTTATGGGTGGCATATAATAT    1428
GM19140_NORM_concensus        TCATTACCTTCAAGGAACCTAAATCCTGGTGTCTTTATGGTTATGGGTGGCATATAATAT    B-A
>GX0387_concensus             TCATTACCTTCAAGGAACCTAAATCCTGGTGTCTTTATGGTTATGGGTGGCATATAATAT
>GX0531_concensus             TCATTACCTTCAAGGAACCTAAATCCTGGTGTCTTTATGGTTATGGGTGGCATATAATAT
>GX0540_concensus             TCATTACCTTCAAGGAACCTAAATCCTGGTGTCTTTATGGTTATGGGTGGCATATAATAT
HG03385_DEL2_hom              TCATTACCTTCAAGGAACCTAAATCCTGGTGTCTTTATGGTTATGGGTGGCATATAATAT    B-A
>GX0403-C_D2_concensus        TCATTACCTTCAAGGAACCTAAATCCTGGTGTCTTTATGGTTATGGGTGGCATATAATAT
>GX0300-C_D2_concensus        TCATTACCTTCAAGGAACCTAAATCCTGGTGTCTTTATGGTTATGGGTGGCATATAATAT
>GX0440-C_D2_concensus        TCATTACCTTCAAGGAACCTAAATCCTGGTGTCTTTATGGTTATGGGTGGCATATAATAT
                              *****************************  * ***** **** ******  ********


                                  B                         BB         A A        B
GYPE_F3-R3                    ACAGGTAAGCCAATGTTACAGGATGTTGTGGAACTATTCCTATTCTTCCATAAGTAGCCA    1499
GYPE-GYPB_F3-R3               ACAGGTAAGCCAATGTTACAGGATGTTGTGAGACTATTCCTATCCTTCCATAATTAGCCA    1498
GYPB-GYPA_F3-R3               ACAGGTAAGCCAATGTTACAGGATGTTGTGAGACTATTCCTGTTCTTCCATAATTAGCCA    1488
GM19140_NORM_concensus        ACAGGTAAGCCAATGTTACAGGATGTTGTGAGACTATTCCTGTTCTTCCATAATTAGCCA    B-A
>GX0387_concensus             ACAGGTAAGCCAATGTTACAGGATGTTGTGAGACTATTCCYGTTCTTCCATAATTAGCCA
>GX0531_concensus             ACAGGTAAGCCAATGTTACAGGATGTTGTGAGACTATTCCYGTTCTTCCATAATTAGCCA
>GX0540_concensus             ACAGGTAAGCCAATGTTACAGGATGTTGTGAGACTATTCCTGTTCTTCCATAATTAGCCA
HG03385_DEL2_hom              ACAGATAAGCCAATGTTACAGGATGTTGTGAGACTATTCCTGTTCTTCCATAATTAGCCA    B-A
>GX0403-C_D2_concensus        ACAGATAAGCCAATGTTACAGGATGTTGTGAGACTATTCCTGTTCTTCCATAATTAGCCA
>GX0300-C_D2_concensus        ACAGATAAGCCAATGTTACAGGATGTTGTGAGACTATTCCTGTTCTTCCATAATTAGCCA
>GX0440-C_D2_concensus        ACAGATAAGCCAATGTTACAGGATGTTGTGAGACTATTCCTGTTCTTCCATAATTAGCCA
                              **** *************************  ********* * ********* ******


GYPE_F3-R3                    CTAGGACCACGGCTGAAACCAAGAGGCACTGATTACCACCTCACTCTAAAGAGAATTACT    1559
GYPE-GYPB_F3-R3               CTAGGACCACAGCTGAAACCAAGAGGCACTGATTACCCCCTCACTCTAAAGAGAATTACT    1558
GYPB-GYPA_F3-R3               CTAGGACCACGGCTGAAACCAAGAGGCACTGATTACCGCCTCACTCTAAAGAGAATTACT    1548
GM19140_NORM_concensus        CTAGGACCACGGCTGAAACCAAGAGGCACTGATTACCGCCTCACTCTAAAGAGAATTACT    B-A
>GX0387_concensus             CTAGGACCACGGCTGAAACCAAGAGGCACTGATTACCGCCTCACTCTAAAGAGAATTACT
>GX0531_concensus             CTAGGACCACGGCTGAAACCAAGAGGCACTGATTACCGCCTCACTCTAAAGAGAATTACT
>GX0540_concensus             CTAGGACCACGGCTGAAACCAAGAGGCACTGATTACCGCCTCACTCTAAAGAGAATTACT
HG03385_DEL2_hom              CTAGGACCACGGCTGAAACCAAGAGGCACTGATTACCGCCTCACTCTAAAGAGAATTACT    B-A
>GX0403-C_D2_concensus        CTAGGACCACGGCTGAAACCAAGAGGCACTGATTACCGCCTCACTCTAAAGAGAATTACT
>GX0300-C_D2_concensus        CTAGGACCACGGCTGAAACCAAGAGGCACTGATTACCGCCTCACTCTAAAGAGAATTACT
```

```
>GX0440-C_D2_concensus        CTAGGACCACGGCTGAAACCAAGAGGCACTGATTACCGCCTCACTCTAAAGAGAATTACT
                              ********** ************************** **********************

                                 X     B       X            X                X
GYPE_F3-R3                    GAGTTTGTGTTCACTGCTGGGGCCAGATCTCTTTGCAGGGCTATGTTAGGCTTTACACAA    1619
GYPE-GYPB_F3-R3               GAGCTTGTGCTCACTGCGGGGGCCAGATCTTTTTGCAGGGCTATGTTAGGCTTTACACAA    1618
GYPB-GYPA_F3-R3               GAGTTTGTGCTCACTGCTGGGGCCAGATCTCTTTGCAGGGCTATGTTTGGCTTTACACAA    1608
GM19140_NORM_concensus        GAGTTTGTGCTCACTGCTGGGGCCAGATCTCTTTGCAGGGCTATGTTTGGCTTTACACAA    B-A
>GX0387_concensus             GAGTTTGTGCTCACTGCTGGGGCCAGATCTCTTTGCAGGGCTATGTTTGGCTTTACACAA
>GX0531_concensus             GAGTTTGTGCTCACTGCTGGGGCCAGATCTCTTTGCAGGGCTATGTTTGGCTTTACACAA
>GX0540_concensus             GAGTTTGTGCTCACTGCTGGGGCCAGATCTCTTTGCAGGGCTATGTTTGGCTTTACACAA
HG03385_DEL2_hom              GAGTTTGTGCTCACTGCTGGGGCCAGATCTCTTTGCAGGGCTATGTTTGGCTTTACACAA    B-A
>GX0403-C_D2_concensus        GAGTTTGTGCTCACTGCTGGGGCCAGATCTCTTTGCAGGGCTATGTTTGGCTTTACACAA
>GX0300-C_D2_concensus        GAGTTTGTGCTCACTGCTGGGGCCAGATCTCTTTGCAGGGCTATGTTTGGCTTTACACAA
>GX0440-C_D2_concensus        GAGTTTGTGCTCACTGCTGGGGCCAGATCTCTTTGCAGGGCTATGTTTGGCTTTACACAA
                              *** ***** ******* ************ **************** ************

                                                                        X   B
GYPE_F3-R3                    TGTGACCCTCTAGTCTGTACAAGGAGAGGAATGCAGAGGGAAAGCGATTCACTGGAGTCT    1679
GYPE-GYPB_F3-R3               TGTGACCCTCTAGTCTGTACAAGGAGAGGAATGCAGAGGGGAAGAGATTCACTGGAGTCT    1678
GYPB-GYPA_F3-R3               TGTGACCCTCTAGTCTGTACAAGGAGAGGAATGCAGAGGGAAAGAGATTCACTGGAGTCT    1668
GM19140_NORM_concensus        TGTGACCCTCTAGTCTGTACAAGGAGAGGAATGCAGAGGGAAAGAGATTCACTGGAGTCT    B-A
>GX0387_concensus             TGTGACCCTCTAGTCTGTACAAGGAGAGGAATGCAGAGGGAAAGAGATTCACTGGAGTCT
>GX0531_concensus             TGTGACCCTCTAGTCTGTACAAGGAGAGGAATGCAGAGGGAAAGAGATTCACTGGAGTCT
>GX0540_concensus             TGTGACCCTCTAGTCTGTACAAGGAGAGGAATGCAGAGGGAAAGAGATTCACTGGAGTCT
HG03385_DEL2_hom              TGTGACCCTCTAGTCTGTACAAGGAGAGGAATGCAGAGGGAAAGAGATTCACTGGAGTCT    B-A
>GX0403-C_D2_concensus        TGTGACCCTCTAGTCTGTACAAGGAGAGGAATGCAGAGGGAAAGAGATTCACTGGAGTCT
>GX0300-C_D2_concensus        TGTGACCCTCTAGTCTGTACAAGGAGAGGAATGCAGAGGGAAAGAGATTCACTGGAGTCT
>GX0440-C_D2_concensus        TGTGACCCTCTAGTCTGTACAAGGAGAGGAATGCAGAGGGAAAGAGATTCACTGGAGTCT
                              **************************************** *** ***************

                                                                                    K
GYPE_F3-R3                    CTCGTCAGTGACCTGGTGGTGAATATATGGTAATGGGTAAATCCCACTTCACCTAATGAA    1739
GYPE-GYPB_F3-R3               CTCGTCAGTGACCTGGTGGTGAATATATGGTAATGGGTAAATCCCACTTCACCTGATGAA    1738
GYPB-GYPA_F3-R3               CTCGTCAGTGACCTGGTGGTGAATATATGGTAATGGGTAAATCCCACTTCACCTGATGAA    1728
GM19140_NORM_concensus        CTCGTCAGTGACCTGGTGGTGAATATATGGTAATGGGTAAATCCCACTTCACCTGATGAA    B-A
>GX0387_concensus             CTCGTCAGTGACCTGGTGGTGAATATATGGTAATGGGTAAATCCCACTTCACCTGATGAA
>GX0531_concensus             CTCGTCAGTGACCTGGTGGTGAATATATGGTAATGGGTAAATCCCACTTCACCTGATGAA
>GX0540_concensus             CTCGTCAGTGACCTGGTGGTGAATATATGGTAATGGGTAAATCCCACTTCACCTGATGAA
HG03385_DEL2_hom              CTCGTCAGTGACCTGGTGGTGAATATATGGTAATGGGTAAATCCCACTTCACCTGATGAA    B-A
>GX0403-C_D2_concensus        CTCGTCAGTGACCTGGTGGTGAATATATGGTAATGGGTAAATCCCACTTCACCTGATGAA
>GX0300-C_D2_concensus        CTCGTCAGTGACCTGGTGGTGAATATATGGTAATGGGTAAATCCCACTTCACCTGATGAA
>GX0440-C_D2_concensus        CTCGTCAGTGACCTGGTGGTGAATATATGGTAATGGGTAAATCCCACTTCACCTGATGAA
                              ****************************************************** *****

                                                                           X            X
GYPE_F3-R3                    GAAAAGGAGAAGGATGCACTTCATTCTTAGAATCTAAGCAGGAACAGGAAAGGGAAAATC    1799
GYPE-GYPB_F3-R3               GAAAAGGAGAAGGATGCACTTCATTCTTAGAATCTAAGCAGGAACGGGAAAGGGAAAATT    1798
GYPB-GYPA_F3-R3               GAAAAGGAGAAGGATGCACTTCATTCTTAGAATCTAAGCAGGAACAGGAAAGGGAAAATC    1788
GM19140_NORM_concensus        GAAAAGGAGAAGGATGCACTTCATTCTTAGAATCTAAGCAGGAACAGGAAAGGGAAAATC    B-A
>GX0387_concensus             GAAAAGGAGAAGGATGCACTTCATTCTTAGAATCTAAGCAGGAACAGGAAAGGGAAAATC
>GX0531_concensus             GAAAAGGAGAAGGATGCACTTCATTCTTAGAATCTAAGCAGGAACAGGAAAGGGAAAATC
>GX0540_concensus             GAAAAGGAGAAGGATGCACTTCATTCTTAGAATCTAAGCAGGAACAGGAAAGGGAAAATC
HG03385_DEL2_hom              GAAAAGGAGAAGGATGCACTTCATTCTTAGAATCTAAGCAGGAACAGGAAAGGGAAAATC    B-A
>GX0403-C_D2_concensus        GAAAAGGAGAAGGATGCACTTCATTCTTAGAATCTAAGCAGGAACAGGAAAGGGAAAATC
>GX0300-C_D2_concensus        GAAAAGGAGAAGGATGCACTTCATTCTTAGAATCTAAGCAGGAACAGGAAAGGGAAAATC
>GX0440-C_D2_concensus        GAAAAGGAGAAGGATGCACTTCATTCTTAGAATCTAAGCAGGAACAGGAAAGGGAAAATC
                              ********************************************* *************

                              X                      XX     X
GYPE_F3-R3                    TAGGAGAAAAGCTTACAAAGACTGTAAATGCCTTCAAGAAGGTGAGAGAAACTAATATTT    1859
GYPE-GYPB_F3-R3               -AGGAGAAAAGCTTACAAAGACTGTAAATGCCTTCAAGAAGGTGAGAGAAACTAATATTT    1857
GYPB-GYPA_F3-R3               TAGGAGAAAAGCTTACAAAGACT--AAATGTCTTCAAGAAGGTGAGAGAAACTAATATTT    1846
GM19140_NORM_concensus        TAGGAGAAAAGCTTACAAAGACT--AAATGTCTTCAAGAAGGTGAGAGAAACTAATATTT    B-A
>GX0387_concensus             TAGGAGAAAAGCTTACAAAGACT--AAATGTCTTCAAGAAGGTGAGAGAAACTAATATTT
>GX0531_concensus             TAGGAGAAAAGCTTACAAAGACT--AAATGTCTTCAAGAAGGTGAGAGAAACTAATATTT
>GX0540_concensus             TAGGAGAAAAGCTTACAAAGACT--AAATGTCTTCAAGAAGGTGAGAGAAACTAATATTT
HG03385_DEL2_hom              TAGGAGAAAAGCTTACAAAGACT--AAATGTCTTCAAGAAGGTGAGAGAAACTAATATTT    B-A
>GX0403-C_D2_concensus        TAGGAGAAAAGCTTACAAAGACT--AAATGTCTTCAAGAAGGTGAGAGAAACTAATATTT
>GX0300-C_D2_concensus        TAGGAGAAAAGCTTACAAAGACT--AAATGTCTTCAAGAAGGTGAGAGAAACTAATATTT
>GX0440-C_D2_concensus        TAGGAGAAAAGCTTACAAAGACT--AAATGTCTTCAAGAAGGTGAGAGAAACTAATATTT
                               **********************  ***** *****************************
```

```
                                            X     X    B                          ---B--
GYPE_F3-R3                  GGTTTACTGTCAGCAGTCTGTTCGGGCGGCATTTTGCCAATAT-AGCCCTATCC------       1910
GYPE-GYPB_F3-R3             GGTTTACTGTCAGCAGGCTGTTCGGGCGGCATTTTGCCAATAT-AGCCCTATCCCTACGT       1916
GYPB-GYPA_F3-R3             GGTTTACTGTCAGCAGGCTGTTTGGGCGGCATTTTGCCAATATTAGCCCTATCCCTACGT       1906
GM19140_NORM_concensus      GGTTTACTGTCAGCAGGCTGTTTGGGCGGCATTTTGCCAATATTAGCCCTATCCCTACGT       B-A
>GX0387_concensus           GGTTTACTGTCAGCAGGCTGTTTGGGCGGCATTTTGCCAA-AATAACCCTATCCCTACGT
>GX0531_concensus           GGTTTACTGTCAGCAGGCTGTTTGGGCRGCATTTTGCCAATATTAGCCCTATCCCTACGT
>GX0540_concensus           GGTTTACTGTCAGCAGGCTGTTTGGGCGGCATTTTGCCAATATTAGCCCTATCCCTACGT
HG03385_DEL2_hom            GGTTTACTGTCAGCAGGCTGTTTGGGCGGCATTTTGCCAATATTAGCCCTATCCCTACGT       B-A
>GX0403-C_D2_concensus      GGTTTACTGTCAGCAGGCTGTTTGGGCGGCATTTTGCCAATATTAGCCCTATCCCTACGT
>GX0300-C_D2_concensus      GGTTTACTGTCAGCAGGCTGTTTGGGCGGCATTTTGCCAATATTAGCCCTATCCCTACGT
>GX0440-C_D2_concensus      GGTTTACTGTCAGCAGGCTGTTTGGGCGGCATTTTGCCAATATTAGCCCTATCCCTACGT
                            **************** ***** ****************   ** **** *****


                            -B-  X   X                      X
GYPE_F3-R3                  ---TAAGGGAAAGGGAATTGTGGTTGGACAACCGAGTTCTGAGAAAGGCAGTGATTGAGC       1969
GYPE-GYPB_F3-R3             GAGTA-GGGGAAGGGAATTGTGGTTGGACAACAGAGTTCTGAGAAAGGCAGTGATTGAGC       1975
GYPB-GYPA_F3-R3             GAGTAGGGGAAAGGGAATTGTGGTTGGACAACCGAGTTCTGAGAAAGGCAGTGATTGAGC       1966
GM19140_NORM_concensus      GAATAGGGGAAAGGGAATTGTGGTTGGACAACCGAGTTCTGAGAAAGGCAGTGATTGAGC       B-A
>GX0387_concensus           GAGTAGGGGAAAGGGAATTGTGGTTGGACAACCGAGTTCTGAGAAAGGCAGTGATTGAGC
>GX0531_concensus           GAGTAGGGGAAAGGGAATTGTGGTTGGACAACCGAGTTCTGAGAAAGGCAGTGATTGAGC
>GX0540_concensus           GAGTAGGGGAAAGGGAATTGTGGTTGGACAACCGAGTTCTGAGAAAGGCAGTGATTGAGC
HG03385_DEL2_hom            GAGTAGGGGAAAGGGAATTGTGGTTGGACAACCGAGTTCTGAGAAAGGCAGTGATTGAGC       B-A
>GX0403-C_D2_concensus      GAGTAGGGGAAAGGGAATTGTGGTTGGACAACCGAGTTCTGAGAAAGGCAGTGATTGAGC
>GX0300-C_D2_concensus      GAGTAGGGGAAAGGGAATTGTGGTTGGACAACCGAGTTCTGAGAAAGGCAGTGATTGAGC
>GX0440-C_D2_concensus      GAGTAGGGGAAAGGGAATTGTGGTTGGACAACCGAGTTCTGAGAAAGGCAGTGATTGAGC
                               ** *** ********************** ***************************

                                   B                         B           B  X
GYPE_F3-R3                  AGCTGAATTCCATCCATTTGGTGGGTAAGGACCTCAGAGTATTCAAAGTATCAAATGGAA       2029
GYPE-GYPB_F3-R3             AGCTGAACTCCATCCATTTGGTGGGTAAGGACCCCAGAGTATTCAAAGTATCAAATGGAA       2035
GYPB-GYPA_F3-R3             AGCTGAACTCCATCCATTTGGTGGGTAAGGACCCCAGAGTATTCAAAGGATCAAATGGAA       2026
GM19140_NORM_concensus      AGCTGAACTCCATCCATTTGGTGGGTAAGGACCCCAGAGTATTCAAAGGATCAAATGGAA       B-A
>GX0387_concensus           AGCTGAACTCCATCCATTTGGTGGGTAAGGACCCCAGAGTATTCAAAGGATCAAATGGAA
>GX0531_concensus           AGCTGAACTCCATCCATTTGGTGGGTAAGGACCCCAGAGTATTCAAAGGATCAAATGGAA
>GX0540_concensus           AGCTGAACTCCATCCATTTGGTGGGTAAGGACCCCAGAGTATTCAAAGGATCAAATGGAA
HG03385_DEL2_hom            AGCTGAACTCCATCCATTTGGTGGGTAAGGACCCCAGAGTATTCAGAGGATCAAATGGAA       B-A
>GX0403-C_D2_concensus      AGCTGAACTCCATCCATTTGGTGGGTAAGGACCCCAGAGTATTCAGAGGATCAAATGGAA
>GX0300-C_D2_concensus      AGCTGAACTCCATCCATTTGGTGGGTAAGGACCCCAGAGTATTCAGAGGATCAAATGGAA
>GX0440-C_D2_concensus      AGCTGAACTCCATCCATTTGGTGGGTAAGGACCCCAGAGTATTCAGAGGATCAAATGGAA
                            ******* ************************* *********** ** ***********

                                       X          X   X         BB
GYPE_F3-R3                  TGTGACTGAAGAGTTATCTTGCTGATCTTACCCAGGAGGACTGTCCCCTAGGCAAGGGGA       2089
GYPE-GYPB_F3-R3             TGTGACTGAAGTGTTATCTTGCTGATGTTACCCAGGAAAACTGTCCCCTAGGCAAGGGGA       2095
GYPB-GYPA_F3-R3             TGTGACTGAAGAGTTATCTTGCCGATCTTACCCAGGAAGACTGTCCCCTAGGCAAGGGGA       2086
GM19140_NORM_concensus      TGTGACTGAAGAGTTATCTTGCCGATCTTACCCAGGAAGACTGTCCCCTA----------       B-A
>GX0387_concensus           TGTGACTGAAGAGTTATCTTGCCGATCTTACCCWGGAAGACTGTCCCCTAGGCAAGGG-A
>GX0531_concensus           TGTGACTGAAGAGTTATCTTGCCGATCTTACCCAGGAAGACTGTCCCCTAGGCAAGGGGA
>GX0540_concensus           TGTGACTGAAGAGTTATCTTGCCGATCTTACCCAGGAAGACTGTCCCCTAGGCAAGGGG
HG03385_DEL2_hom            TGTGACTGAAGAGTTATCTTGCCGATCTTACCCAGGAAGACTGTCCCCTAGGCAA-----       B-A
>GX0403-C_D2_concensus      TGTGACTGAAGAGTTATCTTGCCGATCTTACCCAAGAAGACTGTCCCCTAGGCAAGGGGA
>GX0300-C_D2_concensus      TGTGACTGAAGAGTTATCTTGCCGATCTTACCCAGGAAGACTGTCCCCTAGGCAAGGGGA
>GX0440-C_D2_concensus      TGTGACTGAAGAGTTATCTTGCCGATCTTACCCAGGAAGACTGTCCCCTAGGCAAGGGGA
                            *********** ********** *** **********  ***********

GYPE_F3-R3                  CACCAAACACAATGAGGCATGGGATAAGGATTTCGTGATGAGAATCGGGCAGAACTG          2146
GYPE-GYPB_F3-R3             CACCAAACACAAAGAGGCACGGGATAAGGATTTCGTGACGAGAATTGGGCAGAACTG          2152
GYPB-GYPA_F3-R3             CACCAAACACAAAGAGGCATGGGATAAGGATTTCAAGATGAGAGTTGGGCAGAACTG          2143
GM19140_NORM_concensus      ---------------------------------------------------------          B-A
>GX0387_concensus           CACCAAACACAAAGAGG----------------------------------------
>GX0531_concensus           CACCAAACACAAAGAGG----------------------------------------
>GX0540_concensus           ACACCAAACACAAAGAGG---------------------------------------
HG03385_DEL2_hom            ---------------------------------------------------------          B-A
>GX0403-C_D2_concensus      CACCAAACACAAAGA------------------------------------------
>GX0300-C_D2_concensus      CACCAAACACAAAGA------------------------------------------
>GX0440-C_D2_concensus      CACCAAACACA----------------------------------------------
```

<= DEL2_GYPEBAs_R3
(5'-CAGTTCTGCCCAACTCTCATCTT-3')

Supplementary File 6: GYP boundaries from Leffler et al (Science 2017)

>5’-GYPE
GTTCTCATTCTGAGTTCACATGAGATCTAGTTTTTTTTTTTAATGTTATTATTATTATACTTTAAGTTTTAGGGTACATGTGCACAACGTGCAGGTTTGTT
>GYPA-3’
AAGGTAAAGGGGTAGAAATATACATTCAACGCAAATGCAACCAGAAGCAAGCAGGAGTAGCTTTTCTTTTATTTTTTTTTAATTTTATTATTATTGTACTT

GRCh37	GRCh38
**4:144,706,830**	**4:143,785,677**
**4:145,069,066**	**4:144,147,913**

GYP genic regions

>GYPE_GRCh37	>GYPE_GRCh38
4:144792020-144826716	4: 143870864-143905559
>GYPB_GRCh37	>GYPB_GRCh38
4:144917257-144940492	4:143996104-144019339
>GYPA_GRCh37	>GYPA_GRCh38
4:145030457-145061904	4:144109303-144140751

DEL2 breakpoint was given as 206,000 bases from the 5' end of the GYP region
GRCh37 **4:144,706,830** 4:144706830
GRCh38 **4:143,785,677** 4:143785677

**>DEL1_breakpoints**_GRCh37	**>DEL1_breakpoints**_GRCh38	**>DEL2_breakpoints**__GRCh37	**>DEL2_breakpoints**__GRCh38
**4:144835169-144835279**	**4:143914016:143914126**	4:144792078:144792207	**4:143870925:143871054**
**4:144945407-144945517**	**4:144024254:144024364**	4:144912872-144913001	**4:143991718:143991848**
**4:145066753:145066863**	**4:144145600:144145710**	4:145016127-145016256	**4:144094974:144095103**

```
>DEL1-DEL2_breakpoints
>GYPB-GYPA
TACATGAGTATATGTGTGAAACTGTCACTCAAATCAAGACATAGAACATTTCAGATGTATTCCAGAGATCATGGTGGATGGGAGGCAGGACTGGATTGCAGCTCCCACTTG
>GYPE-GYPB
TACATGAGTATATGTGTGAAACTGTCACTCAAATCAAGACATAGAACATTTCAGATGTATTCCAGAGATCATGGTGGATGGGAGGCAGGACTGGATTGCAGCTCCCACTTG
>5'-GYPE
CAGGATACTTACAATCATGACCAAAGGGGAAACAAACACATCTTTCTTACATAGTGGCAGGAAGGAGAAGAATGAGAGCTGAGTGAAGGGGGAAGCTCCTTTATAAAACTATCAGATTATGTGAGAATTTA
>GYPE-GYPB
TAGGATACTTACAATCATGACCAAAGGGGAAACAAACACATCTTTCTTACATAGTGGCAGGAAGGAGAAGAATGAGAGCTGAGTGAAGGGGGAAGCTCCTTTATAAAACTATCAGATTATGTGAGAATTTA
>GYPB-GYPA
CAGGATACTTACAATCATGACCAAAGGGGAAACAAACACATCTTTCTTACATAGTGGCAGGAAGGAGAAGAATGAGAGCTGAGTGAAGGGGGAAGCTCCTTTATAAAACTATCAGATTATGTGAGAATTTA
```
